# Acute Metabolic Effects in Brazilian A‑29 Fighter Pilots by NMR-Based Metabolomics

**DOI:** 10.1021/acs.jproteome.5c00129

**Published:** 2025-07-21

**Authors:** Roberta Verissimo França de Oliveira, Grace Barros de Sá, Alanny Cristine dos Santos Pinheiro, Antonia Claudia Jácome da Câmara, Palmielly Diógenes, Adriano Percival Calderaro Calvo, Laila Ribeiro Fernandes, Luísa Soares da Silva, Rafael Loureiro Simões, Verônica Morandi, Gilson Costa dos Santos, Paulo Farinatti

**Affiliations:** † Laboratory of Metabolomics (LabMet), IBRAG/Department of Genetics, 162388 State University of Rio de Janeiro (UERJ), Rio de Janeiro, Rio de Janeiro 20950-003, Brazil; ‡ Laboratory of Physical Activity and Healthy Promotion (Labsau), Institute of Physical Education and Sports, State University of Rio de Janeiro (UERJ), Rio de Janeiro, Rio de Janeiro 20550-900, Brazil; § Integrated Laboratory of Clinical Analysis (LIAC), 28123 Federal University of Rio Grande do Norte (UFRN), Natal, Rio Grande do Norte 59010-180, Brazil; ∥ Laboratory of Endothelial Cell Biology & Angiogenesis/Department of Cell Biology, IBRAG/Department of Cell Biology, 28130 University of Rio de Janeiro (UERJ), Rio de Janeiro, Rio de Janeiro 20550-900, Brazil; ⊥ Laboratory of Cellular and Molecular Pharmacology/Department of Cell Biology, IBRAG/Department of Cell Biology, University of Rio de Janeiro (UERJ), Rio de Janeiro, Rio de Janeiro 20550-900, Brazil; # Laboratório de Biologia de Parasitos e de Doença de Chagas, Departamento de Análises Clínicas e Toxicológicas, CCS, UFRN, Natal, Rio Grande do Norte 59012-570, Brazil; ○ 385538 Brazilian Air Force University (UNIFA), Military Human Performance Post-Graduation, Rio de Janeiro, Rio de Janeiro 21740-002, Brazil; & Institute of Aerospace Medicine Brigadier Doctor Roberto Teixeira (IMAE), Rio de Janeiro, Rio de Janeiro 21740-001, Brazil

**Keywords:** G-force, acute metabolism, blood, urine, saliva, military aviation, fighter pilots, NMR, metabolomics, A-29 jet

## Abstract

Operating an aircraft imposes significant physical and mental demands on pilots, particularly those in military aviation. These challenges include circadian disruptions, irregular working hours, and exposure to G-forces. This study investigates the acute metabolic effects of flight in the A-29 fighter pilots of the Brazilian Air Force (FAB). Blood, urine, and saliva samples were collected from 32 pilots, trainees (*n* = 12; aged 23–26 years) and instructors (*n* = 20; aged 25–41 years), immediately before and after flights. Assessments included anthropometric measurements, complete blood count (CBC), circulating endothelial cells (CECs), coagulogram, lipidogram, urinalysis, and nuclear magnetic resonance (NMR)-based metabolomics. After flights, trainees showed a 12% increase in the number of segmented neutrophils, while instructors exhibited a 15% increase in the number of lymphocyte counts. Serum lactate levels decreased in both groups (23% in trainees and 12% in instructors). Salivary glucose increased by 49% in trainees, whereas instructors demonstrated decreases in metabolites such as choline (23%) and lactate (15%). Urinary trigonelline levels increased by 53% in instructors. The observed changes were more pronounced in instructors vs trainees, indicating a degree of metabolic adaptation associated with greater flight experience. These findings highlight NMR-based metabolomics as a valuable tool for monitoring acute metabolic changes in fighter pilots.

## Graphical abstract

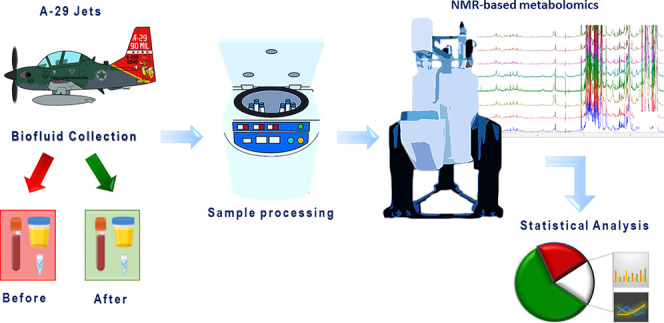

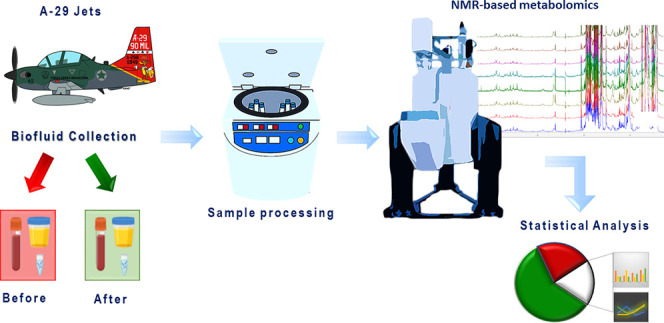

## Introduction

Recent advancements in aviation technology have enabled the development of aircraft capable of withstanding significant gravitational forces. Under such conditions, pilot performance plays a critical role in preventing accidents.^1–3^ Military pilots face numerous challenges, including circadian disruptions, insufficient sleep, high physical and mental demands, and repeated exposure to gravitational forces, all of which contribute to considerable physiological strain.^2^

The accelerations experienced during combat flights can have severe adverse effects on pilot health. Research has shown that stress-induced cardiorespiratory disorders significantly impair physiological function.^4,5^ Prolonged exposure to positive gravitational forces (+Gz) has been linked to circulatory dysfunction, alveolar collapse, ventilatory impairment, hypoxia, ischemia, muscle damage, bruising, petechiae, temporary visual disturbances, and metabolic alterations.^6–9^

Given these stressors, the study of human–machine interactions is essential to mitigate human error. Despite its importance, research focusing on pilot health and well-being remains limited, hindering efforts to address the acute and chronic effects of +Gz exposure.^10^ Tolerance to +Gz is influenced by factors such as sex,^11,12^ age, body composition, and anthropometric characteristics.^13,14^ Existing countermeasures, such as anti-G suits, positive pressure breathing, and the Anti-G Strain Maneuver (AGSM), are widely utilized.^10^ However, there remains an urgent need to develop more effective strategies to enhance tolerance to gravitational forces.^15–17^

To improve our understanding of the physiological demands imposed by combat flights, metabolomics has emerged as a valuable research tool. Studies in animal models have identified +Gz-induced metabolic fluctuations affecting pathways such as fatty acid β-oxidation, glycerophospholipid metabolism, and purine metabolism.^18^ “Omics” technologies, encompassing proteomics, genomics, transcriptomics, and metabolomics, facilitate the identification and characterization of biomolecules and signaling pathways.^19^ Among analytical methods, nuclear magnetic resonance (NMR) spectroscopy is notable for its high reproducibility, minimal sample preparation requirements, and capacity for direct quantification.^20,21^

A recent study by our group^22^ demonstrated that experienced fighter pilots exhibit more pronounced metabolic adaptations compared to their less experienced counterparts, with metabolic profiles resembling those of control groups. Pilots with fewer flight hours exhibited significant reductions in white blood cells (−13%), neutrophils (−15%), and lymphocytes (−20%). NMR-based metabolomics identified variations in amino acids (e.g., histidine and glutamine), energy substrates (e.g., glucose and lactate), and microbiome-associated metabolites (e.g., methylamine, isobutyrate). These findings underscore the importance of metabolic monitoring to mitigate health complications over the course of pilots’ careers.

Since chronic adaptations result from repeated acute exposures, investigating the physiological responses of fighter pilots could yield valuable insights. Such research could elucidate hematological, vascular, and metabolic factors that contribute to chronic adaptations associated with continuous acute exposure to +Gz forces. This knowledge could inform the development of targeted interventions and training programs to minimize the physiological impacts of jet flight. Accordingly, the present study investigated serum, saliva, and urine samples from A-29 fighter pilots of the Brazilian Air Force (FAB) immediately before and after mission flights. We hypothesized that trainees and instructors display different acute metabolic flexibilities when piloting A-29 jets.

## Methods

### Participants

The study included pilots who met the fitness criteria established by the FAB during their annual health inspections. All participants were assessed for flight readiness and had previously completed a physiological adaptation program at the Institute of Aerospace Medicine. Additionally, each pilot had successfully passed the FAB’s routine health and physical fitness evaluations. None of the participants had a documented history of cardiovascular disease, dyslipidemia, or hormonal disorders. In addition, we collected information about positions in A-29, period of flight, physical activity, smoking, hypertension, diabetes, and regular use of supplements, as shown in Table S6.

A total of 96 biofluid samples (blood, urine, and saliva) were collected from 32 A-29 pilots stationed at Natal Air Base (BANT, RN, Brazil) before and after flights conducted between October 24 and 28, 2022. The pilots, all male, aged 23 to 41 years, participated in two types of missions: combat and reconnaissance. During these flights, accelerations did not exceed +4Gz, and the duration of each flight was approximately 1 h.

Two-seater A-29 aircraft were used, and pilots were categorized into two groups, trainees and flight instructors, to maintain regular operational routines. All participants provided written informed consent in compliance with approval from the Research Ethics Committee (CEP) (Opinion No. 2,528,903), following the ethical guidelines established by Resolution No. 466/2012 of the Brazilian National Health Council. Biofluid samples were collected both before and immediately after the flights (approximately 20 min postflight) in a designated room located at the end of the runway. Despite differences, both groups followed identical protocols for biofluid collection and similar diet, physical training, and flight protocols. Both also fell within the same reference ranges for Brazilian male laboratory tests, which account for factors like age and exposure to various agents.^23^

### Blood Collection

Blood samples were collected from the pilots via venipuncture. Plasma intended for blood count analysis was drawn into tubes containing liquid tripotassium ethylenediamine tetra-acetic acid (K3EDTA) anticoagulant, while serum for biochemical and NMR-based metabolomics analysis was collected in vacuum tubes containing a gel separator and clot activator. Blood samples designated for coagulogram analysis were collected in sodium citrate tubes (Biocon, Belo Horizonte, MG, Brazil). Samples were transported on dry ice and maintained at −80 °C until further NMR analysis.

Plasma was separated by centrifugation at 1000*g* for 10 min at 4 °C and analyzed using an ABX Micros 60 (HORIBA). Serum was separated by centrifugation at 2000*g* for 15 min and 4 °C and analyzed using a Labmax Plenno (LABTEST). For coagulogram analysis, blood samples were centrifuged at 1500*g* for 15 min at room temperature. After separation, 1.5 mL of serum supernatant was transferred to microtubes and stored at −80 °C.

### Saliva Collection

Saliva samples were collected in a 50 mL Falcon tube, specifically for this purpose. A total of 2 mL of unstimulated saliva was collected from the participants immediately before and after the flight. The samples were centrifuged at 12,000*g* and 4 °C for 15 min, and the supernatant was then stored at −80 °C, following the same procedure as previously described.

### Urine Collection

Urine samples were collected immediately before and after the flight, concurrent with blood sample collection. Following collection, the samples were stored on ice. For analysis, 12 mL of urine was utilized, and urinary sediment was obtained by centrifuging the samples at 3,000*g* for 10 min at room temperature. Reagent strips were employed for manual colorimetric analysis to rapidly and semiquantitatively assess levels of glucose, bilirubin, ketone bodies (acetoacetic acid), specific gravity, blood, pH, protein, urobilinogen, nitrite, and leukocyte esterase.

The urinary sediment was prepared for microscopic examination, during which leukocytes, red blood cells, epithelial cells, casts, crystals, salts, and other structures were counted across 10 fields at 100× and 400× magnification. A result of 0.1 p/c indicates that one structure was observed in 10 fields. Additionally, 1.5 mL of urine supernatant was transferred to microtubes and stored at −80 °C, following the procedures previously described.

### Quantification of Circulating Endothelial Cells (CECs)

Collected peripheral blood was placed into 4 mL tubes containing K3EDTA as an anticoagulant. Then, 150 μL of blood was transferred to 1.5 mL microtubes, mixed with TransFix (Cytomark, Buckingham, UK), and stored at 4 °C until analysis. The identification of circulating endothelial cells (CECs) was performed by flow cytometry through an adaptation of a previously described protocol.^24^ Briefly, and prior to staining with antibodies, 100 μL of whole blood was incubated with Human TruStain FcX (Biolegend, San Diego, CA) and mouse serum (Merck, Darmstadt, Germany), according to the manufacturer’s recommendation. Then, the staining was performed with the following conjugated mouse antihuman antibodies (all purchased from Biolegend): CD45 FITC (cat. no. 368508), CD34 APC (cat. no. 343510), CD146 PerCP-Cy 5.5 (cat. no. 361010), CD 133 PE (cat. no. 372804), and CD106 PE (cat. no. 30506) and their respective control isotypes bearing the same fluorochrome.

From this stage onward, the samples were protected from light. After incubating at room temperature for 30 min, the samples were subjected to red blood lysis with RBC lysis buffer (Biolegend) for 15 min at room temperature and vortexed, and the acquisitions (1 × 10^6^ events per condition) were carried out on a BD Accuri C6 Plus flow cytometer. Analyses were performed using dedicated cytometer software (C6 Plus analysis software, version 1.0.27.1).

### NMR-Based Metabolomics

For the NMR-based metabolomics of serum, sample processing was conducted according to the method described by dos Santos Pinheiro et al.^22^ For saliva samples, 100 μL of 300 mM sodium phosphate buffer (pH 7.4) in 100% D_2_O, containing 1.2 mM TSP-*d*_4_ (3-(trimethylsilyl)­propionic acid-2,2,3,3-*d*_4_ sodium salt, Sigma-Aldrich, Brazil), was added to 500 μL of saliva, and the mixture was gently homogenized and transferred to 5 mm NMR tubes. For urine analysis, 250 μL of urine was combined with 500 μL of 75 mM sodium phosphate buffer in 100% D_2_O (pH 7.4) containing 0.15 mM TSP-*d*_4_, homogenized, and subsequently transferred to 5 mm NMR tubes. TSP-*d*_4_ was used as an internal reference.

Proton (^1^H) NMR spectra were acquired by using a Bruker 500.13 MHz Avance III spectrometer (Bruker Biospin, Rheinstetten, Germany). For urine samples, spectra were obtained using the zgesgp pulse sequence, while for saliva and serum, the Carr–Purcell–Meiboom–Gill (CPMG) pulse sequence was employed to suppress macromolecular signals. CPMG parameters included a D2O of 0.001 s and a loop counter L4 of 32. Acquisition temperature was set to 298 K with an FID size of 64 K and 1024 scans for serum and saliva and 512 scans for urine. Spectral width was 20 ppm, with a transmitter frequency offset of 4.70 ppm, receiver gain of 203 dB, an acquisition time of 3.27 s, and a relaxation delay of 2 s.

Metabolite assignments for all biofluids were performed by using ^1^H–^1^H total correlation spectroscopy (TOCSY) and ^13^C–^1^H heteronuclear single quantum coherence (HSQC) experiments. Postacquisition spectra were processed using MestReNova version 14.2.1-27684, incorporating zero-filling, phase and baseline correction, and calibration. Spectra were binned at 0.02 ppm intervals using the average sum method and normalized by TSP-*d*_4_.

Two-dimensional spectra were processed with TopSpin, transformed into text files, and uploaded to the COLMARm database^25^ for metabolite assignment. Peak reports were generated by COLMARm with the session IDs: 3307-SJKHh6VhYX for serum, 165-8ABuybBDP8 for saliva, and 134-GfT6JWKsU1 (TOCSY) and 3154-Hqr3sX2JxR (HSQC) for urine, accessible at COLMARm. Default parameters were used for classical peak picking without peak fitting or referencing. Following the processing of one- and two-dimensional NMR spectra, several metabolites present in the pilots’ biofluids were identified and assigned by analyzing chemical shifts and comparing them with standard NMR spectra from the Biological Magnetic Resonance Data Bank (BMRB)^26^ and the Human Metabolome Database (HMDB).^27^

The NMR-based metabolomics data have been deposited in the MetaboLights repository (https://www.ebi.ac.uk/metabolights/editor/study/REQ20250222208808) with the data set identifier REQ20250222208808.

### Statistics

The bucket tables were uploaded to MetaboAnalyst^28^ for Principal Component Analysis (PCA) and GraphPad Prism version 10.1.0 (GraphPad Software, Boston, MA, USA) for univariate analysis. Normality and log-normality tests were conducted using the Shapiro–Wilk, Anderson–Darling, and D’Agostino and Pearson tests. Descriptive statistics were reported as medians (minimum–maximum). Subsequently, the chi-Square test was used to determine whether there was association between categorical variables; the Wilcoxon matched-pairs signed-rank test was performed with multiple comparisons assessed using the two-stage step-up method of Benjamini, Krieger, and Yekutieli, along with the False Discovery Rate (FDR) approach, targeting a desired FDR (Q) of 5%. In all cases, the significance level was set at *p* ≤ 0.05.

## Results

To investigate the potential acute effects associated with combat flight experience, trainees and flight instructors were analyzed separately based on their status within the Air Force at Natal Air Base, as summarized in Table 1. Trainees were, on average, approximately six years younger than instructors and had 895 fewer total flight hours and 889 fewer combat flight hours. Additionally, the rate of perceived exertion (RPE) during flights was higher among trainees compared to instructors. There was also a significant difference in the number of trainee pilots who performed physical activities over instructors (Table S6).

**Table 1:** Characteristics of 32 Pilots^a^

Variable	Trainees (*n* = 12)	Instructors (*n* = 20)	*p*-value
Age [y]^c^	**23.8 ± 1.07**	**31.3 ± 4.14**	**<0.001** ^b^
Weight [kg]	81.5 (71–95)	82 (67–102.1)	0.94
Height [m]	**1.8 (1.71–1.87)**	**1.77 (1.64–1.84)**	**0.02** ^b^
BMI [kg/m^2^]	24.8 (23.15–28.41)	26.545 (21.15–30.48)	0.08
Total flight time [h]	**251 (150–70)**	**1300 (950–3000)**	**<0.001** ^b^
Combat flight time [h]	**142.5 (100–225)**	**1085 (800–2300)**	**<0.001** ^b^
Weekly flight frequency	**4 (3–7)**	**6 (5–8)**	**<0.001** ^b^
Sleep quality [score]	8 (5–10)	8.5 (3–10)	0.37
Sleep [h]	6.25 (4.5–8)	7 (4–8.5)	0.08
Flight RPE [0–10]	**5.5 (2–10)**	**3 (2–7)**	**0.01** ^b^

a Data presented as median (min–max). RPE: Rate of perceived exertion.

b Significant difference between trainees and instructors (*p* < 0.05).

c Parametric data, mean and standard deviation.

Following the characterization of the pilot groups, we assessed their blood profiles, as summarized in Table 2. A comprehensive analysis was conducted, including a complete blood count, lipid profile, leukogram, blood glucose levels, coagulogram, and mature circulating endothelial cells (CECs), immediately before and after flights. The results indicate that trainees experienced a reduction in mean corpuscular volume (MCV) (−1.14%, *p* = 0.03) and an increase in segmented neutrophils after flights (+35.1%, *p* < 0.01). In contrast, instructors exhibited a significant increase in the number of segmented lymphocytes (+15.11%, *p* = 0.007). No significant acute differences in CEC counts were observed between instructors and trainees (*p* > 0.05), as shown in Figure S1a. However, a significant positive linear correlation was identified between CEC counts and the number of days since the last flight (*r* = 0.34, *p* = 0.04), as shown in Figure S1b. The complete data set is available in the Supporting Information (Table S1).

**Table 2:** Trainees and Instructors Showed Increased Immune Cells after Acute Flights^a^

	Trainees (*n* = 12)			Instructors (*n* = 20)		
Variable	Before flight	After flight	*p*-value	Before flight	After flight	*p*-value
Red cells (×10^6^/mm^3^)	5.15 (4.18–5.58)	5.09 (4.26–5.67)	0.20	5.11 (4.33–5.73)	5.01 (4.29–5.54)	0.19
Hemoglobin (g/dL)	15.3 (14.1–16.9)	15.3 (14.1–17.1)	0.22	15.7 (13.4–16.4)	15.4 (14.0–16.6)	0.18
Hematocrit (%)	45.1 (38.1–49.0)	44.0 (39.8–48.8)	0.15	45.1 (40.3–47.3)	44.8 (40.0–47.0)	0.55
Mean corpuscular volume (fL)	**88.0 (81.7–93.0)**	**87.0 (81.5–93.0)**	**0.03** ^*^	89.0 (79.6–93.0)	89.0 (78.9–94.0)	0.55
Mean corpuscular hemoglobin (pg)	30.8 (27.2–289.0)	30.6 (27.3–33.2)	0.72	31.0 (26.9–33.7)	30.9 (27.3–32.8)	0.49
MCH (g/dL)	34.6 (33.3–36.0)	35.0 (33.0–35.5)	0.50	34.9 (32.9–341.0)	35.0 (32.1–35.7)	0.72
RDW (%)	12.5 (11.8–12.9)	12.5 (12.1–13.0)	0.88	12.7 (12.3–13.3)	12.8 (12.2–13.0)	0.84
Number of platelets (mm^3^ × 1000)	212 (165–306)	223 (162–335)	0.58	250 (112–412)	257 (119–433)	0.59
Average platelet volume (fL)	11.1 (10.7–11.3)	10.9 (10.7–11.2)	0.13	11.1 (10.3–11.5)	11.0 (10.0–11.3)	0.18
Number of leukocytes (mm^3^)	6700 (4400–9200)	7600 (1580–9600)	0.07	6650 (4300–9700)	6850 (1190–8500)	0.07
Rod cells (mm^3^)	0 (0 −140)	0 (0–96)	>0.99	0 (0–0)	0 (0–85)	0.06
Segmented (mm^3^)	**3801 (2184–5888)**	**5135 (2842–13746)**	<0.01*	34925 (1677–4964)	3318 (1739–7138)	0.65
Eosinophils (mm^3^)	180 (74–448)	169 (0–532)	0.85	147 (68–660)	150 (0–518)	0.60
Basophils (mm^3^)	0 (0–44)	0 (0–0)	>0.99	0 (0 −73)	0 (0–85)	>0.99
Lymphocytes (mm^3^)	2251 (704–2961)	1610 (990–2464)	0.27	**2157 (1081–5723)**	**2483 (830–6307)**	0.007*
Monocytes (mm^3^)	415 (222–621)	451 (90–632)	>0.99	429 (138–972)	450 (106–810)	0.23
Blood glucose (mg/dL)	79 (62–111)	84 (71–102)	0.06	81.7 (64–95.8)	82.60 (53–110)	0.92
Total cholesterol (mg/dL)	152.5 (85.0–235.8)	156.9 (104.0–243.5)	0.39	181.5 (147.0–311.9)	196.4 (151.0–320.6)	0.69
Cholesterol HDL (mg/dL)	51.5 (25–74)	52 (27–76)	0.24	52 (37–68)	56 (39–71)	0.72
Cholesterol LDL (mg/dL)	86.0 (46.0–182.6)	86.0 (21.0–187.6)	0.64	110.7 (78.0–217.4)	118.9 (80.0–229.5)	0.56
Cholesterol VLDL (mg/dL)	14.0 (9.0–27.9)	15.5 (7.0–28.5)	0.62	19.5 (11.7–40.0)	19.2 (9.6–35.0)	0.49
Cholesterol non-HDL (mg/dL)	100 (60–138)	102 (28–167)	0.41	128 (94–212)	128 (94–204)	0.68
Triglycerides (mg/dL)	64.5 (30.0–139.6)	72.4 (26.0–142.7)	0.96	92 (48–247)	94 (42–187)	0.38
Creatine kinase (U/L)	220 (72–607)	212 (67–640)	0.49	179 (75–737)	181 (82–749)	>0.99
PTT (s)	16.1 (12.1–34.0)	14.80 (12.0–43.7)	0.73	14.4 (12.0–25.0)	14.80(12.5–22.5)	0.83
PPT (INR)	1.2 (1.0–2.6)	1.1 (1.0–3.4)	0.64	1.0 (1.00–1.83)	1.1 (1.0–1.7)	0.95
PPT (%)	64.7 (16.1–100.0)	84.0 (11.5–100.0)	0.84	91.8 (25.8–100.0)	82.9 (30.4–100.0)	0.72
aPPT (s)	33.1 (26.2–46.9)	31.8 (26.2–51.9)	0.38	29.5 (25.4–38.6)	31.8 (24.5–44.0)	0.07
aPPT (p/c)	1.10 (0.87–1.56)	1.06 (0.87–1.73)	0.38	0.98 (0.85–1.29)	1.06 (0.82–1.47)	0.08
Fibrinogen (mg/dL)	221 (70–308)	211 (75–359)	0.52	209 (95–387)	231 (101–399)	0.19
CECs (N cells/mL)	10 (0–25)	8 (0–25)	>0.99	15 (0–35)	10 (0–40)	0.77

a Data of hemograms, lipidograms, coagulograms, and mature circulating endothelial cells (CECs) in trainees and instructors before and after flights. Data expressed as median (min–max). MCH, Mean Corpuscular Hemoglobin; RDW, Red Blood Cell Distribution Width; PTT, Partial Thromboplastin Time; aPTT, Activated Partial Thromboplastin Time; CECs, Circular Endothelial Cells.

To examine the acute systemic alterations, we also employed NMR-based metabolomics on blood, saliva, and urine samples. Figure 1 presents representative images of the ^1^H NMR spectra of serum. Representative spectra for saliva and urine can be found in Figures S2 and S3, respectively. Principal Component Analysis (PCA) was used to track shifts in the metabolic profile before and after flight, as shown in Figure 2. The PCA could not display a clear separation; however, univariate analysis showed significant differences (*p* < 0.05) between groups (Figures 3–5).

**Figure 1:**
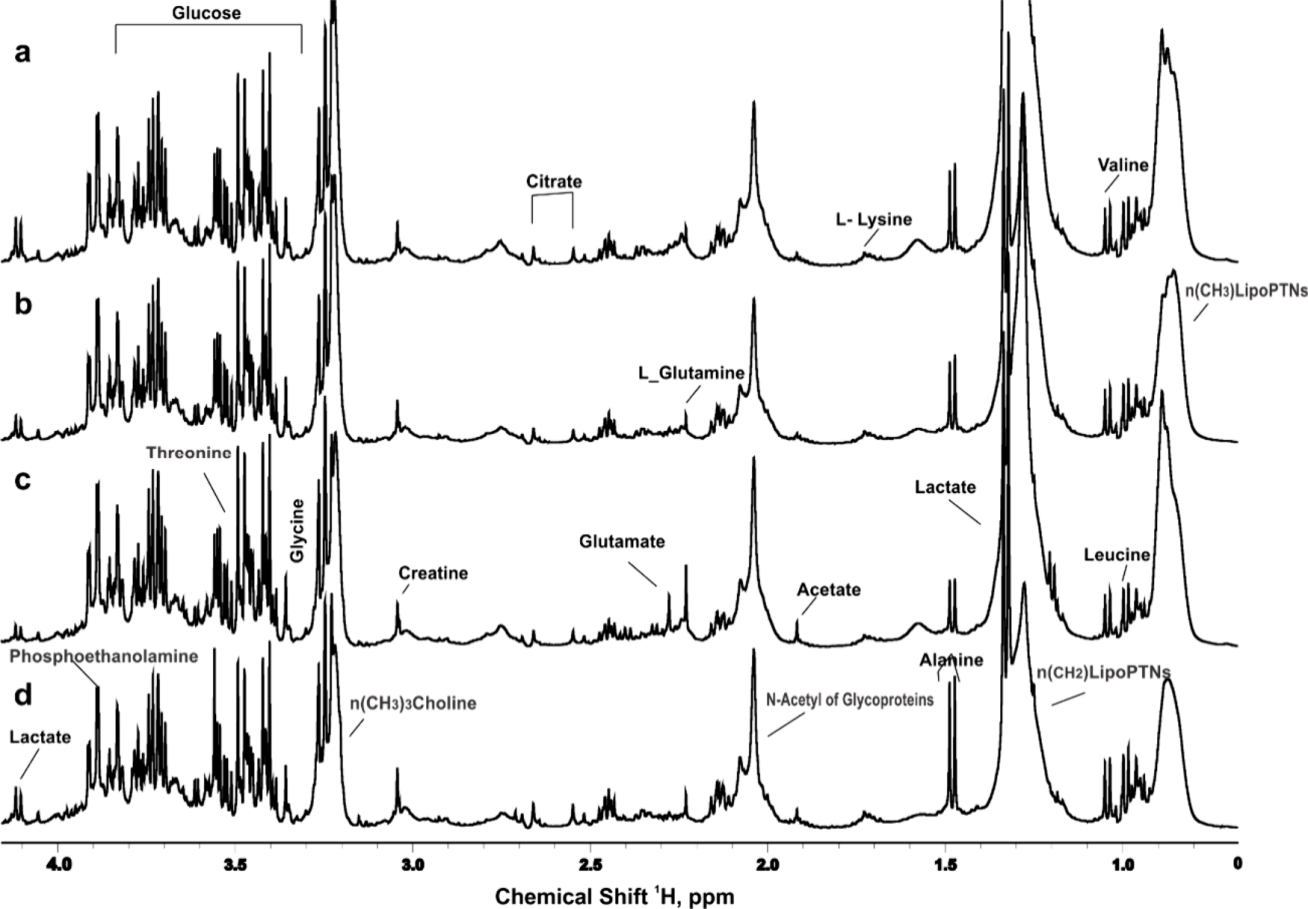
Representative image of serum 1D ^1^H NMR spectrum: (a) instructor before flight; (b) instructor after flight; (c) trainee before flight; (d) trainee after flight.

**Figure 2:**
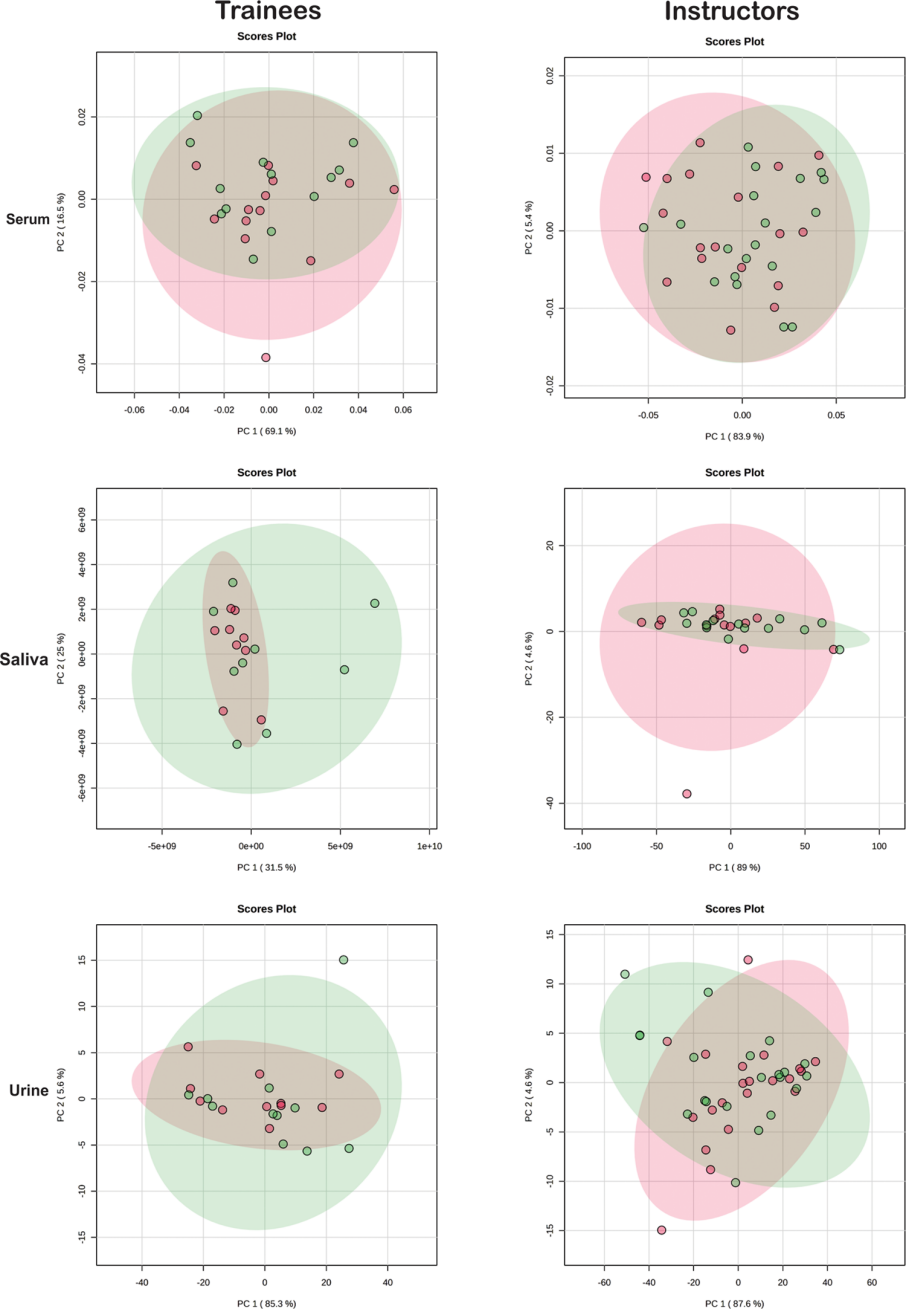
Instructors and trainee pilots demonstrated acute metabolic variation in their biofluids. Instructors and trainees demonstrated variation in their metabolic profiles of serum, saliva, and urine after flights. PCA score plots illustrate these profiles before (red circles) and after (green circles) the flight.

**Figure 3:**
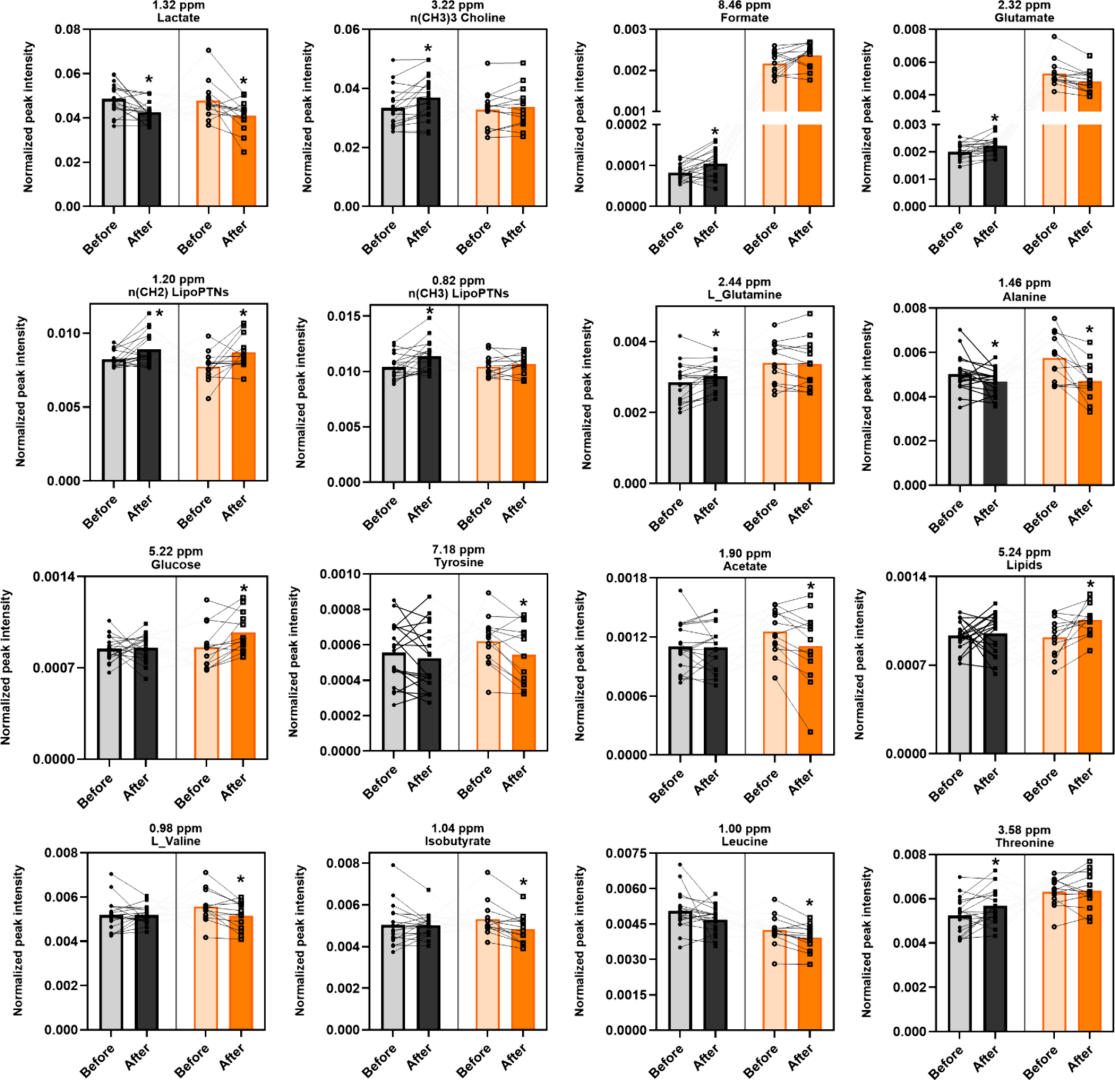
Instructors and trainees showed changes in blood metabolites after flights. Instructors (gray) and trainees (orange). Paired analysis by Wilcoxon nonparametric test. Bars represent median. *p < 0.05. The complete data, including all buckets, can be found in the Supporting Information (Table S2).

Univariate analysis revealed significant alterations in several metabolites, as depicted in Figures 3, 4, and 5. Serum lactate was the only metabolite that decreased in both groups, with reductions of 12% in trainees and 23% in instructors (*p* = 0.003 for both). Figure 3 shows that instructors exhibited increases in nearly all assigned metabolites, including choline (+12%, *p* = 0.003), formate (+34%, *p* = 0.003), glutamate (+15%, *p* = 0.006), lipoproteins (+11%, *p* = 0.006), threonine (+5%, *p* = 0.01), and glutamine (+5%, *p* = 0.01), alongside a decrease in alanine (−7%, *p* = 0.04). In contrast, trainees showed decreases in nearly all assigned metabolites, including leucine (−5%, *p* = 0.003), isobutyrate (−5%, *p* = 0.005), alanine (−21%, *p* = 0.005), l-valine (−5%, *p* = 0.01), acetate (−4%, *p* = 0.02), and tyrosine (−16%, *p* = 0.02), with increases observed in lipids (+15%, *p* = 0.008), α-glucose (+7%, *p* = 0.01), and lipoproteins (+11%, *p* = 0.04).

**Figure 4:**
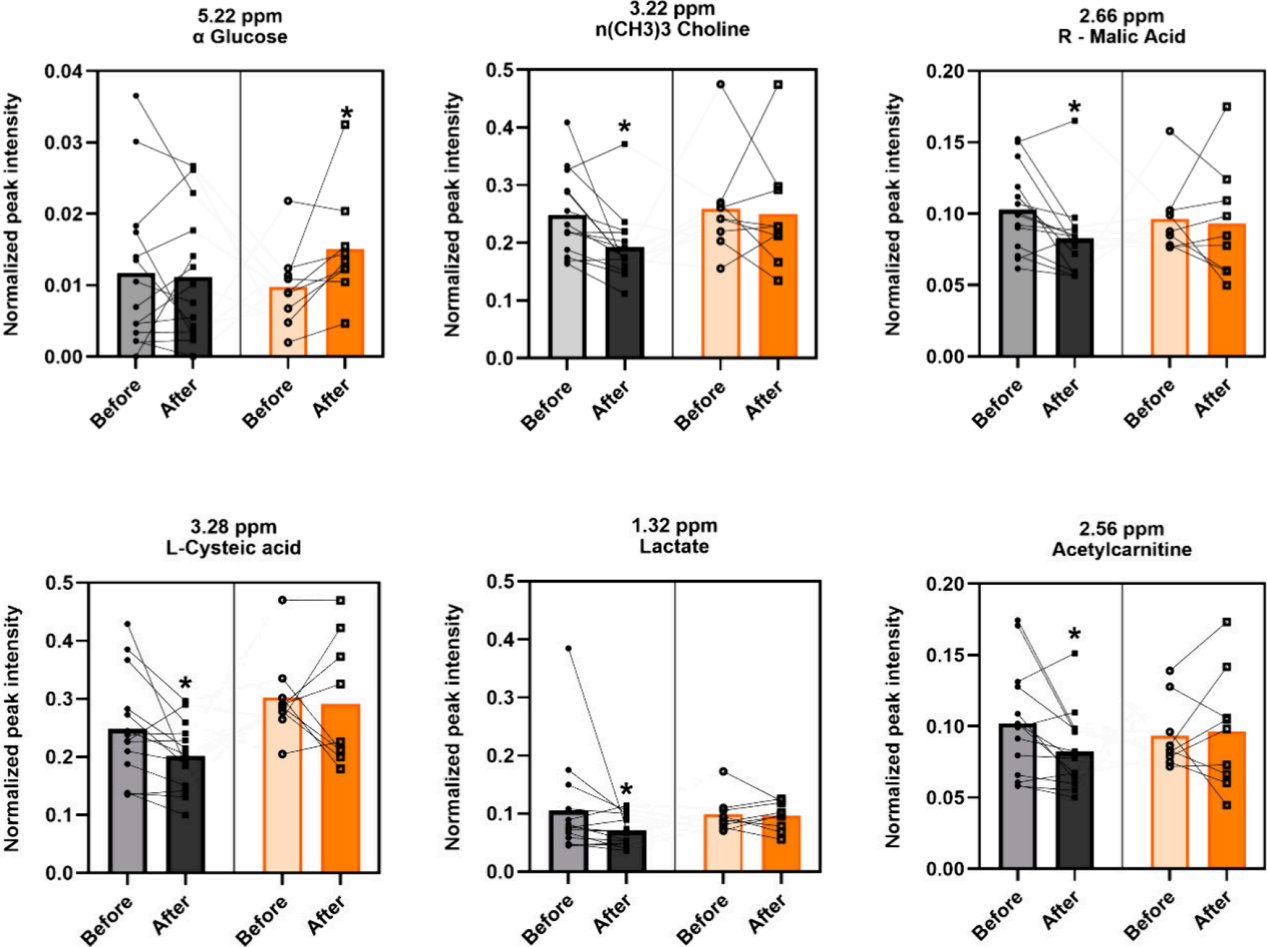
Instructors and trainees showed changes in saliva metabolites after flights. Instructors (gray) and trainees (orange). Paired analysis by Wilcoxon nonparametric. Bars represent median. *p < 0.05. The complete data, including all buckets, can be found in the Supporting Information (Table S3).

**Figure 5:**
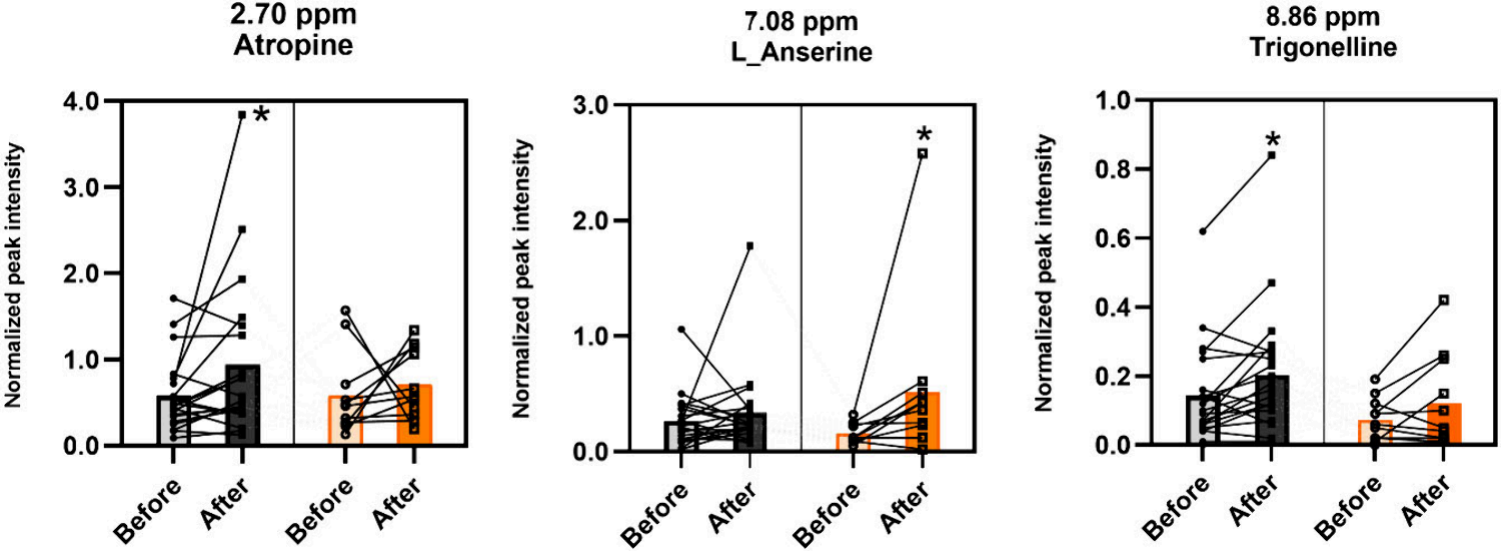
Instructors and trainees showed different changes in urine metabolites after flights. Instructors (gray) and trainees (orange). The 7.08 ppm bucket has overlapping of data L_Anserine, histamine, and 3-methylhistidine. Paired analysis by Wilcoxon nonparametric test. Bars represent median. *p < 0.05. The complete data, including all buckets, can be found in the Supporting Information (Table S4).

Saliva samples from instructors exhibited decreases in several metabolites, including choline (−23.1%, *p* = 0.007), malic acid (−16.2%, *p* = 0.017), cysteic acid (−15%, *p* = 0.02), acetylcarnitine (−23.2%, *p* = 0.035), and lactate (−15%, *p* = 0.032). Notably, glucose (+49%, *p* = 0.02) was the only metabolite that increased in the trainee’s group, as shown in Figure 4.

Figure 5 reveals that urinary metabolomics shows limited changes. Trigonelline and atropine levels increased in the instructor group (+53.8%, *p* = 0.001 and +23.9%, *p* = 0.02, respectively) after the flight. Additionally, the combined levels of l-anserine, histamine, and 3-methylhistidine increased by +200% (*p* = 0.01) in the trainee group.

In addition, we measured ratios of key serological metabolites (Figure S7), and we did a correlation analysis between significant metabolites in the 3 biofluids with key blood parameters, as shown in Figures S8, S9, and S10.

## Discussion

This study investigated serum, saliva, and urine samples from A-29 fighter pilots collected before and after flights. Exact pilot numbers are undisclosed due to national security, but reports indicate that the FAB operates 42 fighter/interceptor and 76 attack aircraft.^29^ Brazil ranks 25th globally in military strength, with the largest air force in the Southern Hemisphere, underscoring the importance of pilot health research.

Blood count data revealed no significant acute changes, only a decrease in MCV in the trainees, while the instructors had slightly higher MCV preflight but still within the reference interval for both groups,^38^ suggesting MCV shifts after age 25, which aligns with the age differences between the groups. Furthermore, flight exposure did not significantly alter leukocyte counts, but trainees showed a 35% increase in segmented leukocytes, while instructors exhibited a 15% decrease in lymphocytes. These immune changes can be linked to G-force exposure, which disrupts blood flow and homeostasis, as observed in astronauts.^30^ In addition, it is known that physical and mental stress likely influence immune system modulation,^39^ and previous studies have shown changes in leukograms and lymphocyte levels in pilots.^22^ We were unable to measure cytokines or glucocorticoids to confirm the immune system impact.

Cardiovascular diseases are common among pilots,^31^ but we did not observe significant lipid profile changes. Vascular inflammation, which contributes to atherosclerosis, is regulated by mechanical transduction, but data on hypergravity’s effect on the endothelium is limited. CECs could serve as early markers of cardiovascular damage.^32^ While we observed trends of increased CECs in instructors, further studies are needed to understand their significance.

In contrast to previous blood coagulation studies,^33,41^ no significant platelet aggregation was found. However, a 30% increase in prothrombin time (PPT) in trainees and a 10% decrease in instructors were noted. These differences were not statistically significant, but sustained G-forces (+6 to +9 Gz) in previous studies have been linked to increased platelet counts.^33^ Our study’s lower accelerations may not have been sufficient to cause significant coagulation changes.^34^

These findings suggest that, as pilots accumulate combat flight hours, they may undergo physiological adaptations to improve performance and protect against blood clots. However, over time, these adaptations could lead to chronic changes that may be harmful. Future research should examine the long-term impact of flight-induced coagulation changes^42–44^ on the health of fighter pilots.

The interesting fact was that creatine phosphokinase (CK) levels, an indirect marker of muscle damage, were elevated before flight in both groups. High CK is normal in active individuals but requires monitoring to prevent complications such as myocardial infarction or stroke.^35^

^1^H NMR-based metabolomics revealed that several metabolites were significantly changed after the flight in both the instructor and trainee groups. In the serum, both groups showed decreased lactate, suggesting oxygen supply and anti-G maneuvers helped reduce anaerobic metabolism. Essential amino acids such as leucine, isoleucine, and valine (BCAAs) decreased significantly after flight only in trainees. Some studies have reported associations between BCAAs and plasma glucose levels, where BCAAs can regulate glucose transporters.^45^ Since glucose also increased only in the trainee, this could suggest these pilots use this pathway to provide glucose to the cells, since trainee pilots would still be metabolically adapting to flight, and as they become metabolically adapted like instructor pilots, this pathway is no longer necessary.

Formate changed only in instructors’ serum, can be associated with anaerobic metabolism, and can act to inhibit cytochrome c oxidase activity.^46,47^ Another finding was that only the instructor’s group showed increased n­(CH_3_)_3_ choline, metabolized in the liver; however, both groups showed elevated lipoproteins, suggesting potential liver strain in the future.^48,49^ Unfortunately, we could not measure transaminase enzymes.

Saliva is a promising alternative to blood sampling, with lactate levels reflecting exercise intensity.^36^ In our study, trainees showed reduced saliva glucose levels, while instructors showed reductions in multiple metabolites, indicating a higher energy expenditure during flight.

Urinary metabolomics showed significant acute alterations in trainees, particularly related to histidine, such as anserine, which may reflect protective effects against oxidative stress.^37^ l-Anserine, a water-soluble carnosine-like imidazole peptide found in high levels in the muscles of salmon, tuna, and migratory fish, has several physiological benefits, including improved cognitive function, reduced blood glucose, and protection against muscle damage.^50^ In addition, l-Anserine is important during muscle contractility,^52^ and its significant increase in the trainees may be related to the fact that approximately 90% of trainees were allocated to the P1 position of the plane.

Trigonelline acts as a muscle strength enhancer and prevents fatigue, increases mitochondrial oxidative phosphorylation, and increases the activity of antioxidant enzymes. High concentrations of trigonelline are found in coffee beans, mung beans (Phaseolus aureus), and potatoes (). Interestingly, the foods directly associated with increased circulating trigonelline in the organism through dietary intake include not only coffee but also folate, fibers, and the microbiome. In addition, some studies have already reported trigonelline as a dietary agent and biomarker of metabolic health or physical fitness, as urinary trigonelline levels decrease during obesity and increase in professional athletes.^53,54^ Our results are in line with these studies since there was an increase in urinary metabolic levels of trigonelline in the group of interns. It is worth remarking that they perform more daily physical activities beyond flying, and there was no increase in serum or salivary glucose when compared to the group of trainees.

Atropine is a natural tropane alkaloid extracted from belladonna (*Atropa belladonna*), zabumba (), mandrake (*Mandragora officinarum*), and other plants of the Solanaceae family, including potato (), tomato (*Solanum lycopersicum*), bell pepper, and chili pepper (*Capsicum*), as well as other species of great importance for human nutrition.^55^ It has a plasma half-life of approximately 2 h and is excreted unchanged in urine.^56^ The fact that this metabolite has increased only in instructors may be related to age and individual food choices. Considering that pilots eat in the same ranch when they are at the air base, there are still individual food preferences that we were unable to quantify. In addition, instructors’ urine displayed significant correlations between key blood parameters and atropine and trigonelline, possibly similar to food intake of these compounds in instructors. It is worth noting that correlation is not causality.

The metabolic proportions of the serum phenylalanine:tyrosine ratio did not show changes, but the glutamate:glutamine ratio showed a significant value only in trainees, suggesting a possible abnormal mitochondrial functioning in the trainee’s group; however, additional studies need to be carried out to confirm this hypothesis.

This study emphasizes the need for long-term monitoring of pilots’ metabolic health to develop interventions that optimize performance, reduce fatigue, and minimize accident risk. Limitations included a lack of control over diet, water intake, and flight types. Nevertheless, the collection of biofluid samples from A-29 pilots represents a significant step in clinical aviation research in Brazil. Further research could enhance our understanding of metabolic changes and inform pilot health strategies.

## Conclusion

Flying A-29 fighter jets induces acute metabolic changes in pilots, significantly affecting lactate levels, certain amino acids such as glutamine, and immune defense cells. Serum and saliva were identified as the most effective biofluids for detecting these metabolic changes. Although blood collection for routine monitoring of pilots is complex, saliva offers a promising alternative due to its ease of collection and handling, making it suitable for regular monitoring. However, further research is needed to better understand the causes of these acute metabolic changes and their long-term effects. We recommend the personalized monitoring of pilots from the onset of their training and the establishment of a biofluid biobank. This approach would facilitate interventions to improve pilots’ adaptation to flying and minimize the long-term health risks faced by this population.

## Supplementary Material



## Notes

Additional experimental details. Figure S1: Representative graph of circulating endothelial cells (CECs) before flight of trainee and instructor pilots. Table S1: Complete data of hemogram, lipidogram, coagulogram, and CECs of trainee and instructor pilots before and after flights. Figure S2: Representative image of saliva 1D ^1^H NMR spectrum. Figure S3: Representative image of urine 1D ^1^H NMR spectrum. Figure S4: ^1^H NMR spectra overlay serum. Figure S5: ^1^H NMR spectra overlay saliva. Figure S6: ^1^H NMR spectra overlay urine. Table S2: Table of chemical shifts used for relative quantification of serum metabolites from instructor and trainee pilots. Table S3: Table of chemical shifts used for relative quantification of saliva metabolites from instructor and trainee pilots. Table S4: Table of chemical shifts used for relative quantification of urine metabolites from instructor and trainee pilots. Table S5: Peak report from HSQC submitted to COLMARm. Table S6: Categorical variables of pilots. Figure S7: Metabolic ratios of serum. Figure S8: Correlations between serum metabolites and key blood parameters of trainee and instructor pilots. Figure S9: Correlations between saliva metabolites and key blood parameters of trainee and instructor pilots. Figure S10: Correlations between urine metabolites and key blood parameters of trainee and instructor pilots (PDF)

## Notes

The NMR-based metabolomics data have been deposited in the MetaboLights repository (https://www.ebi.ac.uk/metabolights/editor/study/REQ20250222208808) with the data set identifier REQ20250222208808.
