# Supplementary material for: Acute Metabolic Effects in Brazilian A‑29 Fighter Pilots by NMR-Based Metabolomics
Source: J Proteome Res. 2025 Jul 21;24(8):4033–43. doi: 10.1021/acs.jproteome.5c00129 (PMC12322951; doi:10.1021/acs.jproteome.5c00129)
Supplement: Supplementary file 1 [file pr5c00129_si_001.pdf]

Supplementary Data

Acute metabolic effects in Brazilian A-29 fighter pilots by NMR-based metabolomics

Roberta Verissimo França de Oliveira[1], Grace Barros de Sá[2], Alanny Cristine dos Santos Pinheiro[1], Antonia Claudia Jácome da Câmara[6], Palmielly Diógenes[3], Adriano Percival Calderaro Calvo[2,§,&], Laila Ribeiro Fernandes[4], Luísa Soares da Silva[4], Rafael Loureiro Simões[5], Verônica Morandi[4], Gilson Costa dos Santos Junior[1*], Paulo Farinatti[2*]

1. Laboratory of Metabolomics (LabMet), State University of Rio de Janeiro (UERJ), IBRAG/Department of Genetics, Rio de Janeiro, RJ, Brazil.

2. Laboratory of Physical Activity and Healthy Promotion (Labsau), State University of Rio de Janeiro (UERJ), Institute of Physical Education and Sports, Rio de Janeiro, RJ, Brazil.

3. Integrated Laboratory of Clinical Analysis (LIAC), Federal University of Rio Grande do Norte (UFRN), Natal, RN, Brazil.

4. Laboratory of Endothelial Cell Biology & Angiogenesis/ Department of Cell Biology, University of Rio de Janeiro (UERJ), IBRAG/Department of Cell Biology, Rio de Janeiro RJ, Brazil.

5. Laboratory of Cellular and Molecular Pharmacology/ Department of Cell Biology, University of Rio de Janeiro (UERJ), IBRAG/Department of Cell Biology, Rio de Janeiro RJ, Brazil.

6. Laboratório de Biologia de Parasitos e de Doença de Chagas, Departamento de Análises Clínicas e Toxicológicas, CCS, UFRN.

§. Brazilian Air Force University (UNIFA), Military Human Performance Post-Graduation, Rio de Janeiro, RJ, Brazil.

&. Institute of Aerospace Medicine Brigadier Doctor Roberto Teixeira (IMAE), Rio de Janeiro, RJ, Brazil

*** Corresponding Authors**

Paulo Farinatti. E-mail: paulo.farinatti@uerj.br.

Gilson Costa Santos Jr. E-mail: gilson.junior@uerj.br.

Summary

Figure S1 .... Pg. S5

Table S1 .... Pg. S6-S7

Figure S2 .... Pg. S8

Figure S3 .... Pg. S8

Figure S4 .... Pg. S9

Figure S5 .... Pg. S10

Figure S6 .... Pg. S10

Table S2 .... Pg. S11-S19

Table S3 .... Pg. S20-S33

Table S4 .... Pg. S34-S42

Table S5 .... Pg. S43-S44

Table S6 .... Pg. S45

Figure S7 .... Pg. S45

Figure S8 .... Pg. S46

Figure S9 .... Pg. S46

Figure S10 .... Pg. S47

Blood Count

For the CBC, 4 ml of whole blood in an EDTA tube was analyzed on the ABX Micros 60 (HORIBA) equipment, using the Ebralyse-MI, EBRAM reagents Ebraterg, EBRAM. Ebraton; EBRAM. D-check D (low, normal, high) – ABX Micros 60, DIAGNO and Wright dye, BIOCLIN. Initially, all hematological settings were reset to ensure that there were no contaminants, then analyzes of low, normal, high controls were carried out and, when necessary, calibrations were made of the settings that were outside the desirable values, thus the samples were analyzed in the equipment and finally blood smears stained according to Wright were made. Number of Red Blood Cells, Leukocytes, Platelets: counted from the change in electrical impedance through the passage of cells through calibrated micro-openings of 80 and 50 micrometers. Hemoglobin: measurement of the chromogenic product cyanmethemoglobin by spectrophotometry at 550 nm. Hematocrit: obtained from MCV values and number of red blood cells. VCM: obtained by passing red blood cells through micro-openings calibrated over time. The generated electronic pulses are grouped by height and an overall medium is determined. HCM: obtained from hemoglobin values and number of red blood cells. MCHC: obtained from hemoglobin and hematocrit values. RDW: distribution amplitude of the pulses generated by the passage of red blood cells through the calibrated micro-openings. VPM: obtained by passing the plates through micro-openings calibrated over time. The generated electronic pulses are grouped by height and an overall medium is determined. Leukocyte differential and morphology: specific on stained slide, according to Wright, under special optical microscopy at 1000x magnification.

Biochemistry

For the biochemistry tests, 8 ml of serum in a tube with gel and clot activator was used, the Labmax Plenno, LABTEST equipment and the reagents: Glucose PAP Liquiform, LABTEST. CK-NAC Liquiform-117, LABTEST. Liquiform-76 cholesterol, LABTEST. HDL-145, latest. Liquiform-87 triglycerides, LABTEST. Qualitrol 1H/2H, LABTEST. Initially, the “blanks” of the reagents were checked, the experimental control and the concentration of the reagents that were outside the desirable values were carried out. Finally, the samples were

analyzed, and the values of each analyte were recorded. Creatine kinase: determined by kinetic evidence using the Szasz method adapted by the IFCC with photometric reading at 340nm. Total cholesterol: determined by endpoint occurrence using the TRINDER method with photometric reading at 505nm. HDL cholesterol: determined by endpoint evidence using the selective surfactant method with photometric reading at 600nm LDL, VLDL, non-HDL cholesterol: obtained from the values of total cholesterol, HDL and triglycerides according to the proposal proposed by Martin et al published in the Journal of the American Medical Association and recently by the national dyslipidemia guideline. Triglycerides: determined by the occurrence of an end point using the TRINDER method with photometric reading at 505nm.

Coagulogram

Blood was obtained through venipuncture using sodium citrate tubes (Biocon™, Belo Horizonte, Minas Gerais, Brazil). The collected samples underwent centrifugation (CLOTimer, CLOT Bios Diagnostica™, Sorocaba, São Paulo, Brazil) at 1500g for 15 min at room temperature. Prior to analysis, the samples were preheated to 37°C. The analysis was conducted using a photo-optical system. Various reagents were employed: Liquid PTT (Partial Thrombin Time, GOLD ANALISA™, Belo Horizonte, Minas Gerais, Brazil); aPTT (activated Partial Thrombin Time, CLOT Bios Diagnostica™, Sorocaba, São Paulo, Brazil); Fibrinogen K136 (BIOCLIN™, Belo Horizonte, Minas Gerais, Brazil). To assess the prothrombin activity time, tissue factor thromboplastin plus calcium were added to each sample. The International Sensitivity Index (ISI) of the reagent used was 1.04. For PTT, ellagic acid and phospholipids were added, followed by calcium. In the case of fibrinogen, samples were diluted 1:10 and preheated to 37°C after the addition of thrombin.

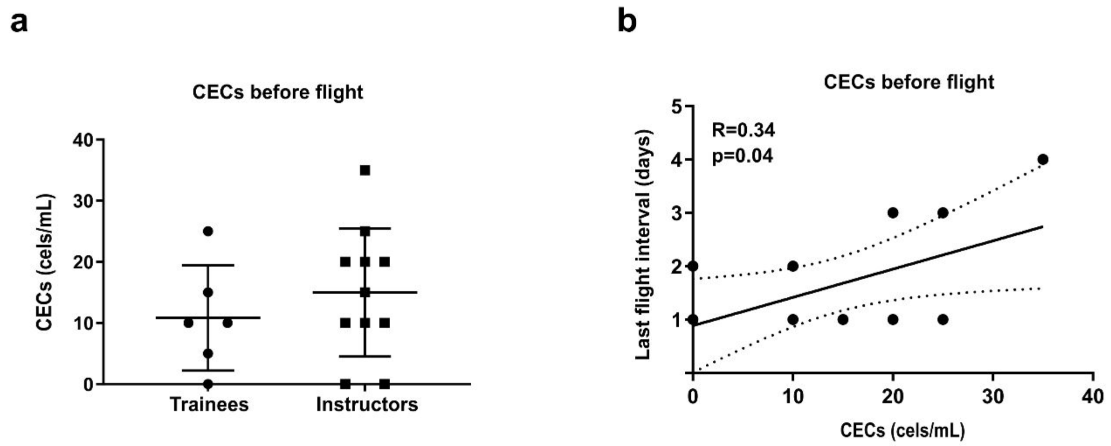

Figure S1. Representative graph Trainee and Instructors of Cell endothelial (CECs) before flight. Analysis carried out with Mann Whitney, test nonparametric data express for mean values and data dispersion details.

Table S1. Trainee and instructor pilots had an increase in immune cells after the flight. Data hemogram, lipidogram, coagulogram, and endothelial cells trainee and instructor pilots before and after the flight. Paired analysis by Wilcoxon nonparametric test after normality Shapiro-Wilk test, data expressed as median, minimum and maximum. Multiple tests with single pooled variance with a desired false discovery rate of 0.05.

		Trainees (n=12)										Instructors (n=20)									
**Class**	**Reference interval**	**Before**	**max**	**mim**	**After**	**max**	**mim**	**#**	**&**	**P value**	**q value**	**Before**	**max**	**mim**	**After**	**max**	**mim**	**#**	**&**	**P value**	**q value**
**Red Cells ($x10^6/mm^3$)**	4.5 - 5.9	5.155	5.58	4.18	5.09	5.67	4.26	99%	-1.3%	0.20	0.71	5.11	5.73	4.33	5.01	5.54	4.29	98%	-2.0%	0.19	0.68
**Hemoglobin (g/dL)**	14 -18	15.25	16.9	14.1	15.3	17.1	14.1	100%	0.3%	0.22	0.71	15.65	16.4	13.4	15.35	16.6	14	98%	-1.9%	0.18	0.68
**Hematocrit (%)**	39 - 54	45.1	49	38.1	44	48.8	39.8	98%	-2.4%	0.15	0.64	45.05	47.3	40.3	44.75	47	40	99%	-0.7%	0.55	0.97
**Mean Corpuscular Volume (fL)**	83 - 98	88	93	81.7	87.8	93	81.5	100%	-0.2%	**0.03***	0.3	89	93	79.6	89	94	78.9	100%	0.0%	0.55	0.97
**Mean Corpuscular Hemoglobin (pg)**	28 - 32	30.75	289	27.2	30.55	33.2	27.3	99%	-0.7%	0.72	>0,99	30.95	33.7	26.9	30.85	32.8	27.3	100%	-0.3%	0.49	0.97
**MCH (g/dL)**	31 - 35	34.55	36	33.3	34.95	35.5	33	101%	1.2%	0.50	0.93	34.9	341	32.9	34.95	35.7	32.1	100%	0.1%	0.72	0.97
**RDW (%)**	11.5% - 14.5%	12.5	12.9	11.8	12.45	13	12.1	100%	-0.4%	0.88	>0,99	12.7	13.3	12.3	12.8	13	12.2	101%	0.8%	0.84	>0,99
**Number Platelets (mm^3)**	150.000 - 450.00	212000	306000	165000	223000	335000	162000	105%	5.2%	0.58	0.97	250000	412000	112000	257000	433000	119000	103%	2.8%	0.59	0.97
**Average Platelet Volume (fL)**	7.5 -11.5	11.1	11.3	10.7	10.85	11.2	10.7	98%	-2.3%	0.13	0.63	11.1	11.5	10.3	11	11.3	10	99%	-0.9%	0.18	0.68
**Number Leukocytes (mm^3)**	4.000 - 10.000	6700	9200	4400	7600	9600	1580	113%	13.4%	0.07	0.42	6650	9700	4300	6850	8500	1190	103%	3.0%	0.07	0.51
**Rods Cell (%)**	3.0 - 5	0	2	0	0	1	0	-	-	>0,999	>0,99	0	0	0	0	1	0	-	-	0.06	0.51
**Rods Cell (mm^3)**	120 - 500	0	140	0	0	96	0	-	-	>0,999	>0,99	0	0	0	0	85	0	-	-	0.06	0.51
**Segmented (%)**	58 - 66	60	72	42	67	87	58	112%	11.7%	**0.01***	0.13	54.5	71	34	53	86	33	97%	-2.8%	0.21	0.68
**Segmented (mm^3)**	2320 - 6600	3801.5	5888	2184	5135	13746	2842	135%	35.1%	**0.003***	0.13	3492.5	4964	1677	3318.5	7138	1739	95%	-5.0%	0.65	0.97
**Eosinophils (%)**	2.0 - 4.0	2.5	8	1	2	7	0	80%	-20.0%	0.13	0.63	2	10	1	2	7	0	100%	0.0%	0.35	0.95
**Eosinophils (mm^3)**	80 - 400	180	448	74	169	532	0	94%	-6.1%	0.85	>0,99	146.5	660	68	150	518	0	102%	2.4%	0.6	0.97
**Basophils (%)**	0 - 1	0	1	0	0	0	0	-	-	>0,999	>0,99	0	1	0	0	1	0	-	-	>0,99	>0,99
**Basophils (mm^3)**	0 -100	0	44	0	0	0	0	-	-	>0,999	>0,99	0	73	0	0	85	0	-	-	>0,99	>0,99
**Lymphocytes (%)**	21 - 35	32	47	16	25	32	7	78%	-21.9%	**0.01***	0.16	35	59	23	35.5	53	10	101%	1.4%	0.22	0.68
**Lymphocytes (mm^3)**	840 -3500	2251	2961	704	1610	2464	990	72%	-28.5%	0.27	0.72	2157	5723	1081	2483	6307	830	115%	15.1%	**0.007***	0.28
**Monocytes (%)**	4 - 8	6	9	3	5.5	9	1	92%	-8.3%	0.23	0.71	6	12	2	7	11	2	117%	16.7%	0.86	>0,99
**Monocytes (mm^3)**	160 - 800	415	621	222	450.5	632	90	109%	8.6%	>0,999	>0,99	429	972	138	450	810	106	105%	4.9%	0.23	0.68
**Blood glucose (mg/dL)**	<100	79.15	111	62	84	102	71	106%	6.1%	0.06	0.42	81.7	95.8	64	82.6	110	53	101%	1.1%	0.92	>0,99

**Total cholesterol (mg/dL)**	< 100	152.5	235.8	85	156.85	243.5	104	103%	2.9%	0.39	0.85	181.45	311.9	147	196.4	320.6	151	108%	8.2%	0.69	0.97
**Cholesterol HDL (mg/dL)**	> 40	51.5	74	25	51.5	76	27	100%	0.0%	0.24	0.71	52	68	37.4	56.2	71	38.6	108%	8.1%	0.72	0.97
**Cholesterol LDL (mg/dL)**	100 -129	86	182.6	46	86	187.6	21	100%	0.0%	0.64	0.97	110.7	217.4	78	118.85	229.5	80	107%	7.4%	0.56	0.97
**Cholesterol VLDL (mg/dL)**	< 30	14	27.9	9	15.5	28.5	7	111%	10.7%	0.62	0.97	19.5	40	11.7	19.15	35	9.6	98%	-1.8%	0.49	0.97
**Cholesterol NÃO-HDL (mg/dL)**	< 130	100	138	60	101.5	167	28	102%	1.5%	0.41	0.85	128	212	94	127.5	204	94	100%	-0.4%	0.68	0.97
**Triglycerides (mg/dL)**	<150	64.5	139.6	30	72.4	142.7	26	112%	12.2%	0.96	>0,99	92	247	48	94	187	42	102%	2.2%	0.38	0.97
**Creatine kinase (U/L)**	26 –140	219.5	607	72	211.5	640	67	96%	-3.6%	0.49	0.93	179	737	75	180.5	749	82	101%	0.8%	>0,99	>0,99
**PTT (s)**	25 - 35	16.05	34	12.1	14.8	43.7	12	92%	-7.8%	0.73	>0,99	14.35	25	12	14.8	22.5	12.5	103%	3.1%	0.83	>0,99
**PPT (INR)**	1.00 -1.08	1.165	2.62	1	1.065	3.4	1	91%	-8.6%	0.64	0.97	1.025	1.83	1	1.06	1.7	1	103%	3.4%	0.95	>0,99
**PPT (%)**	70 -100	64.7	100	16.1	84	100	11.5	130%	29.8%	0.84	>0,99	91.8	100	25.8	82.85	100	30.4	90%	-9.7%	0.72	0.97
**aPPT (s)**	26.1- 45.9	33.1	46.9	26.2	31.75	51.9	26.2	96%	-4.1%	0.38	0.85	29.5	38.6	25.4	31.75	44	24.5	108%	7.6%	0.07	0.51
**aPPT (p/c)**	0.90 -1.2	1.105	1.56	0.87	1.06	1.73	0.87	96%	-4.1%	0.38	0.85	0.985	1.29	0.85	1.06	1.47	0.82	108%	7.6%	0.08	0.51
**Fibrinogen (mg/dL)**	200 - 400	220.5	308	70	211	359	75	96%	-4.3%	0.52	0.93	209	387	95	230.5	399	101	110%	10.3%	0.19	0.68

# corresponds to Instructors over trainees.
& Delta
*Values less than $p < 0.05$.
** reference value used by the laboratory
MCH - Mean Corpuscular Hemoglobin; RDW - Red Blood Cell Distribution Width; PTT- Partial Thromboplastin Time; aPTT - Activated Partial Thromboplastin Time.

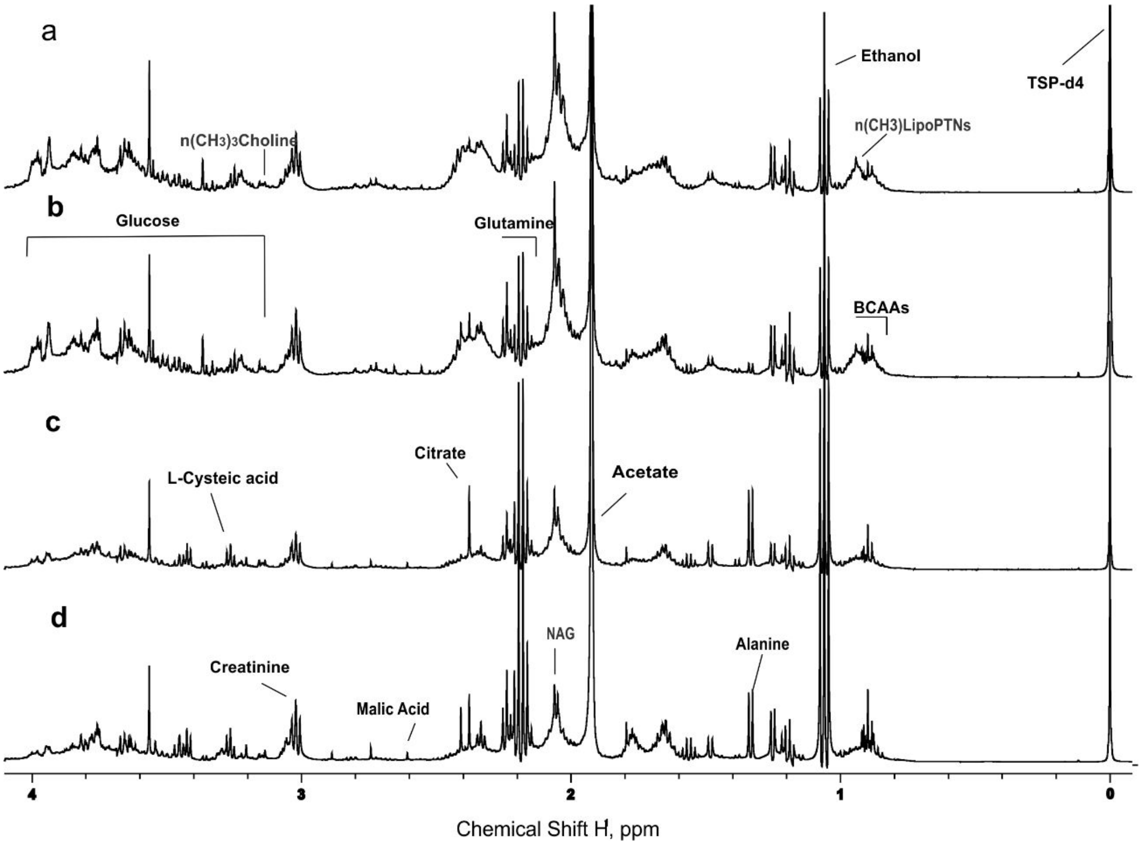

Figure S2. Representative image of saliva 1D ^{1}H NMR spectrum. (a) instructor before flight; **(b)** instructor after flight; **(c)** trainee before flight; **(d)** trainee after flight.

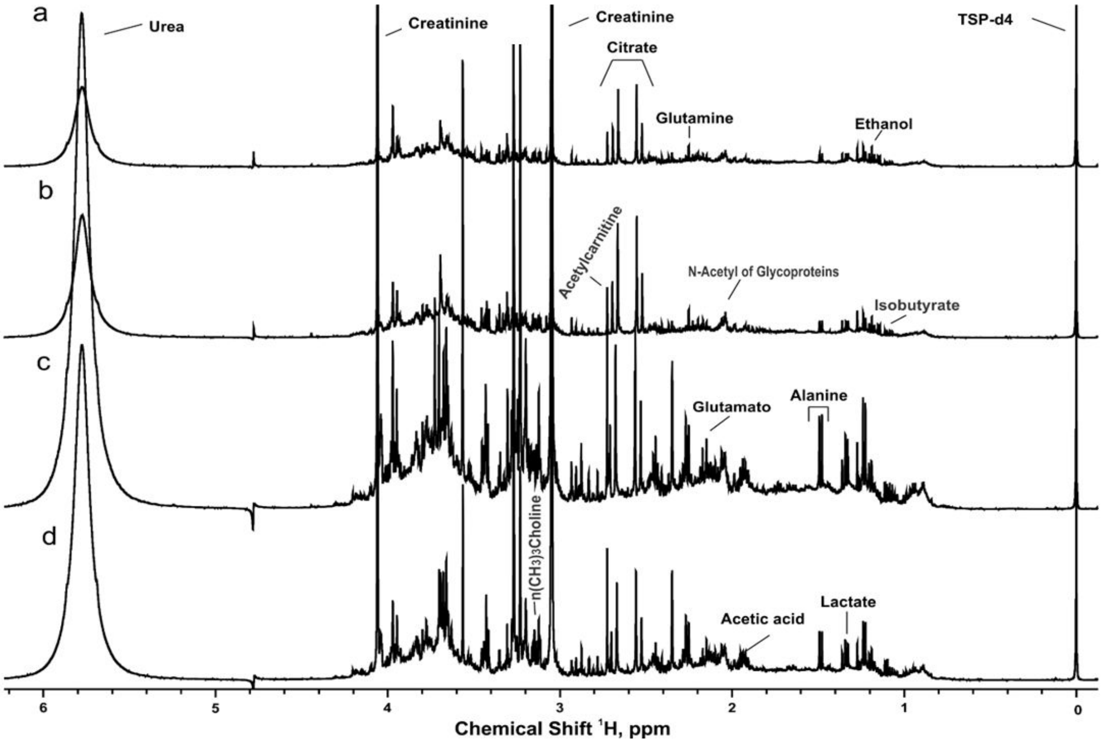

Figure S3. Representative image of urine 1D ^{1}H NMR spectrum. (a) instructor before flight; **(b)** instructor after flight; **(c)** trainee before flight; **(d)** trainee after flight.

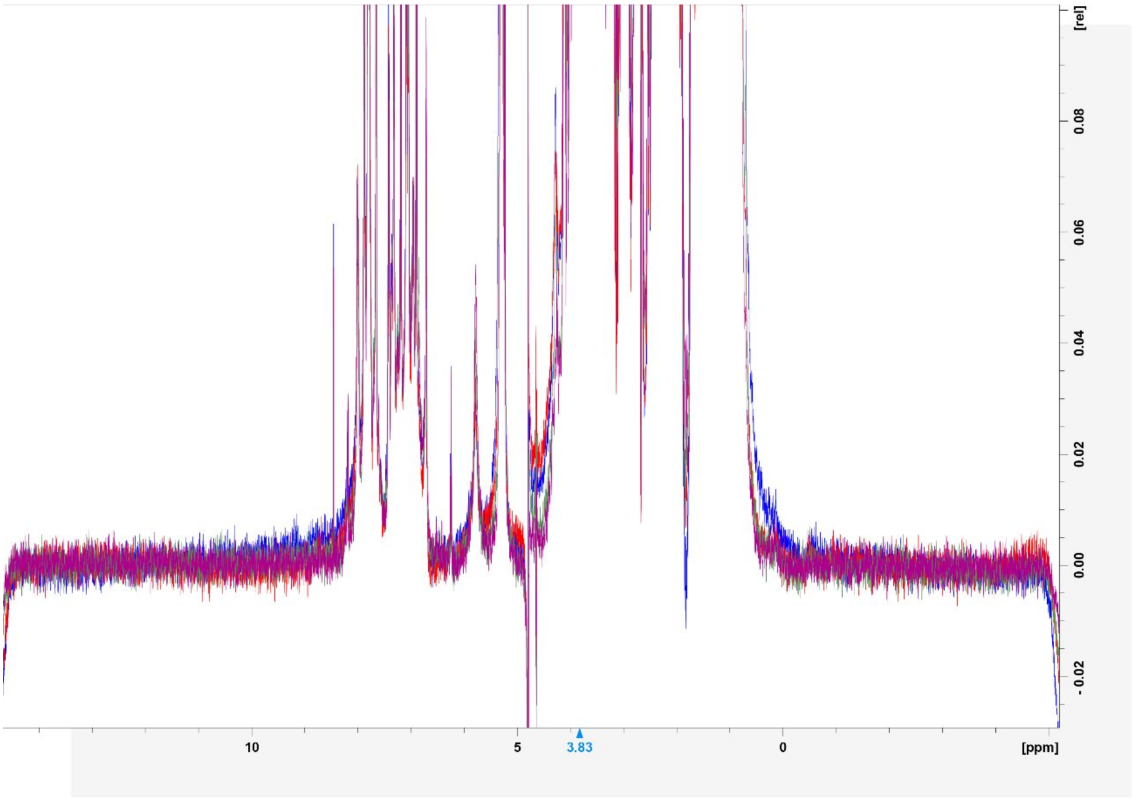

Figure S4. ^{1}H NMR Spectra overlay serum

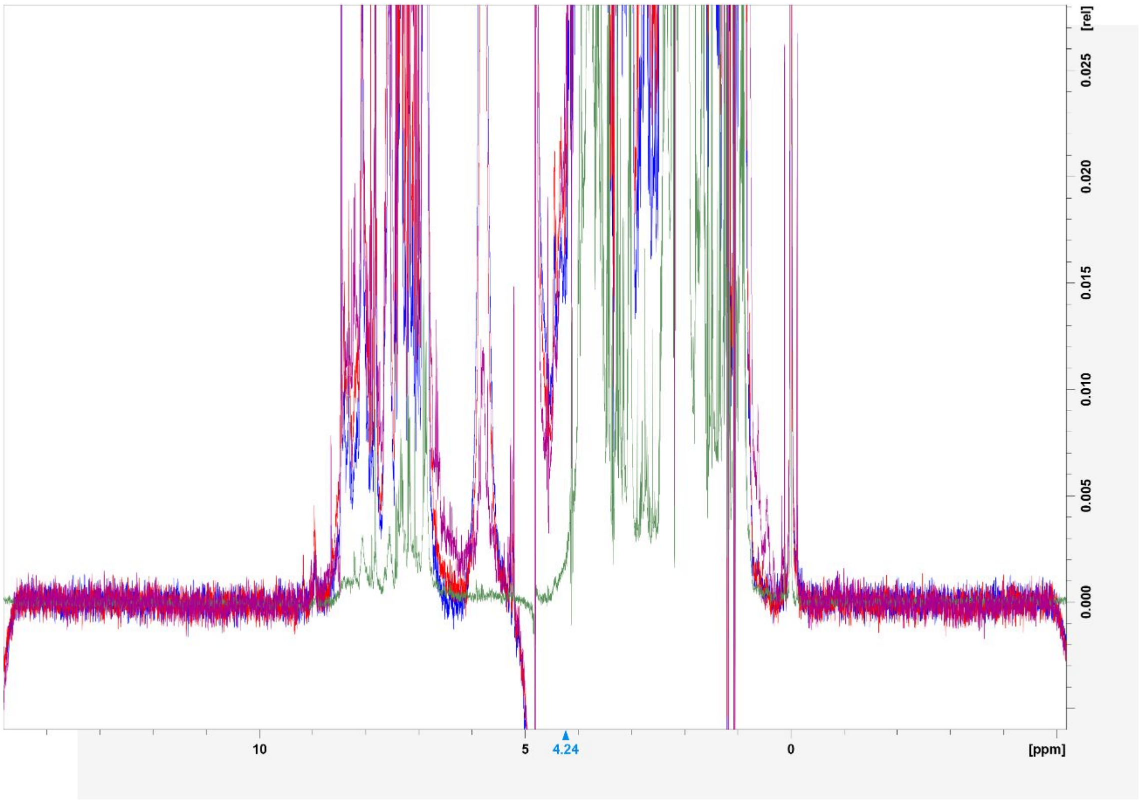

Figure S5. ^{1}H NMR Spectra overlay saliva

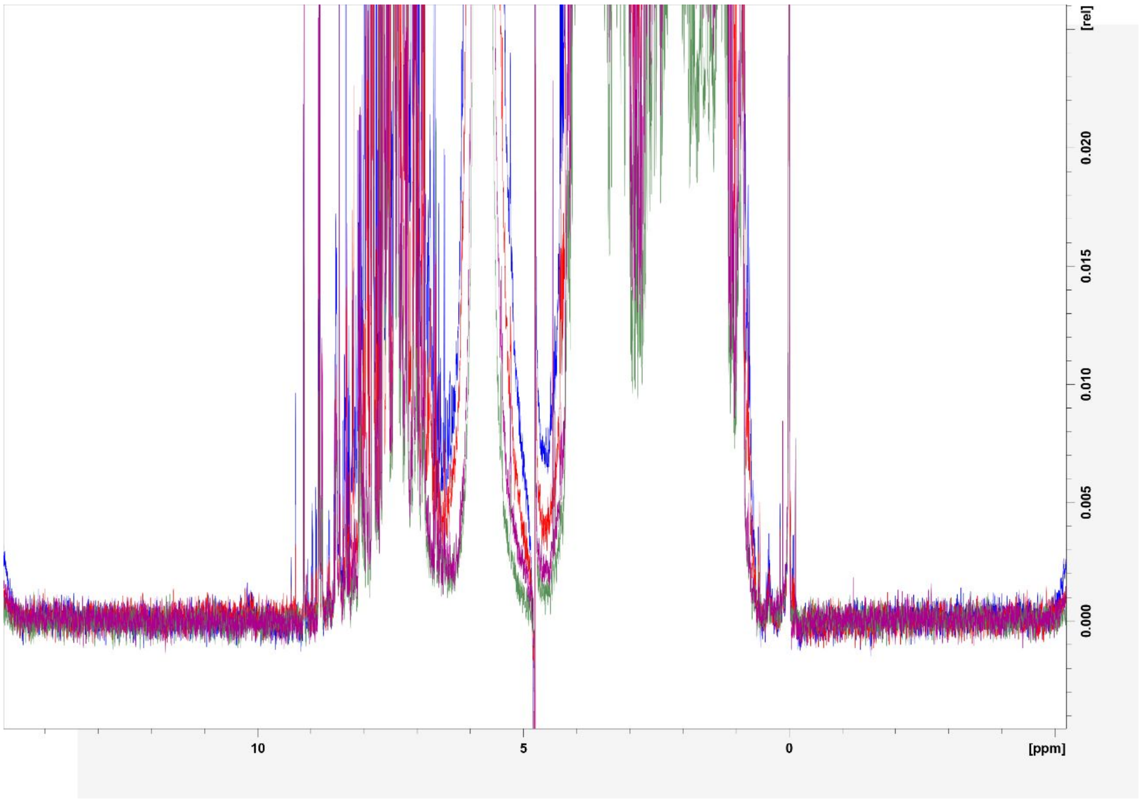

Figure S6. ^{1}H NMR Spectra overlay urine

Table.S2 Table of chemical shifts used for relative quantification of serum metabolites from Instructors and Trainees. Paired analysis, carried out with the Wilcoxon test nonparametric, data expressed as median, minimum and maximum after Shapiro-Wilk normality test. # corresponds to instructors over trainees. & corresponds to the % from #. *Values less than $p < 0.05$.

Instructors											
Chemical Shift (1H. ppm)	Metabolite	Before	max	mim	After	max	mim	P value	q value	#	&
1.32*, 4.10 - 4.12	Lactate	0.04803	0.05972	0.03627	0.04211	0.05150	0.03569	0.003	0.11	88%	-12%
3.22* - 3.20	$n(CH_3)_3$ Choline	0.03252	0.04963	0.02536	0.03634	0.04974	0.02476	0.003	0.11	112%	12%
3.36*		0.00376	0.00603	0.00250	0.00436	0.00657	0.00282	0.003	0.11	116%	16%
8.18*		0.00010	0.00012	0.00003	0.00004	0.00015	0.00000	0.003	0.11	40%	-60%
8.20*		0.00010	0.00012	0.00003	0.00005	0.00016	0.00001	0.003	0.11	48%	-52%
8.46*	Formate	0.00008	0.00012	0.00005	0.00011	0.00016	0.00004	0.003	0.11	134%	34%
3.6*		0.00435	0.00561	0.00346	0.00474	0.00597	0.00375	0.005	0.12	109%	9%
0.84*-0.82	$n(CH_3)$Lipo PTNs	0.02127	0.02764	0.01692	0.02367	0.02945	0.01841	0.006	0.12	111%	11%
2.32* - 2.34	Glutamate	0.00194	0.00255	0.00146	0.00223	0.00291	0.00173	0.006	0.12	115%	15%
2.38*		0.00127	0.00188	0.00091	0.00146	0.00210	0.00111	0.006	0.12	115%	15%
2.12*		0.00441	0.00571	0.00340	0.00455	0.00577	0.00372	0.007	0.12	103%	3%
2.40*		0.00131	0.00201	0.00092	0.00151	0.00217	0.00111	0.008	0.12	115%	15%
3.62*		0.00405	0.00513	0.00318	0.00411	0.00519	0.00331	0.009	0.13	102%	2%
1.34*		0.03099	0.03716	0.02167	0.02696	0.03297	0.02205	0.01	0.14	87%	-13%
2.08*		0.00793	0.00908	0.00672	0.00822	0.01004	0.00707	0.01	0.14	104%	4%
2.10*		0.00428	0.00494	0.00351	0.00437	0.00513	0.00362	0.01	0.14	102%	2%
2.30*		0.00200	0.00238	0.00152	0.00206	0.00271	0.00171	0.01	0.14	103%	3%
2.42*		0.00190	0.00265	0.00128	0.00203	0.00265	0.00155	0.01	0.14	107%	7%
3.58*, 4.26, 1.34	Threonine	0.00529	0.00698	0.00411	0.00554	0.00728	0.00432	0.01	0.14	105%	5%
1.2* - 1.28	$n(CH_2)$Lipo PTNs	0.00809	0.00938	0.00765	0.00848	0.01136	0.00767	0.02	0.15	105%	5%
1.56*		0.00301	0.00397	0.00032	0.00270	0.00384	0.00018	0.02	0.16	90%	-10%
1.58*		0.00304	0.00431	0.00019	0.00256	0.00409	0.00010	0.02	0.16	84%	-16%
1.92*		0.00146	0.00224	0.00095	0.00155	0.00220	0.00115	0.02	0.15	106%	6%
2.04*		0.01970	0.02252	0.01668	0.02067	0.02487	0.01730	0.02	0.16	105%	5%
2.28*		0.00230	0.00300	0.00175	0.00251	0.00423	0.00198	0.02	0.15	109%	9%
3.66*		0.00678	0.00880	0.00569	0.00736	0.00992	0.00604	0.02	0.15	109%	9%
3.68*		0.00678	0.00801	0.00523	0.00717	0.00872	0.00530	0.02	0.15	106%	6%
3.94*		0.00200	0.00263	0.00135	0.00234	0.00274	0.00165	0.02	0.16	117%	17%
5.30*		0.00177	0.00265	0.00064	0.00170	0.00249	0.00048	0.02	0.16	96%	-4%
1.30*		0.04739	0.06627	0.02215	0.03901	0.06506	0.02122	0.03	0.17	82%	-18%
2.44* - 2.48, 2.10	Glutamine	0.00289	0.00417	0.00201	0.00304	0.00379	0.00239	0.03	0.16	105%	5%
2.56*		0.00062	0.00087	0.00025	0.00069	0.00086	0.00026	0.03	0.16	112%	12%
5.32*		0.00161	0.00223	0.00022	0.00138	0.00243	0.00018	0.03	0.17	85%	-15%
1.22*		0.01498	0.01745	0.01361	0.01584	0.01820	0.01419	0.04	0.2	106%	6%
1.38*		0.00442	0.00680	0.00132	0.00371	0.00646	0.00149	0.04	0.2	84%	-16%
1.40*		0.00328	0.00460	0.00150	0.00311	0.00449	0.00144	0.04	0.2	95%	-5%
1.42*, 1.46 - 1.48	Alanine	0.00266	0.00332	0.00113	0.00248	0.00327	0.00097	0.04	0.2	93%	-7%
2.24*		0.00328	0.00435	0.00142	0.00289	0.00447	0.00157	0.04	0.2	88%	-12%
3.20*		0.01719	0.02179	0.01414	0.01790	0.02454	0.01352	0.04	0.2	104%	4%

**3.24***		**0.01883**	**0.02625**	**0.01341**	**0.02049**	**0.02664**	**0.01598**	**0.04**	**0.2**	**109%**	**9%**
**3.64***		**0.00507**	**0.00667**	**0.00401**	**0.00533**	**0.00756**	**0.00434**	**0.04**	**0.2**	**105%**	**5%**
1.36		0.00865	0.01277	0.00289	0.00719	0.01230	0.00323	0.05	0.21	83%	-17%
1.54		0.00236	0.00284	0.00045	0.00233	0.00271	0.00030	0.05	0.21	99%	-1%
3.56		0.00846	0.01106	0.00629	0.00871	0.01188	0.00686	0.05	0.21	103%	3%
3.80		0.00421	0.00520	0.00293	0.00465	0.00557	0.00330	0.05	0.21	110%	10%
3.90		0.00738	0.00948	0.00526	0.00813	0.01012	0.00599	0.05	0.21	110%	10%
3.96		0.00145	0.00199	0.00097	0.00169	0.00201	0.00113	0.05	0.21	116%	16%
7.88		0.00063	0.00088	0.00036	0.00073	0.00094	0.00020	0.05	0.21	116%	16%
2.16		0.00231	0.00304	0.00181	0.00240	0.00282	0.00186	0.06	0.23	104%	4%
2.76		0.00230	0.00310	0.00150	0.00231	0.00346	0.00078	0.06	0.23	100%	0%
5.78		0.00026	0.00063	0.00008	0.00033	0.00076	0.00007	0.06	0.22	126%	26%
0.80		0.00316	0.00378	0.00219	0.00339	0.00415	0.00272	0.07	0.23	107%	7%
**0.88 - 0.86**	n(CH_3) LipoPTNs	0.02945	0.04202	0.02110	0.02804	0.04525	0.02035	0.07	0.23	95%	-5%
0.90		0.01938	0.02788	0.00916	0.01520	0.02889	0.00914	0.07	0.24	78%	-22%
2.22		0.00294	0.00335	0.00246	0.00319	0.00492	0.00233	0.07	0.24	109%	9%
3.70		0.01216	0.01579	0.00885	0.01281	0.01683	0.00894	0.07	0.24	105%	5%
3.86		0.00711	0.00935	0.00522	0.00760	0.00925	0.00532	0.07	0.23	107%	7%
**3.98**	Phosphoet hanolamine	0.00143	0.00180	0.00091	0.00156	0.00188	0.00100	0.07	0.23	109%	9%
4.02		0.00062	0.00129	0.00027	0.00072	0.00113	0.00046	0.07	0.24	117%	17%
5.80		0.00019	0.00041	0.00006	0.00024	0.00051	0.00006	0.07	0.24	128%	28%
2.26		0.00266	0.00323	0.00133	0.00242	0.00327	0.00140	0.08	0.24	91%	-9%
3.76		0.00876	0.01145	0.00689	0.00956	0.01282	0.00733	0.08	0.25	109%	9%
7.66		0.00057	0.00087	0.00024	0.00070	0.00109	0.00023	0.08	0.25	123%	23%
0.92		0.00657	0.00955	0.00438	0.00612	0.00870	0.00411	0.09	0.26	93%	-7%
2.46		0.00247	0.00362	0.00164	0.00257	0.00333	0.00199	0.09	0.26	104%	4%
3.52		0.00646	0.00826	0.00452	0.00699	0.00853	0.00500	0.09	0.26	108%	8%
3.92		0.00460	0.00574	0.00282	0.00474	0.00676	0.00316	0.09	0.26	103%	3%
7.64		0.00054	0.00090	0.00017	0.00042	0.00093	0.00014	0.09	0.26	78%	-22%
2.02		0.01011	0.01114	0.00904	0.01011	0.01132	0.00885	0.1	0.26	100%	0%
2.36		0.00212	0.00259	0.00172	0.00235	0.00265	0.00171	0.1	0.26	111%	11%
3.26		0.01327	0.01885	0.01048	0.01377	0.01735	0.01098	0.1	0.26	104%	4%
3.54		0.00933	0.01161	0.00658	0.00994	0.01254	0.00729	0.1	0.26	106%	6%
3.74		0.01271	0.01540	0.00848	0.01377	0.01734	0.00925	0.1	0.26	108%	8%
3.88		0.01366	0.01745	0.00963	0.01453	0.01833	0.01104	0.1	0.27	106%	6%
2.80		0.00140	0.00176	0.00080	0.00137	0.00192	0.00008	0.11	0.28	98%	-2%
3.78		0.00984	0.01209	0.00677	0.01001	0.01328	0.00686	0.11	0.28	102%	2%
4.14		0.00038	0.00089	0.00019	0.00040	0.00088	0.00020	0.11	0.28	105%	5%
7.78		0.00103	0.00126	0.00064	0.00112	0.00150	0.00064	0.12	0.29	109%	9%
7.82		0.00068	0.00098	0.00042	0.00079	0.00117	0.00028	0.12	0.29	117%	17%
0.86		0.02724	0.03478	0.02214	0.02791	0.03231	0.02397	0.13	0.31	102%	2%
3.84		0.00937	0.01168	0.00626	0.01015	0.01321	0.00654	0.13	0.31	108%	8%
4.00		0.00143	0.00218	0.00089	0.00161	0.00202	0.00098	0.13	0.31	113%	13%
1.46		0.00485	0.00702	0.00351	0.00484	0.00578	0.00357	0.14	0.31	100%	0%
2.14		0.00423	0.00591	0.00324	0.00439	0.00573	0.00342	0.14	0.31	104%	4%
3.04		0.00429	0.00600	0.00312	0.00409	0.00578	0.00339	0.14	0.31	95%	-5%

3.06		0.00145	0.00174	0.00099	0.00151	0.00172	0.00065	0.14	0.31	104%	4%
3.30		0.00181	0.00252	0.00127	0.00199	0.00220	0.00133	0.14	0.31	110%	10%
3.72		0.01585	0.02085	0.01176	0.01687	0.02270	0.01303	0.14	0.31	106%	6%
4.04		0.00055	0.00101	0.00019	0.00055	0.00093	0.00035	0.14	0.31	100%	0%
3.48		0.01318	0.01756	0.00927	0.01373	0.01847	0.00892	0.15	0.33	104%	4%
5.76		0.00021	0.00046	0.00007	0.00023	0.00053	0.00006	0.15	0.33	113%	13%
8.22		0.00002	0.00006	0.00001	0.00002	0.00005	0.00000	0.15	0.33	86%	-14%
**2.5, 2.53–2.54, 2.65, 2.68**	Citrate	0.00078	0.00107	0.00031	0.00083	0.00104	0.00041	0.16	0.33	107%	7%
2.52		0.00097	0.00139	0.00047	0.00104	0.00139	0.00072	0.16	0.33	108%	8%
1.24		0.02391	0.02813	0.02040	0.02444	0.02925	0.02114	0.17	0.33	102%	2%
3.44		0.00769	0.01000	0.00522	0.00877	0.01065	0.00516	0.17	0.33	114%	14%
3.46		0.01317	0.01822	0.00945	0.01500	0.01914	0.01019	0.17	0.33	114%	14%
3.82		0.01173	0.01537	0.00878	0.01293	0.01794	0.00929	0.17	0.33	110%	10%
7.6		0.00012	0.00028	0.00004	0.00009	0.00029	0.00002	0.17	0.33	72%	-28%
2.72		0.00175	0.00219	0.00152	0.00192	0.00226	0.00112	0.18	0.34	110%	10%
2.78		0.00187	0.00242	0.00130	0.00189	0.00252	0.00052	0.18	0.34	101%	1%
3.4		0.01277	0.01675	0.00946	0.01409	0.01782	0.01015	0.18	0.34	110%	10%
3.42		0.01322	0.01759	0.00963	0.01429	0.01827	0.00999	0.18	0.34	108%	8%
**2.00**	N-Acetyl of Glycoproteins	0.00592	0.00685	0.00539	0.00590	0.00655	0.00521	0.2	0.36	100%	0%
2.70		0.00129	0.00184	0.00110	0.00143	0.00176	0.00080	0.2	0.36	111%	11%
2.96		0.00156	0.00186	0.00103	0.00164	0.00190	0.00062	0.2	0.36	105%	5%
3.34		0.00226	0.00303	0.00138	0.00251	0.00311	0.00135	0.2	0.36	111%	11%
3.50		0.00501	0.00666	0.00292	0.00567	0.00777	0.00298	0.2	0.36	113%	13%
1.26		0.03732	0.04888	0.03040	0.03674	0.04522	0.02932	0.21	0.38	98%	-2%
2.74		0.00272	0.00363	0.00215	0.00275	0.00367	0.00143	0.23	0.4	101%	1%
3.00		0.00236	0.00292	0.00164	0.00245	0.00288	0.00189	0.23	0.4	104%	4%
3.38		0.00406	0.00549	0.00292	0.00447	0.00529	0.00311	0.23	0.4	110%	10%
7.74		0.00019	0.00036	0.00003	0.00016	0.00039	0.00004	0.24	0.42	85%	-15%
3.28		0.00373	0.00497	0.00268	0.00400	0.00467	0.00283	0.25	0.42	107%	7%
3.32		0.00141	0.00166	0.00108	0.00147	0.00166	0.00101	0.25	0.43	104%	4%
1.48		0.00582	0.00778	0.00429	0.00566	0.00765	0.00417	0.26	0.44	97%	-3%
2.98		0.00156	0.00186	0.00107	0.00165	0.00190	0.00068	0.26	0.44	105%	5%
3.02		0.00307	0.00364	0.00201	0.00314	0.00360	0.00224	0.28	0.47	102%	2%
1.98		0.00340	0.00377	0.00304	0.00347	0.00384	0.00315	0.3	0.5	102%	2%
3.08		0.00128	0.00153	0.00083	0.00132	0.00148	0.00055	0.3	0.5	104%	4%
2.94		0.00134	0.00160	0.00084	0.00142	0.00158	0.00045	0.31	0.5	106%	6%
4.20		0.00004	0.00047	0.00000	0.00004	0.00042	0.00001	0.32	0.52	91%	-9%
7.06		0.00087	0.00118	0.00055	0.00095	0.00114	0.00049	0.32	0.52	110%	10%
7.68		0.00023	0.00039	0.00006	0.00020	0.00045	0.00007	0.33	0.52	89%	-11%
0.78		0.00134	0.00177	0.00074	0.00133	0.00182	0.00073	0.35	0.54	99%	-1%
4.06		0.00093	0.00134	0.00066	0.00098	0.00130	0.00067	0.37	0.58	105%	5%
7.34		0.00034	0.00058	0.00007	0.00024	0.00055	0.00011	0.38	0.59	71%	-29%
**7.18 - 7.20, 688**	Tyrosine	0.00058	0.00085	0.00026	0.00049	0.00087	0.00027	0.39	0.59	84%	-16%
7.36		0.00032	0.00054	0.00008	0.00023	0.00054	0.00010	0.42	0.65	71%	-29%
6.86		0.00020	0.00044	0.00003	0.00020	0.00044	0.00003	0.44	0.66	101%	1%
8.00		0.00031	0.00051	0.00006	0.00035	0.00051	0.00002	0.44	0.66	114%	14%

1.06		0.00177	0.00209	0.00108	0.00175	0.00203	0.00040	0.46	0.68	99%	-1%
7.70		0.00014	0.00033	0.00003	0.00014	0.00035	0.00003	0.46	0.68	101%	1%
1.02		0.00239	0.00283	0.00153	0.00241	0.00270	0.00082	0.47	0.68	101%	1%
2.18		0.00123	0.00156	0.00096	0.00133	0.00148	0.00090	0.47	0.68	108%	8%
6.26		0.00003	0.00005	0.00001	0.00002	0.00004	0.00001	0.5	0.73	89%	-11%
2.92		0.00136	0.00163	0.00084	0.00139	0.00156	0.00036	0.52	0.74	102%	2%
4.28		0.00023	0.00052	0.00004	0.00026	0.00046	0.00003	0.52	0.74	113%	13%
**7.3 - 7.34**	Phenylalanine	0.00035	0.00054	0.00016	0.00023	0.00057	0.00014	0.52	0.74	65%	-35%
7.44		0.00011	0.00038	0.00003	0.00008	0.00031	0.00003	0.52	0.74	76%	-24%
0.94		0.00639	0.00711	0.00516	0.00618	0.00753	0.00501	0.58	0.8	97%	-3%
4.10		0.00178	0.00221	0.00131	0.00166	0.00233	0.00117	0.58	0.8	93%	-7%
6.74		0.00011	0.00028	0.00003	0.00007	0.00027	0.00004	0.58	0.8	68%	-32%
7.40		0.00034	0.00054	0.00018	0.00030	0.00052	0.00017	0.6	0.83	90%	-10%
2.54		0.00123	0.00183	0.00042	0.00123	0.00184	0.00067	0.61	0.83	100%	0%
1.94		0.00116	0.00153	0.00087	0.00114	0.00145	0.00083	0.64	0.85	98%	-2%
2.06		0.00923	0.01059	0.00809	0.00921	0.01028	0.00805	0.64	0.85	100%	0%
2.90		0.00137	0.00161	0.00086	0.00140	0.00159	0.00045	0.64	0.85	102%	2%
4.26		0.00017	0.00050	0.00003	0.00019	0.00044	0.00003	0.64	0.85	108%	8%
6.94		0.00037	0.00055	0.00009	0.00032	0.00060	0.00010	0.65	0.85	86%	-14%
0.98 - 1.03	L-Valine	0.00514	0.00704	0.00425	0.00517	0.00605	0.00442	0.7	0.9	101%	1%
7.10		0.00043	0.00073	0.00014	0.00043	0.00080	0.00010	0.7	0.9	100%	0%
7.84		0.00028	0.00045	0.00010	0.00031	0.00049	0.00010	0.7	0.9	112%	12%
0.96		0.00675	0.00953	0.00563	0.00694	0.00809	0.00585	0.73	0.92	103%	3%
6.72		0.00043	0.00060	0.00018	0.00041	0.00062	0.00027	0.73	0.92	95%	-5%
6.96		0.00040	0.00062	0.00017	0.00032	0.00061	0.00014	0.73	0.92	80%	-20%
7.20		0.00049	0.00076	0.00023	0.00039	0.00075	0.00024	0.73	0.92	81%	-19%
**5.24**	Lipids	0.00094	0.00112	0.00071	0.00100	0.00119	0.00063	0.74	0.92	107%	7%
5.26		0.00077	0.00102	0.00029	0.00080	0.00095	0.00019	0.76	0.94	104%	4%
1.96		0.00167	0.00189	0.00135	0.00170	0.00188	0.00141	0.77	0.94	101%	1%
6.90		0.00066	0.00096	0.00041	0.00064	0.00098	0.00042	0.77	0.94	97%	-3%
7.80		0.00137	0.00159	0.00067	0.00132	0.00178	0.00088	0.77	0.94	96%	-4%
1.72		0.00182	0.00260	0.00138	0.00180	0.00241	0.00133	0.78	0.94	99%	-1%
6.98		0.00033	0.00060	0.00005	0.00029	0.00054	0.00007	0.79	0.94	88%	-12%
**7.08*, 7.82 - 7.84**	Histidine	0.00091	0.00118	0.00040	0.00084	0.00119	0.00051	0.79	0.94	93%	-7%
2.66		0.00111	0.00158	0.00040	0.00103	0.00163	0.00049	0.81	0.96	93%	-7%
5.22		0.00085	0.00106	0.00066	0.00086	0.00104	0.00062	0.83	0.97	101%	1%
7.98		0.00026	0.00048	0.00004	0.00022	0.00050	0.00007	0.83	0.97	86%	-14%
**1.90**	Acetate	0.00107	0.00167	0.00074	0.00111	0.00146	0.00071	0.86	0.97	103%	3%
7.04		0.00070	0.00090	0.00035	0.00067	0.00091	0.00035	0.86	0.97	95%	-5%
1.00		0.00395	0.00552	0.00325	0.00396	0.00458	0.00329	0.87	0.97	100%	0%
1.52		0.00233	0.00261	0.00093	0.00236	0.00276	0.00067	0.87	0.97	101%	1%
1.70		0.00156	0.00214	0.00095	0.00155	0.00204	0.00090	0.87	0.97	99%	-1%
2.64		0.00086	0.00111	0.00045	0.00089	0.00115	0.00046	0.87	0.97	103%	3%
3.18		0.00277	0.00335	0.00215	0.00265	0.00364	0.00199	0.87	0.97	96%	-4%
6.24		0.00007	0.00011	0.00003	0.00007	0.00011	0.00003	0.87	0.97	103%	3%
1.04		0.00488	0.00791	0.00374	0.00503	0.00672	0.00404	0.9	0.99	103%	3%
2.20		0.00139	0.00160	0.00093	0.00142	0.00162	0.00102	0.91	0.99	102%	2%

4.12		0.00218	0.00297	0.00139	0.00198	0.00291	0.00134	0.91	0.99	91%	-9%
6.70		0.00031	0.00043	0.00022	0.00032	0.00046	0.00015	0.91	0.99	105%	5%
7.76		0.00050	0.00067	0.00024	0.00048	0.00069	0.00022	0.91	0.99	96%	-4%
3.14		0.00104	0.00155	0.00084	0.00105	0.00132	0.00052	0.93	>0.99	101%	1%
5.28		0.00125	0.00174	0.00062	0.00129	0.00156	0.00044	0.93	>0.99	104%	4%
7.86		0.00044	0.00076	0.00020	0.00043	0.00075	0.00021	0.93	>0.99	98%	-2%
1.50		0.00278	0.00324	0.00152	0.00269	0.00328	0.00123	0.97	>0.99	97%	-3%
7.32		0.00069	0.00093	0.00044	0.00066	0.00096	0.00044	0.97	>0.99	95%	-5%
7.42		0.00042	0.00064	0.00028	0.00040	0.00059	0.00023	0.97	>0.99	94%	-6%
Chemical Shift (^{1}H ppm)	Metabolite	Before	max	mim	After	max	mim	P value	q value	#	&
8.18*		0.00009	0.00016	0.00006	0.00006	0.00010	0.00003	0.001	0.05	65%	-35%
8.20*		0.00009	0.00017	0.00004	0.00006	0.00009	0.00001	0.001	0.05	72%	-28%
7.76*		0.00049	0.00081	0.00034	0.00070	0.00090	0.00036	0.002	0.1	142%	42%
1.00*, 0.94	Leucine	0.00422	0.00555	0.00281	0.00401	0.00477	0.00279	0.003	0.1	95%	-5%
1.32*, 4.10 - 4.12	Lactate	0.00273	0.00457	0.00194	0.00209	0.00333	0.00144	0.003	0.1	77%	-23%
7.74*		0.00020	0.00038	0.00003	0.00027	0.00043	0.00007	0.003	0.1	134%	34%
1.04*	Isobutyrate	0.00502	0.00757	0.00420	0.00476	0.00640	0.00390	0.005	0.1	95%	-5%
1.46* -1.48	Alanine	0.00592	0.00753	0.00445	0.00470	0.00646	0.00331	0.005	0.1	79%	-21%
7.10*		0.00060	0.00095	0.00028	0.00046	0.00084	0.00015	0.006	0.11	77%	-23%
2.28*		0.00233	0.00286	0.00152	0.00280	0.00376	0.00182	0.008	0.12	120%	20%
5.24*	Lipids	0.00092	0.00112	0.00065	0.00106	0.00126	0.00082	0.008	0.12	115%	15%
0.98* - 1.03	L-Valine	0.00547	0.00711	0.00417	0.00522	0.00600	0.00409	0.01	0.15	95%	-5%
1.72*		0.00213	0.00255	0.00149	0.00207	0.00249	0.00087	0.01	0.15	97%	-3%
2.22*		0.00245	0.00334	0.00185	0.00330	0.00466	0.00208	0.01	0.15	135%	35%
5.22* - 3.38	α-Glucose	0.00086	0.00122	0.00068	0.00091	0.00124	0.00079	0.01	0.15	107%	7%
7.20*		0.00053	0.00081	0.00025	0.00048	0.00070	0.00025	0.01	0.15	91%	-9%
8.00*		0.00048	0.00066	0.00019	0.00031	0.00064	0.00017	0.01	0.15	64%	-36%
1.90*	Acetate	0.00122	0.00153	0.00078	0.00117	0.00162	0.00024	0.02	0.15	96%	-4%
3.26*		0.01605	0.01901	0.01234	0.01506	0.01699	0.01222	0.02	0.15	94%	-6%
3.50*		0.00555	0.00708	0.00386	0.00680	0.00766	0.00500	0.02	0.17	122%	22%
6.72*		0.00056	0.00070	0.00039	0.00050	0.00062	0.00031	0.02	0.15	90%	-10%
6.86*		0.00023	0.00041	0.00004	0.00027	0.00038	0.00005	0.02	0.15	120%	20%
7.18 - 7.201 6.88*	Tyrosine	0.00064	0.00089	0.00033	0.00054	0.00077	0.00032	0.02	0.15	84%	-16%
1.34*		0.02932	0.05135	0.02347	0.02571	0.03680	0.01502	0.03	0.21	88%	-12%
1.7, 1.00*		0.00177	0.00213	0.00093	0.00173	0.00211	0.00048	0.03	0.22	98%	-2%
3.36*		0.00409	0.00507	0.00332	0.00435	0.00570	0.00341	0.03	0.22	106%	6%
7.44*		0.00013	0.00023	0.00004	0.00011	0.00032	0.00001	0.03	0.22	86%	-14%
1.2 - 1.28*	n(CH2) LipoPTNs	0.00758	0.00982	0.00557	0.00844	0.01069	0.00689	0.04	0.24	111%	11%
3.38*		0.00449	0.00620	0.00337	0.00478	0.00667	0.00417	0.04	0.24	106%	6%
7.82*		0.00098	0.00139	0.00058	0.00081	0.00132	0.00066	0.04	0.24	83%	-17%
7.84	Histidine	0.00039	0.00049	0.00011	0.00037	0.00055	0.00011	0.04	0.26	96%	-4%
7.98		0.00026	0.00041	0.00008	0.00036	0.00055	0.00005	0.05	0.27	142%	42%
2.30		0.00219	0.00234	0.00160	0.00232	0.00253	0.00165	0.06	0.3	106%	6%
2.38		0.00153	0.00178	0.00101	0.00167	0.00195	0.00112	0.06	0.32	109%	9%
3.64		0.00599	0.00689	0.00497	0.00644	0.00817	0.00520	0.06	0.3	108%	8%
6.90		0.00075	0.00106	0.00045	0.00072	0.00093	0.00045	0.06	0.3	96%	-4%

2.32		0.00218	0.00260	0.00174	0.00247	0.00269	0.00177	0.07	0.32	113%	13%
3.44		0.00800	0.01201	0.00578	0.00961	0.01160	0.00769	0.07	0.32	120%	20%
7.42		0.00048	0.00059	0.00033	0.00041	0.00059	0.00031	0.07	0.32	87%	-13%
7.88		0.00089	0.00107	0.00055	0.00078	0.00121	0.00049	0.07	0.32	88%	-12%
0.94		0.00606	0.00815	0.00452	0.00599	0.00658	0.00483	0.08	0.34	99%	-1%
0.96		0.00712	0.01076	0.00594	0.00683	0.00812	0.00573	0.08	0.34	96%	-4%
3.52		0.00759	0.01015	0.00498	0.00813	0.00925	0.00624	0.08	0.34	107%	7%
3.84		0.01060	0.01328	0.00709	0.01162	0.01331	0.00908	0.08	0.34	110%	10%
1.94		0.00127	0.00156	0.00086	0.00110	0.00164	0.00049	0.09	0.36	87%	-13%
3.48		0.01441	0.02310	0.00869	0.01555	0.01989	0.01204	0.09	0.36	108%	8%
3.74		0.01440	0.01890	0.00957	0.01556	0.01775	0.01204	0.09	0.36	108%	8%
3.86		0.00822	0.00984	0.00596	0.00913	0.01017	0.00694	0.09	0.36	111%	11%
5.26		0.00071	0.00085	0.00018	0.00072	0.00087	0.00022	0.09	0.36	102%	2%
7.66		0.00084	0.00116	0.00048	0.00068	0.00113	0.00046	0.09	0.36	81%	-19%
3.42		0.01516	0.02206	0.00964	0.01646	0.01975	0.01235	0.11	0.4	109%	9%
3.78		0.01080	0.01348	0.00707	0.01195	0.01390	0.00877	0.11	0.4	111%	11%
2.06		0.01004	0.01103	0.00912	0.00990	0.01096	0.00861	0.13	0.42	99%	-1%
2.36		0.00241	0.00291	0.00196	0.00227	0.00273	0.00172	0.13	0.42	94%	-6%
2.40		0.00166	0.00195	0.00113	0.00179	0.00209	0.00116	0.13	0.42	108%	8%
3.54		0.01074	0.01445	0.00719	0.01184	0.01311	0.00891	0.13	0.42	110%	10%
7.70		0.00021	0.00030	0.00002	0.00024	0.00033	0.00004	0.13	0.42	116%	16%
7.86		0.00047	0.00059	0.00029	0.00054	0.00092	0.00028	0.13	0.42	114%	14%
2.14		0.00506	0.00583	0.00386	0.00471	0.00602	0.00369	0.14	0.46	93%	-7%
2.16		0.00253	0.00327	0.00170	0.00261	0.00320	0.00204	0.15	0.46	103%	3%
7.68		0.00028	0.00037	0.00007	0.00031	0.00039	0.00009	0.15	0.46	112%	12%
1.5		0.00275	0.00352	0.00086	0.00280	0.00363	0.00089	0.17	0.48	102%	2%
2.02		0.01026	0.01175	0.00927	0.01037	0.01094	0.00938	0.17	0.48	101%	1%
2.10		0.00452	0.00549	0.00404	0.00461	0.00553	0.00386	0.17	0.48	102%	2%
3.40		0.01505	0.02257	0.00964	0.01616	0.01935	0.01229	0.17	0.48	107%	7%
3.88		0.01545	0.02141	0.01040	0.01740	0.01980	0.01310	0.17	0.48	113%	13%
6.94		0.00040	0.00056	0.00017	0.00046	0.00056	0.00018	0.17	0.48	115%	15%
5.28		0.00108	0.00139	0.00043	0.00114	0.00147	0.00048	0.19	0.53	105%	5%
7.60		0.00011	0.00028	0.00004	0.00011	0.00031	0.00004	0.19	0.53	100%	0%
5.78		0.00028	0.00047	0.00009	0.00034	0.00064	0.00014	0.2	0.54	122%	22%
**2.32 - 2.34**	Glutamate	0.00269	0.00316	0.00214	0.00255	0.00317	0.00199	0.22	0.56	95%	-5%
3.24		0.02154	0.02577	0.01587	0.02087	0.02608	0.01836	0.22	0.56	97%	-3%
**7.08, 7.82 - 7.84**	Histidine	0.00104	0.00129	0.00077	0.00097	0.00135	0.00063	0.22	0.56	94%	-6%
7.78		0.00123	0.00151	0.00102	0.00134	0.00158	0.00103	0.22	0.56	109%	9%
8.46		0.00009	0.00013	0.00005	0.00010	0.00014	0.00005	0.22	0.56	110%	10%
0.82		0.01007	0.01235	0.00938	0.01063	0.01199	0.00914	0.24	0.56	106%	6%
1.02		0.00259	0.00283	0.00070	0.00244	0.00261	0.00086	0.24	0.56	94%	-6%
2.12		0.00505	0.00583	0.00407	0.00486	0.00593	0.00393	0.24	0.56	96%	-4%
3.28		0.00412	0.00523	0.00302	0.00391	0.00512	0.00329	0.24	0.56	95%	-5%
3.46		0.01632	0.02457	0.00994	0.01724	0.02070	0.01365	0.24	0.56	106%	6%
3.70		0.01397	0.01808	0.00993	0.01561	0.01775	0.01154	0.24	0.56	112%	12%
5.80		0.00021	0.00035	0.00006	0.00024	0.00042	0.00007	0.24	0.56	113%	13%
7.34		0.00040	0.00055	0.00008	0.00040	0.00055	0.00008	0.24	0.56	102%	2%

7.64		0.00045	0.00077	0.00027	0.00051	0.00115	0.00030	0.24	0.56	111%	11%
5.76		0.00021	0.00034	0.00004	0.00024	0.00043	0.00006	0.25	0.57	114%	14%
2.26		0.00191	0.00299	0.00108	0.00206	0.00322	0.00156	0.27	0.6	108%	8%
2.72		0.00173	0.00219	0.00117	0.00177	0.00224	0.00119	0.27	0.6	102%	2%
3.22		0.03355	0.04853	0.02338	0.03266	0.04861	0.02371	0.27	0.6	97%	-3%
4.06		0.00094	0.00143	0.00079	0.00090	0.00118	0.00075	0.27	0.6	96%	-4%
7.40		0.00039	0.00047	0.00020	0.00035	0.00051	0.00021	0.3	0.63	89%	-11%
2.24		0.00237	0.00406	0.00124	0.00237	0.00405	0.00147	0.31	0.63	100%	0%
3.00		0.00268	0.00324	0.00170	0.00282	0.00328	0.00199	0.31	0.63	105%	5%
3.18		0.00301	0.00368	0.00181	0.00311	0.00375	0.00235	0.31	0.63	103%	3%
3.14		0.00115	0.00164	0.00001	0.00121	0.00162	0.00035	0.33	0.65	106%	6%
0.80		0.00294	0.00414	0.00200	0.00328	0.00392	0.00258	0.34	0.65	112%	12%
2.42		0.00228	0.00276	0.00168	0.00230	0.00311	0.00154	0.34	0.65	101%	1%
2.46		0.00298	0.00384	0.00214	0.00286	0.00424	0.00201	0.34	0.65	96%	-4%
2.94		0.00146	0.00190	0.00029	0.00161	0.00199	0.00053	0.34	0.65	110%	10%
3.66		0.00816	0.00958	0.00668	0.00865	0.01001	0.00682	0.34	0.65	106%	6%
3.72		0.01837	0.02711	0.01225	0.02005	0.02310	0.01519	0.34	0.65	109%	9%
7.32		0.00080	0.00098	0.00055	0.00074	0.00092	0.00048	0.34	0.65	93%	-7%
4.14		0.00045	0.00089	0.00008	0.00035	0.00066	0.00023	0.37	0.67	78%	-22%
2.08		0.00924	0.01073	0.00769	0.00888	0.01066	0.00735	0.38	0.67	96%	-4%
2.96		0.00180	0.00222	0.00055	0.00189	0.00224	0.00078	0.38	0.67	105%	5%
2.98		0.00178	0.00224	0.00054	0.00197	0.00222	0.00080	0.38	0.67	110%	10%
3.06		0.00159	0.00196	0.00018	0.00170	0.00200	0.00075	0.38	0.67	107%	7%
3.08		0.00136	0.00187	0.00014	0.00142	0.00176	0.00052	0.38	0.67	104%	4%
3.82		0.01424	0.02131	0.00930	0.01552	0.01761	0.01191	0.38	0.67	109%	9%
1.26		0.03251	0.04121	0.02369	0.03251	0.03936	0.02456	0.41	0.69	100%	0%
1.52		0.00226	0.00267	0.00026	0.00227	0.00275	0.00039	0.41	0.69	100%	0%
1.98		0.00335	0.00374	0.00280	0.00333	0.00357	0.00308	0.41	0.69	99%	-1%
2.20		0.00131	0.00159	0.00034	0.00135	0.00161	0.00092	0.41	0.69	103%	3%
3.92		0.00527	0.00683	0.00344	0.00542	0.00676	0.00413	0.41	0.69	103%	3%
3.96		0.00176	0.00225	0.00138	0.00168	0.00208	0.00146	0.41	0.69	95%	-5%
**7.3 - 7.34**	Phenylalanine	0.00038	0.00052	0.00018	0.00038	0.00056	0.00014	0.41	0.69	99%	-1%
8.22		0.00002	0.00005	0.00000	0.00002	0.00005	0.00001	0.41	0.69	95%	-5%
6.96		0.00044	0.00064	0.00020	0.00049	0.00057	0.00018	0.44	0.74	110%	10%
2.56		0.00077	0.00099	0.00017	0.00071	0.00097	0.00024	0.46	0.76	92%	-8%
7.04		0.00076	0.00100	0.00061	0.00075	0.00104	0.00046	0.49	0.79	100%	0%
7.36		0.00036	0.00048	0.00011	0.00038	0.00054	0.00010	0.5	0.8	105%	5%
**2.00**	N-Acetyl of Glycoproteins	0.00551	0.00678	0.00510	0.00554	0.00630	0.00509	0.54	0.84	101%	1%
2.44		0.00335	0.00448	0.00251	0.00337	0.00479	0.00256	0.54	0.84	101%	1%
3.60		0.00521	0.00595	0.00441	0.00503	0.00574	0.00432	0.54	0.84	96%	-4%
4.00		0.00174	0.00233	0.00139	0.00167	0.00194	0.00130	0.54	0.84	96%	-4%
4.02		0.00079	0.00140	0.00038	0.00077	0.00098	0.00042	0.54	0.84	98%	-2%
6.70		0.00037	0.00047	0.00026	0.00037	0.00044	0.00022	0.58	0.85	101%	1%
0.84		0.02053	0.02443	0.01719	0.02162	0.02375	0.01621	0.59	0.85	105%	5%
**2.5, 2.53–2.54, 2.65, 2.68**	Citrate	0.00098	0.00114	0.00023	0.00082	0.00122	0.00041	0.59	0.85	84%	-16%

2.76		0.00186	0.00292	0.00068	0.00191	0.00277	0.00125	0.59	0.85	103%	3%
2.90		0.00148	0.00199	0.00039	0.00157	0.00205	0.00062	0.59	0.85	106%	6%
3.34		0.00243	0.00334	0.00160	0.00244	0.00333	0.00201	0.59	0.85	100%	0%
3.90		0.00942	0.01074	0.00661	0.00937	0.01064	0.00782	0.59	0.85	99%	-1%
5.32		0.00091	0.00203	0.00031	0.00110	0.00192	0.00032	0.59	0.85	121%	21%
6.24		0.00008	0.00016	0.00006	0.00008	0.00012	0.00003	0.59	0.85	92%	-8%
1.42		0.00251	0.00313	0.00032	0.00268	0.00305	0.00080	0.64	0.89	107%	7%
1.54		0.00209	0.00286	0.00006	0.00213	0.00273	0.00019	0.64	0.89	102%	2%
1.92		0.00170	0.00229	0.00130	0.00173	0.00282	0.00098	0.64	0.89	102%	2%
1.96		0.00163	0.00193	0.00112	0.00162	0.00192	0.00105	0.64	0.89	99%	-1%
4.26		0.00010	0.00050	0.00005	0.00009	0.00044	0.00005	0.64	0.89	96%	-4%
6.74		0.00011	0.00030	0.00005	0.00013	0.00025	0.00004	0.65	0.9	116%	16%
0.90		0.01182	0.02378	0.00773	0.01284	0.02177	0.00797	0.68	0.92	109%	9%
1.40		0.00303	0.00372	0.00073	0.00312	0.00373	0.00131	0.68	0.92	103%	3%
2.52		0.00123	0.00157	0.00072	0.00110	0.00161	0.00071	0.68	0.92	89%	-11%
3.20		0.01823	0.02076	0.01645	0.01897	0.02163	0.01575	0.68	0.92	104%	4%
**3.98**	Phosphoet hanolamine	0.00161	0.00213	0.00115	0.00159	0.00185	0.00127	0.68	0.92	99%	-1%
4.20		0.00005	0.00049	0.00000	0.00003	0.00035	0.00001	0.68	0.92	61%	-39%
1.06		0.00191	0.00213	0.00030	0.00184	0.00198	0.00042	0.74	0.95	96%	-4%
1.30		0.03069	0.05848	0.01850	0.03263	0.05162	0.01773	0.74	0.95	106%	6%
1.36		0.00594	0.01053	0.00356	0.00486	0.00957	0.00345	0.74	0.95	82%	-18%
2.74		0.00222	0.00344	0.00156	0.00239	0.00319	0.00171	0.74	0.95	107%	7%
2.92		0.00152	0.00197	0.00042	0.00154	0.00194	0.00047	0.74	0.95	102%	2%
**3.58, 4.26, 1.34**	Threonine	0.00641	0.00716	0.00473	0.00625	0.00769	0.00498	0.74	0.95	98%	-2%
2.18		0.00136	0.00166	0.00026	0.00130	0.00163	0.00074	0.75	0.95	95%	-5%
**2.44 - 2.48, 2.10**	Glutamine	0.00151	0.00195	0.00078	0.00133	0.00204	0.00102	0.75	0.95	88%	-12%
2.66		0.00133	0.00192	0.00052	0.00115	0.00185	0.00056	0.75	0.95	87%	-13%
1.56		0.00186	0.00387	0.00038	0.00197	0.00357	0.00023	0.79	0.97	106%	6%
6.26		0.00003	0.00004	0.00001	0.00003	0.00005	0.00001	0.79	0.97	107%	7%
7.8		0.00154	0.00206	0.00132	0.00152	0.00212	0.00102	0.79	0.97	98%	-2%
3.32		0.00141	0.00187	0.00030	0.00143	0.00190	0.00081	0.83	>0.99	102%	2%
1.38		0.00335	0.00504	0.00083	0.00312	0.00501	0.00144	0.84	>0.99	93%	-7%
1.58		0.00176	0.00394	0.00011	0.00186	0.00377	0.00006	0.84	>0.99	105%	5%
2.64		0.00101	0.00124	0.00030	0.00086	0.00134	0.00040	0.84	>0.99	85%	-15%
3.02		0.00326	0.00390	0.00257	0.00330	0.00411	0.00262	0.84	>0.99	101%	1%
3.30		0.00197	0.00252	0.00079	0.00198	0.00256	0.00145	0.84	>0.99	101%	1%
3.56		0.01053	0.01175	0.00743	0.01097	0.01216	0.00766	0.84	>0.99	104%	4%
3.80		0.00508	0.00595	0.00381	0.00515	0.00598	0.00437	0.84	>0.99	101%	1%
4.04		0.00061	0.00112	0.00026	0.00059	0.00083	0.00035	0.85	>0.99	97%	-3%
1.24		0.02175	0.02421	0.01859	0.02172	0.02425	0.01838	0.89	>0.99	100%	0%
2.04		0.02230	0.02782	0.01995	0.02284	0.02777	0.01851	0.89	>0.99	102%	2%
2.78		0.00158	0.00232	0.00045	0.00155	0.00215	0.00070	0.89	>0.99	98%	-2%
3.94		0.00248	0.00307	0.00189	0.00239	0.00296	0.00201	0.89	>0.99	96%	-4%
5.30		0.00148	0.00231	0.00060	0.00146	0.00211	0.00054	0.89	>0.99	99%	-1%
0.92		0.00539	0.00709	0.00386	0.00515	0.00739	0.00445	0.91	>0.99	96%	-4%
0.86		0.02600	0.02962	0.02051	0.02564	0.02947	0.02092	0.95	>0.99	99%	-1%
1.22		0.01357	0.01612	0.01155	0.01347	0.01544	0.01211	0.95	>0.99	99%	-1%

3.04		0.00476	0.00661	0.00366	0.00505	0.00690	0.00360	0.95	>0.99	106%	6%
3.68		0.00792	0.01000	0.00625	0.00803	0.00964	0.00653	0.95	>0.99	101%	1%
3.76		0.01093	0.01382	0.00745	0.01124	0.01282	0.00879	0.95	>0.99	103%	3%
6.98		0.00037	0.00057	0.00012	0.00041	0.00047	0.00006	0.95	>0.99	110%	10%
2.54		0.00157	0.00206	0.00082	0.00135	0.00207	0.00057	0.97	>0.99	86%	-14%
3.62		0.00475	0.00526	0.00384	0.00472	0.00534	0.00388	0.99	>0.99	99%	-1%
0.78		0.00128	0.00190	0.00037	0.00128	0.00179	0.00069	>0.99	>0.99	99%	-1%
**0.88 - 0.86**	n(CH3) LipoPTNs	0.02400	0.03845	0.01528	0.02464	0.03598	0.01687	>0.99	>0.99	103%	3%
2.70		0.00131	0.00208	0.00039	0.00135	0.00187	0.00091	>0.99	>0.99	103%	3%
2.80		0.00123	0.00181	0.00008	0.00115	0.00167	0.00023	>0.99	>0.99	94%	-6%
4.28		0.00008	0.00046	0.00003	0.00009	0.00048	0.00004	>0.99	>0.99	108%	8%
7.06		0.00102	0.00128	0.00078	0.00097	0.00132	0.00082	>0.99	>0.99	95%	-5%

Table.S3 Table of chemical shifts used for relative quantification of saliva metabolites from Instructors and Trainees. Paired analysis, carried out with the Wilcoxon test nonparametric, data expressed as median, minimum and maximum after Shapiro-Wilk normality test. # corresponds to instructors over trainees. & corresponds to the % from #. *Values less than p < 0.05.

Instructors											
Chemical Shift (^1H ppm)	Metabolite	Before	max	mim	After	max	mim	P value	q value	#	&
3.26*		0.191	0.316	0.122	0.136	0.285	0.099	0.005	0.26	71%	-28.7%
3.96*		0.517	0.958	0.277	0.360	0.614	0.249	0.005	0.26	70%	-30.4%
3.22*	n$(CH_3)_3$ Choline	0.226	0.409	0.164	0.174	0.372	0.112	0.007	0.26	77%	-23.1%
3.24*		0.271	0.419	0.167	0.186	0.478	0.125	0.011	0.26	69%	-31.3%
5.62*		0.025	0.076	0.003	0.013	0.034	0.003	0.013	0.26	55%	-45.1%
8.22*		0.026	0.047	0.016	0.021	0.036	0.011	0.013	0.26	82%	-17.9%
8.96*		0.005	0.010	0.002	0.004	0.011	0.001	0.013	0.26	81%	-18.9%
2.66*-2.64	R-Malic acid	0.100	0.152	0.062	0.084	0.165	0.056	0.017	0.26	84%	-16.2%
3.92*		0.451	0.697	0.228	0.334	0.764	0.209	0.017	0.26	74%	-26.0%
5.64*		0.030	0.093	0.001	0.015	0.041	0.001	0.017	0.26	48%	-51.5%
3.28*	L-Cysteic acid	0.233	0.429	0.135	0.198	0.296	0.100	0.02	0.26	85%	-15.0%
5.78*		0.207	0.633	0.004	0.095	0.299	0.000	0.02	0.26	46%	-54.3%
7.98*		0.034	0.075	0.016	0.031	0.045	0.016	0.02	0.26	91%	-9.2%
2.78*		0.111	0.217	0.059	0.090	0.225	0.055	0.025	0.26	80%	-19.6%
3.90*		0.444	0.727	0.225	0.346	0.829	0.215	0.025	0.26	78%	-22.0%
5.84*		0.138	0.415	0.005	0.063	0.199	0.001	0.025	0.26	46%	-54.0%
5.88*		0.065	0.192	0.004	0.030	0.093	0.001	0.025	0.26	47%	-53.5%
2.80*		0.119	0.215	0.057	0.097	0.221	0.056	0.03	0.26	82%	-18.1%
2.84*		0.086	0.166	0.045	0.075	0.173	0.041	0.03	0.26	87%	-12.6%
3.62*		0.531	0.900	0.256	0.439	0.915	0.261	0.03	0.26	83%	-17.3%
3.68*		0.597	0.902	0.254	0.509	1.067	0.279	0.03	0.26	85%	-14.7%
3.7*		0.455	0.704	0.206	0.378	0.735	0.206	0.03	0.26	83%	-16.8%
3.82*		0.721	1.056	0.306	0.578	1.063	0.316	0.03	0.26	80%	-19.8%
3.84*		0.594	1.012	0.311	0.510	1.135	0.314	0.03	0.26	86%	-14.1%
3.88*		0.514	0.776	0.248	0.419	0.927	0.253	0.03	0.26	82%	-18.5%
5.80*		0.228	0.700	0.004	0.105	0.330	0.000	0.03	0.26	46%	-54.1%
5.82*		0.194	0.591	0.003	0.089	0.281	0.001	0.03	0.26	46%	-54.4%
2.56*	Acetylcarnitine	0.101	0.174	0.058	0.077	0.151	0.050	0.035	0.26	77%	-23.2%
2.82*		0.108	0.205	0.053	0.094	0.214	0.049	0.035	0.26	87%	-13.2%
3.72*		0.433	0.767	0.192	0.337	0.625	0.188	0.035	0.26	78%	-22.1%
4.02*		0.267	0.492	0.145	0.215	0.583	0.148	0.035	0.26	80%	-19.5%
7.60*		0.067	0.143	0.035	0.045	0.166	0.027	0.035	0.26	67%	-32.5%
7.96*		0.033	0.061	0.014	0.023	0.041	0.015	0.035	0.26	70%	-29.7%
2.86*		0.089	0.180	0.048	0.071	0.179	0.044	0.042	0.26	81%	-19.5%
3.60*		0.437	0.749	0.201	0.346	0.712	0.219	0.042	0.26	79%	-20.9%
4.00*		0.481	0.811	0.235	0.351	1.037	0.229	0.042	0.26	73%	-27.0%
4.06*		0.119	0.229	0.071	0.105	0.212	0.060	0.042	0.26	88%	-12.1%
4.08*		0.109	0.199	0.065	0.093	0.153	0.050	0.042	0.26	85%	-14.7%

**4.10* - 4.12, 1.32**	**Lactate**	**0.099**	**0.178**	**0.054**	**0.084**	**0.141**	**0.046**	**0.042**	**0.26**	**85%**	**-15.0%**
**7.58***		**0.084**	**0.179**	**0.045**	**0.054**	**0.210**	**0.033**	**0.042**	**0.26**	**65%**	**-35.5%**
**7.66***		**0.032**	**0.064**	**0.020**	**0.025**	**0.053**	**0.017**	**0.042**	**0.26**	**80%**	**-20.2%**
**7.84***		**0.051**	**0.119**	**0.026**	**0.033**	**0.089**	**0.022**	**0.042**	**0.26**	**65%**	**-35.0%**
**8.26***		**0.018**	**0.031**	**0.012**	**0.016**	**0.025**	**0.009**	**0.042**	**0.26**	**88%**	**-11.6%**
**1.4***		**0.122**	**0.249**	**0.076**	**0.106**	**0.214**	**0.059**	**0.049**	**0.26**	**87%**	**-13.1%**
**1.66***		**0.529**	**1.474**	**0.220**	**0.601**	**1.704**	**0.208**	**0.049**	**0.26**	**114 %**	**13.6%**
**3.64***		**0.652**	**0.992**	**0.271**	**0.555**	**1.183**	**0.300**	**0.049**	**0.26**	**85%**	**-14.9%**
**3.74***		**0.485**	**0.891**	**0.213**	**0.376**	**0.688**	**0.210**	**0.049**	**0.26**	**78%**	**-22.4%**
**4.04***		**0.168**	**0.300**	**0.084**	**0.151**	**0.324**	**0.080**	**0.049**	**0.26**	**90%**	**-10.1%**
**7.54***		**0.060**	**0.127**	**0.034**	**0.045**	**0.123**	**0.029**	**0.049**	**0.26**	**76%**	**-24.0%**
**8.20***		**0.024**	**0.045**	**0.016**	**0.021**	**0.034**	**0.012**	**0.049**	**0.26**	**91%**	**-9.4%**
**8.34***		**0.024**	**0.049**	**0.018**	**0.020**	**0.038**	**0.011**	**0.049**	**0.26**	**84%**	**-16.4%**
8.24		0.021	0.034	0.011	0.017	0.035	0.009	0.057	0.26	81%	-18.9%
1.42		0.159	0.286	0.082	0.124	0.341	0.071	0.058	0.26	78%	-22.2%
2.06		2.180	3.486	0.980	1.937	4.029	1.053	0.058	0.26	89%	-11.2%
2.44		0.492	0.861	0.193	0.377	1.169	0.214	0.058	0.26	77%	-23.3%
2.76		0.138	0.261	0.082	0.120	0.268	0.068	0.058	0.26	87%	-13.1%
3.66		0.732	1.099	0.304	0.620	1.362	0.335	0.058	0.26	85%	-15.3%
3.86		0.668	1.004	0.294	0.553	1.156	0.311	0.058	0.26	83%	-17.3%
5.28		0.008	0.029	0.000	0.006	0.017	0.000	0.058	0.26	71%	-29.3%
7.62		0.044	0.093	0.023	0.033	0.092	0.019	0.058	0.26	75%	-24.6%
8.32		0.025	0.055	0.020	0.024	0.039	0.013	0.058	0.26	94%	-5.7%
8.36		0.021	0.046	0.016	0.020	0.040	0.012	0.058	0.26	92%	-7.9%
2.04		1.476	2.759	0.657	1.313	3.211	0.733	0.068	0.28	89%	-11.1%
2.4		0.638	1.251	0.335	0.481	1.599	0.286	0.068	0.28	75%	-24.6%
2.72		0.134	0.250	0.081	0.117	0.268	0.069	0.068	0.28	87%	-13.0%
7.56		0.085	0.169	0.047	0.061	0.194	0.034	0.068	0.28	72%	-28.1%
7.64		0.036	0.080	0.021	0.030	0.063	0.020	0.068	0.28	82%	-18.0%
8.12		0.031	0.078	0.018	0.024	0.048	0.018	0.068	0.28	79%	-21.1%
1.06		0.795	2.579	0.250	1.020	2.168	0.283	0.078	0.28	128 %	28.3%
2.38		0.767	1.438	0.384	0.695	1.695	0.367	0.078	0.28	91%	-9.4%
**2.54-2.53, 2.5, 2.65, 2.68**	Citrate	0.081	0.155	0.046	0.065	0.132	0.039	0.078	0.28	81%	-19.5%
2.68		0.101	0.167	0.055	0.078	0.159	0.041	0.078	0.28	78%	-22.2%
3.2		0.139	0.288	0.104	0.122	0.247	0.075	0.078	0.28	88%	-11.7%
3.8		0.665	1.098	0.283	0.545	0.961	0.292	0.078	0.28	82%	-18.0%
7.88		0.025	0.047	0.016	0.021	0.033	0.013	0.078	0.28	82%	-17.9%
8.28		0.019	0.038	0.013	0.016	0.027	0.009	0.078	0.28	85%	-15.2%
8.3		0.023	0.050	0.016	0.020	0.035	0.011	0.078	0.28	89%	-10.8%
8.4		0.027	0.057	0.020	0.023	0.046	0.013	0.078	0.28	83%	-17.0%
1.98		0.811	1.600	0.371	0.672	1.684	0.402	0.091	0.30	83%	-17.1%
2.12		0.715	1.384	0.339	0.584	1.289	0.335	0.091	0.30	82%	-18.4%
2.7		0.118	0.197	0.066	0.098	0.209	0.056	0.091	0.30	83%	-17.3%
3.5		0.207	0.402	0.078	0.158	0.327	0.083	0.091	0.30	77%	-23.4%
3.52		0.242	0.538	0.122	0.214	0.367	0.128	0.091	0.30	88%	-11.8%

**3.98**	Phosphoethanolamine.	0.408	0.717	0.253	0.332	0.866	0.232	0.091	0.30	81%	-18.5%
**1.04**	Isobutyrate	0.208	0.631	0.084	0.244	0.521	0.092	0.104	0.32	117%	17.1%
1.38		0.129	0.314	0.082	0.111	0.239	0.060	0.104	0.32	86%	-14.0%
2.32		0.623	1.331	0.306	0.567	1.399	0.345	0.104	0.32	91%	-9.0%
2.74		0.139	0.264	0.100	0.124	0.276	0.082	0.104	0.32	89%	-10.7%
8.02		0.042	0.093	0.027	0.033	0.078	0.021	0.104	0.32	78%	-21.6%
8.14		0.022	0.053	0.014	0.022	0.038	0.011	0.104	0.32	103%	3.3%
1.3		0.109	0.235	0.038	0.085	0.135	0.040	0.119	0.33	78%	-21.9%
1.64		0.376	0.897	0.165	0.392	1.050	0.160	0.119	0.33	104%	4.3%
2.08		1.667	2.892	0.837	1.530	3.255	0.912	0.119	0.33	92%	-8.2%
2.1		0.952	1.887	0.445	0.778	1.801	0.466	0.119	0.33	82%	-18.3%
2.3		0.492	1.031	0.246	0.440	1.085	0.258	0.119	0.33	89%	-10.6%
6.9		0.208	0.440	0.129	0.180	0.449	0.104	0.119	0.33	86%	-13.6%
8		0.037	0.083	0.019	0.035	0.058	0.018	0.119	0.33	95%	-4.8%
8.04		0.049	0.118	0.030	0.039	0.090	0.026	0.119	0.33	79%	-21.2%
8.06		0.070	0.179	0.043	0.055	0.129	0.039	0.119	0.33	79%	-21.2%
8.38		0.022	0.047	0.015	0.018	0.039	0.010	0.119	0.33	81%	-19.1%
1.86		0.217	0.471	0.115	0.200	0.506	0.125	0.131	0.36	92%	-7.7%
**6.88, 7.18 - 7.20**	Tyrosine	0.168	0.394	0.103	0.147	0.349	0.093	0.135	0.37	87%	-12.6%
8.42		0.029	0.060	0.020	0.024	0.054	0.011	0.135	0.37	81%	-18.5%
1.96		0.972	1.980	0.470	0.862	2.035	0.482	0.153	0.39	89%	-11.4%
2.24		0.712	1.741	0.330	0.760	1.882	0.317	0.153	0.39	107%	6.7%
3.54		0.274	0.601	0.141	0.233	0.379	0.146	0.153	0.39	85%	-14.9%
7.48		0.017	0.041	0.012	0.016	0.039	0.010	0.153	0.39	94%	-6.3%
7.92		0.035	0.142	0.019	0.031	0.073	0.016	0.153	0.39	89%	-11.4%
8.1		0.039	0.123	0.027	0.043	0.080	0.028	0.153	0.39	109%	9.4%
8.98		0.004	0.007	0.001	0.002	0.013	0.001	0.153	0.39	59%	-41.2%
1.08		0.705	2.517	0.241	0.921	1.992	0.265	0.173	0.41	131%	30.7%
1.44		0.173	0.331	0.105	0.158	0.362	0.083	0.173	0.41	91%	-8.8%
1.84		0.219	0.451	0.115	0.207	0.517	0.129	0.173	0.41	95%	-5.3%
2		0.845	1.686	0.382	0.701	1.754	0.411	0.173	0.41	83%	-17.0%
2.62		0.085	0.188	0.056	0.081	0.151	0.042	0.173	0.41	96%	-4.0%
3.16		0.204	0.298	0.113	0.172	0.328	0.092	0.173	0.41	85%	-15.4%
3.78		0.780	1.549	0.347	0.670	1.296	0.380	0.173	0.41	86%	-14.1%
6.92		0.200	0.406	0.136	0.177	0.406	0.098	0.173	0.41	88%	-11.7%
8.08		0.071	0.176	0.046	0.058	0.139	0.042	0.173	0.41	82%	-18.3%
1.36		0.172	2.368	0.073	0.130	0.435	0.062	0.194	0.42	76%	-24.3%
1.68		0.540	1.271	0.224	0.557	1.444	0.224	0.194	0.42	103%	3.2%
1.72		0.449	0.936	0.243	0.415	1.090	0.229	0.194	0.42	92%	-7.6%
2.14		0.670	1.280	0.314	0.574	1.239	0.316	0.194	0.42	86%	-14.4%
2.28		0.397	0.820	0.200	0.361	0.838	0.199	0.194	0.42	91%	-8.9%
2.88		0.067	0.148	0.043	0.062	0.148	0.036	0.194	0.42	92%	-7.9%
6.68		0.014	0.038	0.007	0.012	0.028	0.005	0.194	0.42	86%	-14.2%
7.06		0.044	0.121	0.025	0.038	0.070	0.019	0.194	0.42	86%	-13.6%

**7.08, 7.82**	Histidine	0.087	0.194	0.042	0.077	0.119	0.038	0.194	0.42	89%	-11.4%
7.52		0.036	0.082	0.021	0.031	0.073	0.018	0.194	0.42	88%	-12.4%
2.02		1.048	2.146	0.482	0.934	2.305	0.529	0.217	0.45	89%	-10.9%
3.94		0.588	0.846	0.277	0.480	1.061	0.337	0.217	0.45	82%	-18.3%
4.16		0.093	0.263	0.048	0.081	0.171	0.047	0.217	0.45	87%	-13.3%
6.86		0.153	0.392	0.109	0.137	0.287	0.088	0.217	0.45	90%	-10.3%
7.68		0.019	0.041	0.013	0.016	0.034	0.009	0.217	0.45	85%	-14.8%
8.18		0.021	0.048	0.014	0.019	0.035	0.012	0.217	0.45	91%	-9.3%
**8.46**	Formate	0.034	0.817	0.017	0.215	0.512	0.011	0.217	0.45	630 %	529.8 %
2.58		0.086	0.185	0.051	0.074	0.146	0.038	0.241	0.49	86%	-13.8%
6.7		0.015	0.037	0.007	0.013	0.028	0.005	0.241	0.49	87%	-13.4%
6.72		0.016	0.041	0.008	0.013	0.029	0.007	0.241	0.49	84%	-16.4%
7.94		0.024	0.055	0.011	0.019	0.040	0.010	0.241	0.49	80%	-19.6%
1.74		0.425	0.879	0.235	0.389	0.947	0.211	0.268	0.51	92%	-8.4%
1.78		0.359	1.067	0.182	0.414	0.953	0.188	0.268	0.51	115 %	15.2%
1.88		0.248	0.533	0.134	0.240	0.543	0.137	0.268	0.51	97%	-3.5%
**2.34**	Glutamate	0.782	1.790	0.382	0.739	1.785	0.418	0.268	0.51	94%	-5.5%
2.46		0.272	0.539	0.119	0.235	0.571	0.116	0.268	0.51	86%	-13.7%
2.6		0.082	0.172	0.052	0.074	0.140	0.038	0.268	0.51	90%	-9.6%
7.1		0.047	0.109	0.030	0.045	0.094	0.021	0.268	0.51	96%	-3.8%
7.5		0.025	0.059	0.017	0.022	0.054	0.013	0.268	0.51	88%	-12.1%
1.82		0.219	0.447	0.104	0.193	0.474	0.106	0.296	0.53	88%	-11.8%
1.9		0.364	0.760	0.195	0.340	0.779	0.200	0.296	0.53	93%	-6.7%
2.36		0.743	1.697	0.371	0.700	1.683	0.423	0.296	0.53	94%	-5.8%
2.52		0.074	0.167	0.043	0.067	0.137	0.035	0.296	0.53	90%	-9.6%
3.14		0.162	0.310	0.102	0.142	0.249	0.073	0.296	0.53	88%	-12.2%
3.18		0.122	0.232	0.070	0.109	0.192	0.054	0.296	0.53	89%	-10.7%
**4.12**	Lactate	0.090	0.183	0.047	0.078	0.148	0.042	0.296	0.53	87%	-12.9%
4.14		0.085	0.177	0.045	0.073	0.148	0.043	0.296	0.53	85%	-14.5%
6.74		0.022	0.050	0.012	0.017	0.035	0.009	0.296	0.53	81%	-19.1%
6.78		0.026	0.066	0.017	0.024	0.057	0.013	0.296	0.53	93%	-6.7%
7.7		0.014	0.038	0.008	0.013	0.031	0.006	0.296	0.53	89%	-10.8%
**1.46 -1.48**	Alanine	0.191	0.389	0.112	0.181	0.418	0.088	0.326	0.54	95%	-4.9%
2.94		0.062	0.133	0.039	0.059	0.130	0.031	0.326	0.54	95%	-5.4%
3.04		0.649	1.583	0.305	0.701	1.707	0.304	0.326	0.54	108 %	8.0%
3.3		0.116	0.293	0.045	0.122	0.238	0.049	0.326	0.54	105 %	5.1%
6.76		0.024	0.059	0.015	0.022	0.045	0.011	0.326	0.54	89%	-11.2%
6.8		0.038	0.088	0.022	0.033	0.084	0.019	0.326	0.54	86%	-13.8%
6.82		0.069	0.154	0.040	0.061	0.154	0.034	0.326	0.54	89%	-11.2%
6.84		0.098	0.253	0.065	0.095	0.214	0.057	0.326	0.54	97%	-2.9%
7.12		0.056	0.147	0.035	0.056	0.101	0.030	0.326	0.54	100 %	-0.2%
**0.94**	Leucine	0.465	1.002	0.317	0.451	0.823	0.241	0.358	0.57	97%	-3.1%
1.02		0.134	0.401	0.065	0.164	0.307	0.064	0.358	0.57	123 %	22.7%
1.5		0.285	0.626	0.151	0.257	0.607	0.131	0.358	0.57	90%	-9.9%
2.92		0.058	0.139	0.040	0.061	0.122	0.031	0.358	0.57	105 %	5.5%

3.12		0.115	0.240	0.077	0.105	0.205	0.057	0.358	0.57	91%	-8.6%
6.64		0.011	0.030	0.005	0.010	0.022	0.003	0.358	0.57	92%	-8.2%
6.96		0.059	0.140	0.037	0.049	0.144	0.030	0.358	0.57	82%	-17.8%
7.34		0.126	0.309	0.087	0.144	0.221	0.073	0.358	0.57	114%	14.2%
0.9		0.421	0.883	0.281	0.462	0.831	0.224	0.391	0.61	110%	9.6%
3.1		0.118	0.270	0.079	0.120	0.227	0.059	0.391	0.61	101%	1.3%
6.66		0.013	0.034	0.006	0.011	0.025	0.004	0.391	0.61	89%	-10.6%
7.38		0.075	0.233	0.048	0.091	0.160	0.043	0.391	0.61	121%	21.5%
0.96		0.523	1.018	0.322	0.441	0.714	0.224	0.426	0.63	84%	-15.7%
1.8		0.304	0.830	0.151	0.329	0.730	0.150	0.426	0.63	108%	8.2%
3.44		0.226	0.528	0.123	0.236	0.515	0.096	0.426	0.63	104%	4.3%
**3.58, 4.26, 1.34**	Threonine	0.667	1.749	0.309	0.697	1.893	0.341	0.426	0.63	104%	4.5%
6.94		0.099	0.215	0.059	0.086	0.249	0.055	0.426	0.63	87%	-13.3%
6.98		0.038	0.086	0.021	0.032	0.104	0.016	0.426	0.63	83%	-17.2%
7.04		0.054	0.102	0.022	0.046	0.115	0.024	0.426	0.63	84%	-16.1%
7.26		0.066	0.164	0.040	0.069	0.130	0.032	0.426	0.63	105%	4.7%
7.9		0.022	0.068	0.015	0.020	0.037	0.011	0.426	0.63	92%	-7.9%
1.24		0.171	0.400	0.061	0.145	0.334	0.066	0.463	0.65	85%	-15.1%
1.28		0.174	0.409	0.044	0.143	0.302	0.055	0.463	0.65	82%	-17.8%
2.9		0.079	0.190	0.051	0.086	0.156	0.039	0.463	0.65	109%	8.5%
2.96		0.082	0.174	0.050	0.074	0.186	0.040	0.463	0.65	91%	-9.3%
2.98		0.125	0.277	0.076	0.112	0.288	0.061	0.463	0.65	90%	-10.0%
3.34		0.086	0.299	0.049	0.105	0.296	0.054	0.463	0.65	123%	23.2%
3.36		0.094	0.344	0.046	0.111	0.353	0.049	0.463	0.65	117%	17.3%
3.76		0.729	1.481	0.314	0.615	1.280	0.349	0.463	0.65	84%	-15.5%
6.62		0.010	0.030	0.005	0.009	0.021	0.003	0.463	0.65	92%	-8.0%
7.16		0.062	0.162	0.039	0.059	0.110	0.028	0.463	0.65	95%	-4.8%
7.4		0.066	0.176	0.032	0.076	0.137	0.035	0.463	0.65	117%	16.7%
7.46		0.026	0.058	0.015	0.025	0.040	0.015	0.463	0.65	95%	-5.0%
2.16		0.683	1.301	0.315	0.588	1.182	0.318	0.502	0.69	86%	-13.9%
3		0.221	0.511	0.134	0.225	0.598	0.119	0.502	0.69	102%	1.8%
7.32		0.089	0.272	0.057	0.099	0.194	0.052	0.502	0.69	112%	11.8%
8.16		0.022	0.063	0.013	0.023	0.035	0.014	0.502	0.69	106%	5.7%
1.52		0.187	0.403	0.102	0.168	0.379	0.082	0.542	0.72	90%	-10.2%
3.06		0.425	1.038	0.228	0.469	0.946	0.212	0.542	0.72	110%	10.2%
6.6		0.008	0.026	0.004	0.008	0.018	0.002	0.542	0.72	93%	-6.7%
7.02		0.043	0.110	0.028	0.042	0.079	0.022	0.542	0.72	99%	-0.8%
7.24		0.044	0.101	0.021	0.038	0.083	0.018	0.542	0.72	86%	-14.0%
0.98 - 1.03	L-Valine	0.321	0.693	0.181	0.312	0.542	0.135	0.583	0.75	97%	-2.6%
1		0.163	0.387	0.085	0.172	0.323	0.063	0.583	0.75	106%	5.7%
1.22		0.273	0.587	0.132	0.267	0.547	0.126	0.583	0.75	98%	-2.0%
1.62		0.234	0.506	0.114	0.224	0.514	0.121	0.583	0.75	96%	-4.3%

1.92		1.614	3.550	0.736	1.649	2.972	0.812	0.583	0.75	102 %	2.1%
7		0.057	0.114	0.027	0.052	0.137	0.028	0.583	0.75	91%	-8.6%
0.8		0.039	0.115	0.019	0.043	0.084	0.019	0.626	0.76	111 %	10.6%
0.92		0.342	0.775	0.215	0.354	0.668	0.178	0.626	0.76	103 %	3.4%
1.1		0.130	0.415	0.058	0.161	0.328	0.054	0.626	0.76	123 %	23.3%
1.34		0.206	5.489	0.072	0.158	0.671	0.073	0.626	0.76	77%	-23.3%
1.56		0.128	0.267	0.070	0.130	0.345	0.063	0.626	0.76	101 %	1.3%
2.18		0.917	2.176	0.433	0.886	1.734	0.377	0.626	0.76	97%	-3.4%
2.42		0.733	2.931	0.391	0.726	1.776	0.355	0.626	0.76	99%	-1.0%
2.5		0.089	0.215	0.050	0.092	0.172	0.043	0.626	0.76	103 %	3.1%
3.08		0.225	0.632	0.146	0.271	0.481	0.121	0.626	0.76	120 %	20.4%
3.42		0.169	0.459	0.086	0.178	0.434	0.076	0.626	0.76	105 %	5.4%
3.46		0.190	0.553	0.083	0.204	0.460	0.094	0.626	0.76	108 %	7.7%
3.48		0.187	0.510	0.073	0.174	0.473	0.080	0.626	0.76	93%	-6.9%
3.56		0.330	0.983	0.203	0.305	0.580	0.181	0.626	0.76	92%	-7.6%
7.14		0.050	0.134	0.033	0.056	0.096	0.027	0.626	0.76	112 %	11.6%
1.26		0.316	0.782	0.081	0.284	0.746	0.107	0.67	0.79	90%	-10.3%
1.58		0.124	0.242	0.067	0.118	0.277	0.060	0.67	0.79	95%	-4.8%
3.02		0.518	1.268	0.271	0.570	1.513	0.247	0.67	0.79	110 %	10.0%
7.36		0.101	0.251	0.063	0.105	0.162	0.054	0.67	0.79	103 %	3.4%
7.72		0.013	0.032	0.007	0.013	0.031	0.006	0.67	0.79	100 %	0.1%
8.44		0.025	0.058	0.015	0.023	0.045	0.008	0.67	0.79	95%	-5.5%
0.88		0.299	0.678	0.190	0.309	0.566	0.157	0.715	0.83	103 %	3.4%
1.12		0.048	0.127	0.026	0.050	0.094	0.025	0.715	0.83	106 %	5.6%
1.94		9.652	27.584	4.014	11.806	19.061	4.958	0.715	0.83	122 %	22.3%
3.38		0.146	0.515	0.098	0.149	0.342	0.088	0.715	0.83	103 %	2.6%
7.22		0.081	0.199	0.050	0.083	0.133	0.037	0.715	0.83	103 %	3.4%
7.78		0.025	0.052	0.013	0.022	0.090	0.011	0.715	0.83	88%	-11.9%
1.76		0.385	0.893	0.197	0.365	0.857	0.187	0.761	0.87	95%	-5.2%
7.28		0.058	0.143	0.034	0.062	0.094	0.025	0.761	0.87	108 %	8.5%
7.86		0.023	0.061	0.016	0.024	0.045	0.014	0.761	0.87	104 %	4.3%
1.14		0.052	0.143	0.032	0.057	0.206	0.031	0.808	0.89	110 %	10.0%
2.2		0.678	1.720	0.322	0.677	1.267	0.274	0.808	0.89	100 %	-0.2%
2.26		0.530	1.196	0.247	0.528	1.228	0.260	0.808	0.89	100 %	-0.3%
**2.48 - 2.44, 2.10**	Glutamine	0.146	0.364	0.074	0.143	0.375	0.070	0.808	0.89	97%	-2.6%
7.2		0.108	0.276	0.062	0.121	0.216	0.052	0.808	0.89	112 %	12.4%
7.42		0.047	0.101	0.021	0.048	0.082	0.021	0.808	0.89	102 %	2.1%
7.76		0.020	0.040	0.011	0.019	0.050	0.009	0.808	0.89	97%	-3.4%
7.8		0.041	0.087	0.019	0.039	0.120	0.017	0.808	0.89	94%	-6.0%
1.54		0.149	0.327	0.085	0.132	0.293	0.066	0.855	0.94	89%	-11.1%

3.4		0.079	0.243	0.043	0.086	0.206	0.045	0.855	0.94	108 %	8.4%
7.44		0.057	0.119	0.027	0.063	0.102	0.025	0.855	0.94	111 %	11.0%
7.74		0.015	0.037	0.008	0.017	0.036	0.007	0.903	0.99	113 %	12.5%
0.82		0.048	0.132	0.025	0.052	0.095	0.023	0.952	>0,999	107 %	6.9%
1.6		0.142	0.296	0.084	0.139	0.272	0.072	0.952	>0,999	98%	-1.6%
1.7		0.468	1.049	0.228	0.468	1.225	0.231	0.952	>0,999	100 %	-0.1%
2.22		0.510	1.180	0.268	0.505	0.846	0.234	0.952	>0,999	99%	-1.0%
0.84		0.089	0.203	0.047	0.087	0.172	0.042	>0,999	>0,999	98%	-2.4%
**0.86 - 0.88**	n(CH_3) LipoPTNs	0.156	0.370	0.093	0.161	0.298	0.086	>0,999	>0,999	103 %	2.9%
3.32		0.105	0.252	0.041	0.106	0.205	0.046	>0,999	>0,999	101 %	1.0%
5.22	Glucose	0.009	0.037	0.000	0.009	0.027	0.000	>0,999	>0,999	101 %	1.3%
7.3		0.071	0.178	0.042	0.075	0.123	0.033	>0,999	>0,999	106 %	5.8%
8.48		0.022	0.065	0.013	0.026	0.052	0.007	>0,999	>0,999	120 %	20.4%
**Trainees**											
**Chemical Shift (1H ppm)**	**Metabolite**	**Before**	**max**	**mim**	**After**	**max**	**mim**	**P value**	**q value**	**#**	**&**
**5.22***	**Glucose**	**0.009**	**0.022**	**0.002**	**0.013**	**0.032**	**0.005**	**0.02**	**>0,999**	**149 %**	**49%**
**8.46**	Formate	0.019	0.438	0.011	0.226	0.409	0.046	0.098	>0,999	1188 %	1088%
8.48		0.015	0.059	0.007	0.030	0.041	0.014	0.098	>0,999	194 %	94%
2.42		0.556	1.530	0.410	0.671	2.011	0.485	0.129	>0,999	121 %	21%
1.26		0.313	0.811	0.210	0.413	0.843	0.205	0.164	>0,999	132 %	32%
**7.08, 7.82 - 7.84**	Histidine	0.094	0.118	0.066	0.081	0.112	0.046	0.164	>0,999	86%	-14%
3.16		0.243	0.301	0.155	0.201	0.323	0.136	0.203	>0,999	82%	-18%
1.80		0.335	0.711	0.238	0.406	0.817	0.237	0.25	>0,999	121 %	21%
3.30		0.146	0.322	0.100	0.137	0.323	0.072	0.25	>0,999	94%	-6%
7.32		0.107	0.272	0.076	0.123	0.305	0.082	0.25	>0,999	115 %	15%
1.78		0.385	0.895	0.243	0.511	1.079	0.269	0.301	>0,999	133 %	33%
7.38		0.095	0.203	0.069	0.102	0.249	0.067	0.301	>0,999	108 %	8%
1.34		0.311	0.926	0.112	0.501	0.896	0.235	0.359	>0,999	161 %	61%
1.36		0.207	0.510	0.120	0.282	0.388	0.146	0.359	>0,999	136 %	36%
3.14		0.183	0.219	0.140	0.160	0.254	0.103	0.359	>0,999	88%	-12%
3.38		0.172	0.269	0.090	0.136	0.357	0.062	0.359	>0,999	79%	-21%
3.48		0.194	0.429	0.126	0.193	0.470	0.115	0.359	>0,999	99%	-1%
**3.58, 4.26, 1.34**	Threonine	0.907	1.959	0.458	0.704	1.949	0.307	0.359	>0,999	78%	-22%
7.40		0.079	0.162	0.058	0.085	0.179	0.045	0.359	>0,999	108 %	8%
8.06		0.070	0.091	0.042	0.062	0.089	0.030	0.359	>0,999	89%	-11%
1.22		0.293	0.661	0.242	0.341	0.673	0.251	0.426	>0,999	116 %	16%
1.24		0.179	0.365	0.137	0.200	0.379	0.119	0.426	>0,999	112 %	12%
1.58		0.200	0.378	0.113	0.158	0.489	0.095	0.426	>0,999	79%	-21%
1.60		0.203	0.373	0.127	0.169	0.457	0.109	0.426	>0,999	83%	-17%

1.76		0.351	0.666	0.258	0.428	0.773	0.239	0.426	>0,999	122 %	22%
1.94		11.962	29.805	10.236	14.683	33.334	7.465	0.426	>0,999	123 %	23%
3.08		0.226	0.538	0.170	0.300	0.667	0.136	0.426	>0,999	133 %	33%
3.46		0.248	0.523	0.154	0.248	0.549	0.138	0.426	>0,999	100 %	0%
5.28		0.009	0.016	0.002	0.011	0.024	0.002	0.426	>0,999	129 %	29%
1.12		0.061	0.113	0.039	0.061	0.109	0.032	0.496	>0,999	101 %	1%
1.30		0.104	0.204	0.074	0.107	0.164	0.056	0.496	>0,999	102 %	2%
2.16		0.736	1.168	0.550	0.794	1.401	0.421	0.496	>0,999	108 %	8%
3.32		0.117	0.253	0.078	0.131	0.294	0.065	0.496	>0,999	112 %	12%
3.34		0.132	0.235	0.063	0.098	0.368	0.043	0.496	>0,999	75%	-25%
3.36		0.150	0.268	0.071	0.108	0.431	0.042	0.496	>0,999	72%	-28%
3.60		0.389	0.654	0.274	0.441	0.535	0.174	0.496	>0,999	113 %	13%
3.62		0.481	0.944	0.366	0.545	0.786	0.226	0.496	>0,999	113 %	13%
3.70		0.397	0.710	0.275	0.415	0.586	0.182	0.496	>0,999	104 %	4%
3.72		0.411	0.582	0.304	0.399	0.544	0.214	0.496	>0,999	97%	-3%
3.84		0.562	1.096	0.459	0.601	0.980	0.272	0.496	>0,999	107 %	7%
3.92		0.402	0.796	0.284	0.383	0.656	0.175	0.496	>0,999	95%	-5%
5.82		0.034	0.179	0.004	0.051	0.190	0.011	0.496	>0,999	151 %	51%
**7.30**	Phenylalanine	0.078	0.159	0.053	0.085	0.183	0.053	0.496	>0,999	109 %	9%
8.08		0.072	0.097	0.046	0.069	0.096	0.033	0.496	>0,999	96%	-4%
8.44		0.020	0.059	0.010	0.030	0.038	0.014	0.496	>0,999	151 %	51%
0.80		0.044	0.077	0.032	0.042	0.084	0.025	0.57	>0,999	97%	-3%
1.10		0.188	0.391	0.121	0.185	0.400	0.083	0.57	>0,999	98%	-2%
1.14		0.064	0.119	0.039	0.067	0.112	0.043	0.57	>0,999	104 %	4%
1.54		0.212	0.364	0.118	0.189	0.456	0.112	0.57	>0,999	89%	-11%
1.56		0.233	0.446	0.123	0.189	0.583	0.109	0.57	>0,999	81%	-19%
2.22		0.587	1.039	0.468	0.629	1.008	0.408	0.57	>0,999	107 %	7%
**2.5, 2.53–2.54, 2.65, 2.68**	Citrate	0.119	0.201	0.075	0.107	0.251	0.066	0.57	>0,999	90%	-10%
3.06		0.455	0.995	0.298	0.594	1.211	0.243	0.57	>0,999	131 %	31%
3.18		0.124	0.193	0.101	0.119	0.198	0.070	0.57	>0,999	96%	-4%
3.42		0.265	0.385	0.158	0.223	0.580	0.117	0.57	>0,999	84%	-16%
3.90		0.384	0.814	0.268	0.381	0.686	0.173	0.57	>0,999	99%	-1%
**3.98**	Phosphoethanolamine	0.370	0.812	0.306	0.384	0.700	0.177	0.57	>0,999	104 %	4%
5.78		0.035	0.185	0.006	0.053	0.199	0.010	0.57	>0,999	151 %	51%
7.04		0.045	0.065	0.035	0.041	0.081	0.031	0.57	>0,999	91%	-9%
7.12		0.053	0.106	0.035	0.053	0.110	0.035	0.57	>0,999	101 %	1%
7.14		0.052	0.094	0.033	0.053	0.098	0.036	0.57	>0,999	103 %	3%
7.24		0.043	0.068	0.025	0.043	0.072	0.026	0.57	>0,999	100 %	0%
7.92		0.028	0.037	0.023	0.029	0.038	0.019	0.57	>0,999	103 %	3%

7.96		0.025	0.042	0.022	0.029	0.040	0.013	0.57	>0,999	114%	14%
1.28		0.177	0.337	0.122	0.192	0.352	0.104	0.652	>0,999	108%	8%
1.40		0.154	0.200	0.095	0.131	0.223	0.090	0.652	>0,999	85%	-15%
1.62		0.281	0.644	0.185	0.292	0.608	0.172	0.652	>0,999	104%	4%
1.86		0.215	0.452	0.173	0.206	0.371	0.117	0.652	>0,999	96%	-4%
1.88		0.256	0.472	0.194	0.228	0.395	0.139	0.652	>0,999	89%	-11%
1.92		1.720	3.551	1.375	2.085	3.787	1.297	0.652	>0,999	121%	21%
2.08		1.721	2.762	1.207	1.920	2.665	0.754	0.652	>0,999	112%	12%
2.18		1.239	2.018	0.837	1.165	2.315	0.762	0.652	>0,999	94%	-6%
2.38		0.771	1.697	0.610	0.827	1.286	0.443	0.652	>0,999	107%	7%
2.40		0.543	1.590	0.429	0.659	1.258	0.261	0.652	>0,999	122%	22%
**2.56**	Acetylcarnitine	0.083	0.139	0.072	0.098	0.173	0.045	0.652	>0,999	118%	18%
3.26		0.172	0.276	0.157	0.180	0.302	0.122	0.652	>0,999	105%	5%
3.40		0.112	0.210	0.060	0.081	0.301	0.039	0.652	>0,999	73%	-27%
3.54		0.302	0.406	0.217	0.253	0.450	0.163	0.652	>0,999	84%	-16%
3.56		0.343	0.554	0.256	0.338	0.512	0.187	0.652	>0,999	98%	-2%
3.68		0.534	1.016	0.351	0.553	0.834	0.234	0.652	>0,999	104%	4%
3.74		0.475	0.629	0.330	0.420	0.673	0.225	0.652	>0,999	89%	-11%
3.88		0.475	0.891	0.338	0.482	0.769	0.225	0.652	>0,999	101%	1%
3.94		0.620	0.723	0.436	0.524	0.788	0.284	0.652	>0,999	85%	-15%
3.96		0.446	0.791	0.295	0.489	0.657	0.218	0.652	>0,999	110%	10%
4.06		0.121	0.230	0.089	0.124	0.214	0.065	0.652	>0,999	102%	2%
4.16		0.088	0.126	0.066	0.105	0.136	0.062	0.652	>0,999	119%	19%
5.80		0.039	0.207	0.006	0.059	0.222	0.012	0.652	>0,999	150%	50%
5.84		0.028	0.133	0.005	0.038	0.137	0.014	0.652	>0,999	138%	38%
5.88		0.017	0.067	0.004	0.021	0.068	0.009	0.652	>0,999	122%	22%
7.26		0.064	0.128	0.049	0.067	0.142	0.047	0.652	>0,999	104%	4%
7.34		0.149	0.303	0.108	0.137	0.329	0.115	0.652	>0,999	92%	-8%
7.42		0.048	0.090	0.035	0.044	0.108	0.030	0.652	>0,999	91%	-9%
7.76		0.021	0.027	0.013	0.018	0.034	0.013	0.652	>0,999	87%	-13%
7.78		0.025	0.041	0.017	0.020	0.048	0.013	0.652	>0,999	80%	-20%
7.90		0.022	0.034	0.015	0.021	0.034	0.011	0.652	>0,999	96%	-4%
7.94		0.024	0.037	0.021	0.026	0.035	0.012	0.652	>0,999	104%	4%
8.00		0.034	0.061	0.022	0.040	0.053	0.014	0.652	>0,999	116%	16%
8.04		0.048	0.070	0.028	0.051	0.061	0.020	0.652	>0,999	106%	6%
8.22		0.026	0.054	0.018	0.026	0.051	0.012	0.652	>0,999	99%	-1%
8.98		0.002	0.006	0.001	0.003	0.007	0.000	0.652	>0,999	112%	12%
**0.86 - 0.88**	n(CH_3) LipoPTNs	0.178	0.277	0.116	0.181	0.382	0.117	0.734	>0,999	102%	2%
1.06		1.150	2.564	0.776	1.296	2.796	0.461	0.734	>0,999	113%	13%
1.38		0.147	0.230	0.098	0.144	0.220	0.086	0.734	>0,999	98%	-2%

1.64		0.484	1.286	0.308	0.535	1.235	0.270	0.734	>0,999	111%	11%
**1.90**	Acetate	0.352	0.691	0.242	0.340	0.545	0.202	0.734	>0,999	97%	-3%
**2.00**	N-Acetyl of Glycoproteins	0.764	1.529	0.518	0.865	1.267	0.321	0.734	>0,999	113%	13%
2.04		1.184	2.987	0.825	1.441	2.394	0.507	0.734	>0,999	122%	22%
2.06		2.004	3.839	1.401	2.242	3.363	0.877	0.734	>0,999	112%	12%
2.10		0.896	1.564	0.579	0.989	1.400	0.369	0.734	>0,999	110%	10%
2.12		0.641	1.212	0.476	0.734	1.040	0.293	0.734	>0,999	115%	15%
2.14		0.645	1.150	0.484	0.726	1.054	0.301	0.734	>0,999	113%	13%
2.20		0.819	1.454	0.588	0.844	1.507	0.579	0.734	>0,999	103%	3%
**2.48 - 2.44, 2.10**	Glutamine	0.216	0.283	0.120	0.165	0.356	0.075	0.734	>0,999	77%	-23%
2.52		0.080	0.143	0.064	0.087	0.185	0.050	0.734	>0,999	109%	9%
**2.54**	Citrate	0.074	0.121	0.062	0.091	0.150	0.037	0.734	>0,999	122%	22%
2.58		0.080	0.134	0.068	0.099	0.164	0.042	0.734	>0,999	123%	23%
**2.66**	R-Malic acid	0.088	0.158	0.077	0.085	0.175	0.050	0.734	>0,999	96%	-4%
2.68		0.089	0.153	0.074	0.090	0.157	0.049	0.734	>0,999	102%	2%
2.70		0.100	0.199	0.086	0.111	0.187	0.052	0.734	>0,999	111%	11%
2.72		0.120	0.269	0.108	0.128	0.230	0.062	0.734	>0,999	106%	6%
2.80		0.111	0.207	0.089	0.118	0.193	0.051	0.734	>0,999	107%	7%
2.96		0.078	0.134	0.055	0.083	0.157	0.052	0.734	>0,999	107%	7%
2.98		0.118	0.225	0.085	0.125	0.245	0.079	0.734	>0,999	107%	7%
**3.20 - 3.22**	$n(CH_3)_3$ Choline	0.140	0.268	0.130	0.145	0.269	0.088	0.734	>0,999	103%	3%
3.44		0.309	0.490	0.233	0.294	0.670	0.173	0.734	>0,999	95%	-5%
3.86		0.574	1.049	0.458	0.647	0.965	0.298	0.734	>0,999	113%	13%
4.00		0.390	0.983	0.329	0.430	0.816	0.202	0.734	>0,999	110%	10%
4.04		0.158	0.330	0.125	0.177	0.307	0.078	0.734	>0,999	112%	12%
4.08		0.107	0.165	0.080	0.106	0.153	0.064	0.734	>0,999	99%	-1%
6.66		0.014	0.027	0.008	0.015	0.026	0.007	0.734	>0,999	112%	12%
6.90		0.177	0.495	0.145	0.199	0.421	0.091	0.734	>0,999	112%	12%
6.92		0.174	0.415	0.142	0.194	0.386	0.092	0.734	>0,999	111%	11%
7.28		0.065	0.118	0.042	0.063	0.159	0.045	0.734	>0,999	98%	-2%
7.54		0.052	0.146	0.041	0.063	0.110	0.024	0.734	>0,999	121%	21%
7.56		0.062	0.239	0.049	0.076	0.166	0.029	0.734	>0,999	121%	21%
7.58		0.063	0.249	0.050	0.078	0.180	0.028	0.734	>0,999	124%	24%
7.70		0.015	0.024	0.011	0.015	0.021	0.006	0.734	>0,999	102%	2%
7.98		0.029	0.053	0.019	0.031	0.048	0.013	0.734	>0,999	106%	6%
8.96		0.004	0.013	0.002	0.003	0.012	0.001	0.734	>0,999	72%	-28%
0.84		0.102	0.157	0.059	0.089	0.168	0.062	0.82	>0,999	87%	-13%
0.92		0.492	0.959	0.297	0.479	1.514	0.273	0.82	>0,999	97%	-3%

1.44		0.165	0.351	0.135	0.170	0.385	0.118	0.82	>0,999	103 %	3%
1.50		0.384	0.513	0.239	0.329	0.635	0.206	0.82	>0,999	86%	-14%
1.52		0.210	0.334	0.134	0.207	0.436	0.128	0.82	>0,999	98%	-2%
1.66		0.723	2.063	0.452	0.829	2.004	0.397	0.82	>0,999	115 %	15%
1.84		0.211	0.480	0.187	0.241	0.411	0.126	0.82	>0,999	114 %	14%
1.98		0.719	1.451	0.505	0.791	1.248	0.320	0.82	>0,999	110 %	10%
2.02		0.975	2.081	0.628	1.086	1.721	0.380	0.82	>0,999	111 %	11%
2.24		0.873	2.264	0.644	1.131	2.246	0.504	0.82	>0,999	130 %	30%
2.46		0.293	0.582	0.182	0.357	0.546	0.097	0.82	>0,999	122 %	22%
2.74		0.124	0.253	0.109	0.134	0.230	0.068	0.82	>0,999	108 %	8%
2.78		0.095	0.208	0.085	0.102	0.184	0.046	0.82	>0,999	106 %	6%
2.84		0.083	0.164	0.073	0.085	0.158	0.040	0.82	>0,999	102 %	2%
2.86		0.080	0.158	0.067	0.087	0.154	0.041	0.82	>0,999	109 %	9%
2.92		0.065	0.124	0.045	0.069	0.161	0.050	0.82	>0,999	107 %	7%
3.00		0.226	0.517	0.176	0.262	0.526	0.137	0.82	>0,999	116 %	16%
3.12		0.116	0.181	0.096	0.109	0.191	0.069	0.82	>0,999	94%	-6%
3.24		0.213	0.489	0.133	0.226	0.399	0.138	0.82	>0,999	106 %	6%
**3.28**	L-Cysteic acid	0.289	0.470	0.205	0.226	0.469	0.180	0.82	>0,999	78%	-22%
3.50		0.174	0.326	0.128	0.174	0.371	0.115	0.82	>0,999	100 %	0%
3.64		0.601	1.138	0.426	0.603	0.998	0.313	0.82	>0,999	100 %	0%
3.78		0.859	1.197	0.570	0.738	1.521	0.435	0.82	>0,999	86%	-14%
3.82		0.631	1.028	0.435	0.610	0.904	0.312	0.82	>0,999	97%	-3%
**4.12 - 4.10, 1.32**	Lactate	0.097	0.130	0.068	0.097	0.183	0.059	0.82	>0,999	100 %	0%
4.14		0.086	0.113	0.062	0.094	0.144	0.058	0.82	>0,999	109 %	9%
6.60		0.009	0.019	0.005	0.011	0.020	0.004	0.82	>0,999	117 %	17%
6.72		0.016	0.030	0.010	0.017	0.027	0.007	0.82	>0,999	110 %	10%
6.74		0.019	0.036	0.013	0.019	0.037	0.008	0.82	>0,999	101 %	1%
6.86		0.156	0.251	0.121	0.178	0.236	0.086	0.82	>0,999	114 %	14%
**6.88, 7.18 - 7.20**	Tyrosine	0.166	0.359	0.122	0.189	0.297	0.079	0.82	>0,999	114 %	14%
7.16		0.061	0.115	0.038	0.067	0.119	0.037	0.82	>0,999	108 %	8%
7.52		0.034	0.075	0.028	0.040	0.065	0.019	0.82	>0,999	117 %	17%
7.60		0.050	0.212	0.041	0.065	0.158	0.022	0.82	>0,999	130 %	30%
7.62		0.035	0.107	0.027	0.039	0.082	0.014	0.82	>0,999	111 %	11%
7.64		0.031	0.070	0.024	0.035	0.059	0.015	0.82	>0,999	114 %	14%
7.66		0.026	0.057	0.022	0.029	0.048	0.012	0.82	>0,999	110 %	10%
7.88		0.024	0.030	0.017	0.022	0.030	0.014	0.82	>0,999	93%	-7%
8.20		0.026	0.046	0.015	0.025	0.046	0.011	0.82	>0,999	94%	-6%
8.42		0.026	0.078	0.015	0.028	0.051	0.013	0.82	>0,999	106 %	6%
0.82		0.058	0.095	0.037	0.054	0.099	0.035	0.91	>0,999	92%	-8%

0.88		0.340	0.744	0.222	0.382	1.156	0.250	0.91	>0,999	112 %	12%
0.90		0.645	1.343	0.391	0.591	2.224	0.371	0.91	>0,999	92%	-8%
**1.04**	Isobutyrate	0.327	0.581	0.212	0.351	0.619	0.121	0.91	>0,999	107 %	7%
1.08		1.118	2.356	0.695	1.129	2.631	0.441	0.91	>0,999	101 %	1%
1.42		0.147	0.323	0.110	0.144	0.344	0.102	0.91	>0,999	98%	-2%
**1.48 - 1.46**	Alanine	0.390	0.520	0.226	0.328	0.669	0.208	0.91	>0,999	84%	-16%
1.70		0.462	1.032	0.367	0.525	1.062	0.272	0.91	>0,999	114 %	14%
1.74		0.407	0.790	0.293	0.447	0.902	0.234	0.91	>0,999	110 %	10%
1.82		0.203	0.444	0.156	0.254	0.416	0.130	0.91	>0,999	125 %	25%
2.26		0.604	1.440	0.455	0.712	1.437	0.324	0.91	>0,999	118 %	18%
2.30		0.453	1.014	0.355	0.490	0.882	0.231	0.91	>0,999	108 %	8%
2.32		0.582	1.288	0.412	0.602	1.096	0.276	0.91	>0,999	104 %	4%
**2.34**	Glutamate	0.758	1.517	0.539	0.769	1.319	0.368	0.91	>0,999	101 %	1%
**2.64 - 2.66**	R-Malic acid	0.073	0.122	0.057	0.069	0.143	0.042	0.91	>0,999	94%	-6%
2.82		0.105	0.195	0.085	0.110	0.192	0.047	0.91	>0,999	105 %	5%
2.90		0.097	0.246	0.072	0.102	0.363	0.080	0.91	>0,999	105 %	5%
2.94		0.066	0.111	0.044	0.070	0.125	0.041	0.91	>0,999	106 %	6%
3.02		0.586	1.387	0.418	0.688	1.378	0.339	0.91	>0,999	117 %	17%
3.04		0.754	1.889	0.508	0.912	2.005	0.408	0.91	>0,999	121 %	21%
3.10		0.119	0.208	0.093	0.125	0.236	0.071	0.91	>0,999	105 %	5%
3.52		0.244	0.408	0.184	0.232	0.478	0.143	0.91	>0,999	95%	-5%
3.66		0.666	1.246	0.445	0.670	1.076	0.338	0.91	>0,999	101 %	1%
3.76		0.792	1.210	0.542	0.735	1.425	0.459	0.91	>0,999	93%	-7%
3.80		0.719	0.880	0.429	0.619	1.007	0.304	0.91	>0,999	86%	-14%
4.02		0.249	0.571	0.203	0.305	0.496	0.124	0.91	>0,999	122 %	22%
5.62		0.011	0.034	0.009	0.017	0.034	0.005	0.91	>0,999	154 %	54%
5.64		0.011	0.034	0.006	0.017	0.035	0.005	0.91	>0,999	157 %	57%
6.62		0.011	0.025	0.007	0.013	0.024	0.006	0.91	>0,999	116 %	16%
6.68		0.016	0.030	0.009	0.018	0.029	0.007	0.91	>0,999	115 %	15%
6.70		0.015	0.029	0.009	0.017	0.027	0.006	0.91	>0,999	111 %	11%
6.76		0.021	0.043	0.016	0.024	0.044	0.011	0.91	>0,999	113 %	13%
6.78		0.025	0.050	0.019	0.026	0.043	0.013	0.91	>0,999	108 %	8%
6.82		0.057	0.126	0.047	0.059	0.125	0.037	0.91	>0,999	102 %	2%
6.84		0.093	0.174	0.078	0.101	0.178	0.061	0.91	>0,999	109 %	9%
6.94		0.078	0.211	0.066	0.103	0.201	0.048	0.91	>0,999	132 %	32%
7.00		0.049	0.107	0.038	0.051	0.105	0.031	0.91	>0,999	104 %	4%
7.06		0.043	0.098	0.029	0.043	0.080	0.024	0.91	>0,999	100 %	0%
7.36		0.103	0.182	0.083	0.111	0.231	0.080	0.91	>0,999	107 %	7%

7.44		0.063	0.109	0.044	0.052	0.136	0.034	0.91	>0,999	82%	-18%
7.48		0.019	0.037	0.014	0.021	0.029	0.007	0.91	>0,999	110 %	10%
7.50		0.026	0.052	0.019	0.031	0.043	0.012	0.91	>0,999	119 %	19%
7.72		0.015	0.020	0.010	0.014	0.022	0.008	0.91	>0,999	96%	-4%
7.74		0.018	0.026	0.012	0.017	0.027	0.010	0.91	>0,999	94%	-6%
7.80		0.039	0.066	0.024	0.034	0.087	0.019	0.91	>0,999	89%	-11%
7.86		0.025	0.056	0.016	0.020	0.055	0.011	0.91	>0,999	82%	-18%
8.02		0.035	0.073	0.025	0.041	0.065	0.017	0.91	>0,999	115 %	15%
8.12		0.030	0.046	0.023	0.035	0.049	0.017	0.91	>0,999	117 %	17%
8.14		0.023	0.037	0.015	0.024	0.039	0.012	0.91	>0,999	106 %	6%
8.16		0.026	0.039	0.017	0.026	0.039	0.012	0.91	>0,999	103 %	3%
8.26		0.016	0.040	0.013	0.018	0.033	0.010	0.91	>0,999	111 %	11%
8.28		0.016	0.040	0.011	0.020	0.032	0.009	0.91	>0,999	121 %	21%
8.30		0.019	0.054	0.014	0.023	0.039	0.010	0.91	>0,999	119 %	19%
8.34		0.020	0.053	0.014	0.022	0.036	0.008	0.91	>0,999	107 %	7%
8.38		0.019	0.053	0.013	0.021	0.038	0.008	0.91	>0,999	107 %	7%
8.40		0.024	0.071	0.016	0.026	0.047	0.011	0.91	>0,999	111 %	11%
**0.94**	Leucine	0.529	0.824	0.350	0.479	1.185	0.324	>0,999	>0,999	91%	-9%
0.96		0.516	0.702	0.389	0.490	0.936	0.351	>0,999	>0,999	95%	-5%
0.98 - 1.03	L-Valine	0.385	0.576	0.249	0.294	0.712	0.246	>0,999	>0,999	76%	-24%
1.00		0.224	0.391	0.138	0.217	0.433	0.120	>0,999	>0,999	97%	-3%
1.02		0.214	0.361	0.129	0.223	0.412	0.102	>0,999	>0,999	104 %	4%
1.68		0.666	1.741	0.437	0.734	1.710	0.359	>0,999	>0,999	110 %	10%
1.72		0.440	0.936	0.322	0.447	1.014	0.237	>0,999	>0,999	101 %	1%
1.96		0.881	1.732	0.672	0.988	1.599	0.437	>0,999	>0,999	112 %	12%
2.28		0.373	0.762	0.310	0.430	0.706	0.197	>0,999	>0,999	115 %	15%
2.36		0.768	1.452	0.520	0.795	1.202	0.335	>0,999	>0,999	104 %	4%
2.44		0.372	1.219	0.283	0.465	1.041	0.147	>0,999	>0,999	125 %	25%
2.60		0.077	0.128	0.067	0.092	0.136	0.042	>0,999	>0,999	120 %	20%
2.62		0.101	0.141	0.075	0.097	0.178	0.061	>0,999	>0,999	96%	-4%
2.76		0.127	0.255	0.101	0.130	0.224	0.064	>0,999	>0,999	102 %	2%
2.88		0.067	0.124	0.057	0.073	0.168	0.046	>0,999	>0,999	108 %	8%
6.64		0.012	0.024	0.007	0.014	0.023	0.005	>0,999	>0,999	111 %	11%
6.80		0.032	0.069	0.026	0.033	0.065	0.021	>0,999	>0,999	102 %	2%
6.96		0.046	0.131	0.040	0.060	0.123	0.027	>0,999	>0,999	132 %	32%
6.98		0.033	0.080	0.026	0.037	0.073	0.018	>0,999	>0,999	111 %	11%
7.02		0.040	0.076	0.030	0.042	0.080	0.028	>0,999	>0,999	106 %	6%
7.10		0.044	0.072	0.030	0.044	0.076	0.027	>0,999	>0,999	102 %	2%
7.20		0.119	0.229	0.092	0.134	0.249	0.073	>0,999	>0,999	113 %	13%

7.22		0.081	0.144	0.068	0.073	0.168	0.056	>0,999	>0,999	89%	-11%
7.46		0.026	0.039	0.020	0.024	0.049	0.013	>0,999	>0,999	92%	-8%
7.68		0.017	0.033	0.013	0.018	0.029	0.008	>0,999	>0,999	111 %	11%
7.84		0.036	0.117	0.025	0.037	0.082	0.020	>0,999	>0,999	103 %	3%
8.10		0.042	0.062	0.035	0.054	0.063	0.021	>0,999	>0,999	126 %	26%
8.18		0.019	0.040	0.015	0.021	0.032	0.009	>0,999	>0,999	115 %	15%
8.24		0.019	0.048	0.013	0.022	0.038	0.009	>0,999	>0,999	112 %	12%
8.32		0.021	0.059	0.015	0.024	0.039	0.010	>0,999	>0,999	114 %	14%
8.36		0.020	0.052	0.014	0.021	0.038	0.009	>0,999	>0,999	105 %	5%

Table.S4 Table of chemical shifts used for relative quantification of urine metabolites from Instructors and Trainees. Paired analysis, carried out with the Wilcoxon test nonparametric, data expressed as median, minimum and maximum after Shapiro-Wilk normality test. # corresponds to instructors over trainees. & corresponds to the % from #. *Values less than p < 0.05.

Instructors											
**Chemical Shift (1H ppm)**	**Metabolite**	**Before**	**max**	**mim**	**After**	**max**	**mim**	**P value**	**q value**	**#**	**&**
**8.84* - 8.86**	**Trigonelline**	**0.130**	**1.010**	**0.010**	**0.200**	**1.480**	**0.010**	**0.001**	**0.28**	**154%**	**53.8%**
**9.14***		**0.110**	**0.830**	**0.010**	**0.180**	**1.160**	**0.000**	**0.003**	**0.36**	**164%**	**63.6%**
**8.10***		**0.080**	**0.490**	**0.000**	**0.120**	**0.830**	**0.000**	**0.005**	**0.39**	**150%**	**50.0%**
**2.7*, 7.40-7.36, 2.66**	**Atropine**	**0.460**	**1.710**	**0.090**	**0.570**	**3.840**	**0.120**	**0.02**	**0.84**	**124%**	**23.9%**
**2.58, 2.72**	Nicotine	0.710	2.300	0.040	0.740	5.710	0.060	0.05	>0.99	104%	4.2%
9.3		0.020	0.050	0.000	0.010	0.020	0.000	0.06	>0.99	50%	-50.0%
7.96		0.150	0.870	0.000	0.140	1.440	0.010	0.10	>0.99	93%	-6.7%
1.98		0.530	1.070	0.010	0.440	1.370	0.010	0.21	>0.99	83%	-17.0%
2.56		1.710	4.950	0.240	1.190	4.450	0.500	0.21	>0.99	70%	-30.4%
**2.76, 7.07, 8.85, 8.79**	NADPH	0.340	0.580	0.000	0.300	0.800	0.020	0.24	>0.99	88%	-11.8%
**7.68 7.18 - 7.20**	Tyrosine	0.220	0.510	0.010	0.220	0.550	0.020	0.24	>0.99	100%	0.0%
2.98		0.270	0.570	0.010	0.280	0.690	0.020	0.25	>0.99	104%	3.7%
2.78		0.300	0.540	0.010	0.310	0.690	0.030	0.26	>0.99	103%	3.3%
2.06		1.410	2.480	0.150	1.200	3.230	0.180	0.27	>0.99	85%	-14.9%
2.8		0.420	0.640	0.020	0.360	0.900	0.050	0.27	>0.99	86%	-14.3%
2.08		1.130	1.960	0.090	0.910	2.390	0.110	0.28	>0.99	81%	-19.5%
2.82		0.310	0.510	0.010	0.260	0.740	0.010	0.28	>0.99	84%	-16.1%
**2.04 - 2.01. 1.86**	N_Acetyl_L_glutamic_acid	1.100	2.080	0.140	0.910	2.150	0.120	0.29	>0.99	83%	-17.3%
2.22		0.720	1.230	0.080	0.560	1.450	0.010	0.29	>0.99	78%	-22.2%
2.64		0.450	0.770	0.020	0.340	0.960	0.030	0.29	>0.99	76%	-24.4%
3.3		1.640	3.550	0.160	1.410	4.010	0.050	0.29	>0.99	86%	-14.0%
8.2		0.040	0.120	0.000	0.030	0.400	0.000	0.29	>0.99	75%	-25.0%
8.56		0.040	0.220	0.000	0.030	0.140	0.000	0.30	>0.99	75%	-25.0%
1.96		0.660	1.280	0.030	0.560	1.580	0.040	0.31	>0.99	85%	-15.2%
**1.9**	Acetate	0.520	0.980	0.000	0.490	1.320	0.010	0.32	>0.99	94%	-5.8%
2.24		0.660	1.100	0.070	0.500	1.360	0.030	0.32	>0.99	76%	-24.2%
3.72		2.650	6.030	0.430	3.090	7.120	0.360	0.32	>0.99	117%	16.6%
6.66		0.090	0.200	0.000	0.050	0.340	0.000	0.32	>0.99	56%	-44.4%
1.84		0.520	1.010	0.000	0.440	1.190	0.010	0.33	>0.99	85%	-15.4%
2		0.800	1.640	0.060	0.670	1.960	0.040	0.33	>0.99	84%	-16.3%
3.56		1.130	2.000	0.270	0.860	2.260	0.090	0.33	>0.99	76%	-23.9%
3.62		1.530	2.550	0.330	1.200	2.980	0.200	0.33	>0.99	78%	-21.6%
2.9		0.370	0.800	0.020	0.320	1.000	0.020	0.34	>0.99	86%	-13.5%
3.82		1.660	3.000	0.280	1.330	4.070	0.210	0.34	>0.99	80%	-19.9%
2.62		0.470	0.910	0.020	0.330	1.130	0.020	0.35	>0.99	70%	-29.8%
2.88		0.390	0.950	0.030	0.370	1.080	0.040	0.35	>0.99	95%	-5.1%
3.36		0.880	1.510	0.150	0.700	2.050	0.110	0.35	>0.99	80%	-20.5%
3.9		1.270	2.270	0.240	1.050	3.050	0.170	0.35	>0.99	83%	-17.3%

2.42		0.610	1.040	0.030	0.500	1.180	0.020	0.36	>0.99	82%	-18.0%
3.32		1.290	2.280	0.270	0.960	3.270	0.110	0.36	>0.99	74%	-25.6%
**3.96**	3_Methylhistidine	2.150	3.220	0.370	1.590	4.100	0.370	0.36	>0.99	74%	-26.0%
1.94		0.890	1.610	0.070	0.710	1.970	0.120	0.37	>0.99	80%	-20.2%
2.02		0.600	1.250	0.010	0.460	1.190	0.010	0.37	>0.99	77%	-23.3%
2.1		0.840	1.550	0.030	0.660	1.780	0.090	0.37	>0.99	79%	-21.4%
2.26		0.930	1.810	0.150	0.620	2.190	0.140	0.37	>0.99	67%	-33.3%
2.32		0.560	0.960	0.030	0.430	1.010	0.010	0.37	>0.99	77%	-23.2%
2.92		0.490	0.870	0.040	0.350	1.120	0.060	0.37	>0.99	71%	-28.6%
3.88		1.310	2.410	0.250	1.110	2.750	0.170	0.37	>0.99	85%	-15.3%
**8.46**	Formate	0.030	0.080	0.010	0.030	0.130	0.010	0.37	>0.99	100%	0.0%
8.52		0.080	0.560	0.010	0.050	0.770	0.010	0.37	>0.99	63%	-37.5%
2.2		0.790	1.360	0.100	0.580	1.660	0.050	0.38	>0.99	73%	-26.6%
2.96		0.250	0.510	0.000	0.230	0.660	0.010	0.38	>0.99	92%	-8.0%
2.38		0.640	1.310	0.060	0.620	1.380	0.030	0.39	>0.99	97%	-3.1%
3.7		2.850	4.960	0.820	2.400	5.370	0.740	0.39	>0.99	84%	-15.8%
3.16		1.310	2.540	0.240	1.330	2.540	0.390	0.40	>0.99	102%	1.5%
3.8		2.280	4.350	0.390	1.830	8.330	0.390	0.41	>0.99	80%	-19.7%
3.6		1.420	2.400	0.340	1.110	2.630	0.150	0.42	>0.99	78%	-21.8%
1.2		0.610	1.390	0.080	0.570	1.320	0.150	0.43	>0.99	93%	-6.6%
1.92		0.710	1.310	0.040	0.640	1.630	0.050	0.43	>0.99	90%	-9.9%
2.5		0.680	1.180	0.060	0.520	1.580	0.030	0.43	>0.99	76%	-23.5%
3.34		0.760	1.310	0.090	0.560	2.190	0.060	0.43	>0.99	74%	-26.3%
3.64		2.000	3.680	0.470	1.580	4.090	0.340	0.43	>0.99	79%	-21.0%
3.78		2.440	4.220	0.450	1.930	5.810	0.470	0.43	>0.99	79%	-20.9%
1.82		0.510	1.140	0.010	0.410	1.100	0.010	0.44	>0.99	80%	-19.6%
2.3		0.710	1.420	0.020	0.570	1.630	0.050	0.45	>0.99	80%	-19.7%
3.28		4.410	12.850	0.660	3.840	14.91	0.820	0.45	>0.99	87%	-12.9%
2.18		0.930	1.650	0.110	0.620	1.640	0.050	0.46	>0.99	67%	-33.3%
2.84		0.490	0.790	0.040	0.450	1.110	0.080	0.46	>0.99	92%	-8.2%
**3.22**	n$(CH_3)_3$ Choline	1.480	2.670	0.230	1.180	4.210	0.220	0.46	>0.99	80%	-20.3%
2.34		0.580	1.110	0.020	0.460	1.040	0.010	0.47	>0.99	79%	-20.7%
3.84		1.890	3.970	0.430	1.570	4.350	0.360	0.47	>0.99	83%	-16.9%
8.34		0.080	0.260	0.010	0.080	0.390	0.010	0.48	>0.99	100%	0.0%
2.12		0.730	1.410	0.010	0.550	1.460	0.070	0.49	>0.99	75%	-24.7%
3.46		1.380	3.160	0.210	1.170	2.750	0.110	0.49	>0.99	85%	-15.2%
3		0.400	0.910	0.020	0.320	0.860	0.040	0.50	>0.99	80%	-20.0%
7.72		0.150	0.420	0.010	0.070	0.570	0.020	0.50	>0.99	47%	-53.3%
3.12		0.970	2.080	0.140	0.910	2.030	0.150	0.51	>0.99	94%	-6.2%
3.5		0.710	1.750	0.130	0.580	1.730	0.090	0.51	>0.99	82%	-18.3%
**3.58, 4.26, 1.34**	Threonine	2.230	3.870	0.530	1.860	7.220	0.510	0.51	>0.99	83%	-16.6%
**2.54 - 2.53, 2.5, 2.65, 2.68**	Citrate	1.090	2.840	0.250	0.990	3.430	0.340	0.52	>0.99	91%	-9.2%
2.74		1.190	1.990	0.170	0.990	2.480	0.280	0.52	>0.99	83%	-16.8%
**3.42**	L_Carnitine	0.940	2.420	0.100	0.880	2.530	0.070	0.52	>0.99	94%	-6.4%
2.48		0.850	1.420	0.100	0.650	1.520	0.060	0.54	>0.99	76%	-23.5%
3.06		25.90	48.31	4.37	24.10	69.44	7.06	0.54	>0.99	93%	-6.9%

3.54		1.140	2.630	0.250	0.860	2.450	0.120	0.54	>0.99	75%	-24.6%
3.68		2.760	5.220	0.530	2.030	5.700	0.640	0.54	>0.99	74%	-26.4%
7.06		0.100	0.940	0.000	0.150	0.350	0.000	0.54	>0.99	150%	50.0%
3.86		1.510	2.900	0.310	1.400	3.410	0.240	0.56	>0.99	93%	-7.3%
7.66		0.510	2.780	0.060	0.360	2.370	0.050	0.56	>0.99	71%	-29.4%
2.28		1.040	2.190	0.070	0.680	2.860	0.170	0.58	>0.99	65%	-34.6%
2.94		0.470	1.100	0.070	0.360	1.970	0.060	0.58	>0.99	77%	-23.4%
5.78		13.500	32.330	1.950	13.900	27.43	3.600	0.59	>0.99	103%	3.0%
1.64		0.390	0.960	0.010	0.330	0.960	0.020	0.60	>0.99	85%	-15.4%
1.68		0.520	1.130	0.020	0.410	1.130	0.030	0.60	>0.99	79%	-21.2%
7.18		0.230	0.640	0.020	0.200	0.630	0.030	0.60	>0.99	87%	-13.0%
7.42		0.490	1.240	0.080	0.440	2.070	0.110	0.60	>0.99	90%	-10.2%
**0.94, 1.00**	L_Isoleucine	0.420	0.870	0.030	0.370	0.940	0.040	0.61	>0.99	88%	-11.9%
0.96		0.330	0.680	0.020	0.290	0.760	0.030	0.61	>0.99	88%	-12.1%
1.66		0.470	1.040	0.010	0.390	1.040	0.020	0.61	>0.99	83%	-17.0%
3.40		0.390	1.070	0.030	0.350	0.910	0.000	0.61	>0.99	90%	-10.3%
4.02		0.860	1.720	0.110	0.690	2.090	0.060	0.62	>0.99	80%	-19.8%
**4.04, 3.03 - 3.04**	Creatinine	1.000	1.860	0.110	0.960	2.310	0.140	0.62	>0.99	96%	-4.0%
**2.44, 2.14, 3.76**	L_Glutamine	0.730	1.300	0.050	0.590	1.260	0.050	0.63	>0.99	81%	-19.2%
3.38		0.510	0.890	0.100	0.440	0.980	0.070	0.63	>0.99	86%	-13.7%
3.92		1.240	2.810	0.220	0.900	2.900	0.160	0.63	>0.99	73%	-27.4%
**7.08, 2.68**	L_Anserine	0.220	1.060	0.020	0.230	1.780	0.070	0.63	>0.99	105%	4.5%
7.76		0.080	0.200	0.000	0.050	0.390	0.010	0.64	>0.99	63%	-37.5%
1.22		0.640	1.340	0.070	0.480	1.210	0.120	0.65	>0.99	75%	-25.0%
1.26		0.600	1.650	0.040	0.490	1.440	0.070	0.65	>0.99	82%	-18.3%
3.26, 3.66		1.570	3.510	0.160	1.490	5.420	0.200	0.65	>0.99	95%	-5.1%
3.48		0.600	1.330	0.090	0.450	1.470	0.010	0.65	>0.99	75%	-25.0%
2.46		0.970	2.000	0.090	0.770	1.980	0.110	0.66	>0.99	79%	-20.6%
6.98		0.110	0.420	0.020	0.110	0.560	0.010	0.67	>0.99	100%	0.0%
7.36		0.650	2.080	0.070	0.510	2.200	0.090	0.67	>0.99	78%	-21.5%
7.60		0.080	0.480	0.000	0.050	0.540	0.000	0.67	>0.99	63%	-37.5%
4.10		0.380	1.160	0.040	0.360	0.870	0.020	0.68	>0.99	95%	-5.3%
8.22		0.040	0.080	0.000	0.030	0.170	0.000	0.68	>0.99	75%	-25.0%
3.10		0.550	1.380	0.030	0.440	1.320	0.050	0.69	>0.99	80%	-20.0%
3.18		1.040	2.230	0.080	0.970	3.140	0.140	0.69	>0.99	93%	-6.7%
3.74		2.030	6.620	0.400	1.710	10.87	0.300	0.69	>0.99	84%	-15.8%
6.86		0.110	0.500	0.020	0.110	0.600	0.010	0.69	>0.99	100%	0.0%
7.44		0.560	1.310	0.120	0.470	1.760	0.160	0.69	>0.99	84%	-16.1%
1.34		0.710	1.910	0.100	0.700	1.810	0.120	0.70	>0.99	99%	-1.4%
3.24		2.420	7.730	0.330	2.130	6.210	0.290	0.70	>0.99	88%	-12.0%
1.10		0.360	0.840	0.020	0.310	0.800	0.050	0.71	>0.99	86%	-13.9%
6.96		0.070	0.220	0.000	0.050	0.310	0.000	0.72	>0.99	71%	-28.6%
4.00		1.170	2.400	0.170	0.940	3.220	0.150	0.73	>0.99	80%	-19.7%
5.8		12.910	31.620	1.790	13.110	26.89	3.340	0.73	>0.99	102%	1.5%
7.38		1.090	2.450	0.150	0.830	3.670	0.180	0.73	>0.99	76%	-23.9%
**1.48 - 1.46**	Alanine	0.550	1.760	0.060	0.550	1.700	0.070	0.75	>0.99	100%	0.0%
1.50		0.510	1.790	0.050	0.510	1.700	0.060	0.75	>0.99	100%	0.0%

3.02		0.710	1.680	0.070	0.580	1.610	0.080	0.75	>0.99	82%	-18.3%
3.14		1.270	2.270	0.140	1.110	3.080	0.200	0.75	>0.99	87%	-12.6%
3.44		1.450	4.130	0.200	1.380	3.730	0.180	0.77	>0.99	95%	-4.8%
**1.28, 0.86 - 0.88**	n(CH_2) LipoPTNs	0.860	1.950	0.070	0.710	1.850	0.080	0.78	>0.99	83%	-17.4%
7.24		0.210	0.420	0.010	0.120	0.390	0.020	0.78	>0.99	57%	-42.9%
0.90		0.500	1.060	0.070	0.420	0.950	0.090	0.79	>0.99	84%	-16.0%
2.16		1.000	1.850	0.070	0.780	1.810	0.100	0.79	>0.99	78%	-22.0%
5.82		9.690	23.780	1.270	9.810	20.51	2.430	0.79	>0.99	101%	1.2%
8.48		0.060	0.170	0.000	0.050	0.220	0.000	0.79	>0.99	83%	-16.7%
7.46		0.170	0.420	0.020	0.140	0.500	0.040	0.80	>0.99	82%	-17.6%
7.74		0.050	0.460	0.000	0.020	0.520	0.000	0.80	>0.99	40%	-60.0%
0.92		0.390	0.910	0.030	0.330	0.800	0.050	0.81	>0.99	85%	-15.4%
**3.98**	Phosphoetha nolamine	2.590	12.39	0.480	2.080	13.59	0.340	0.81	>0.99	80%	-19.7%
2.36		1.200	1.990	0.070	0.800	3.960	0.170	0.82	>0.99	67%	-33.3%
2.52		0.640	1.800	0.030	0.480	1.630	0.020	0.82	>0.99	75%	-25.0%
1.08		0.270	0.630	0.010	0.250	0.740	0.030	0.83	>0.99	93%	-7.4%
1.24		1.040	2.830	0.150	0.920	2.170	0.220	0.83	>0.99	88%	-11.5%
3.20		1.570	3.300	0.200	1.350	3.990	0.260	0.83	>0.99	86%	-14.0%
7.40		0.210	0.530	0.030	0.180	0.620	0.030	0.83	>0.99	86%	-14.3%
7.70		0.270	0.590	0.040	0.160	0.600	0.030	0.83	>0.99	59%	-40.7%
4.08		1.640	7.190	0.280	2.100	7.480	0.490	0.84	>0.99	128%	28.0%
7.00		0.130	0.750	0.020	0.110	0.890	0.010	0.84	>0.99	85%	-15.4%
7.86		1.060	5.650	0.170	0.830	6.060	0.120	0.84	>0.99	78%	-21.7%
0.98 - 1.03	L-Valine	0.270	0.590	0.020	0.250	0.730	0.030	0.85	>0.99	93%	-7.4%
0.98 - 1.03		6.610	16.020	0.800	6.680	13.88	1.590	0.85	>0.99	101%	1.1%
5.86		4.510	10.840	0.490	4.600	9.570	1.050	0.85	>0.99	102%	2.0%
**1.32, 4.10 - 4.12**	Lactic_acid	0.510	1.300	0.030	0.400	1.370	0.040	0.86	>0.99	78%	-21.6%
8.36		0.030	0.170	0.000	0.030	0.210	0.010	0.86	>0.99	100%	0.0%
5.88		3.270	7.950	0.310	3.360	7.030	0.720	0.87	>0.99	103%	2.8%
8.54		0.070	0.400	0.010	0.040	0.530	0.000	0.87	>0.99	57%	-42.9%
5.92		1.550	3.780	0.050	1.610	3.480	0.250	0.88	>0.99	104%	3.9%
6.94		0.080	0.820	0.000	0.080	0.740	0.000	0.88	>0.99	100%	0.0%
**7.34 - 7.38**	Phenylalanine	0.270	0.660	0.020	0.260	0.560	0.020	0.88	>0.99	96%	-3.7%
7.04		0.140	0.550	0.010	0.130	1.000	0.000	0.89	>0.99	93%	-7.1%
7.22		0.540	1.270	0.040	0.360	1.910	0.070	0.89	>0.99	67%	-33.3%
6.92		0.140	0.430	0.010	0.120	0.430	0.020	0.91	>0.99	86%	-14.3%
7.30		0.470	1.230	0.050	0.360	1.570	0.060	0.91	>0.99	77%	-23.4%
1.30		0.480	1.100	0.000	0.330	1.170	0.000	0.92	>0.99	69%	-31.3%
**6.88, 7.18 - 7.20**	Tyrosine	0.150	0.510	0.020	0.130	0.510	0.010	0.93	>0.99	87%	-13.3%
7.50		0.130	0.520	0.000	0.080	0.420	0.010	0.94	>0.99	62%	-38.5%
5.90		2.150	5.240	0.130	2.240	4.730	0.410	0.95	>0.99	104%	4.2%
7.58		0.780	4.960	0.090	0.510	5.570	0.070	0.95	>0.99	65%	-34.6%
1.06		0.210	0.500	0.010	0.210	0.510	0.010	0.96	>0.99	100%	0.0%
1.36		0.720	2.050	0.040	0.570	1.830	0.050	0.96	>0.99	79%	-20.8%
7.56		1.180	6.970	0.160	0.790	7.650	0.120	0.96	>0.99	67%	-33.1%
5.94		1.140	2.740	0.010	1.150	2.610	0.130	0.97	>0.99	101%	0.9%

7.28		0.370	0.990	0.020	0.230	1.490	0.030	0.98	>0.99	62%	-37.8%
7.32		0.220	0.710	0.020	0.180	0.680	0.010	0.98	>0.99	82%	-18.2%
7.54		0.400	2.270	0.050	0.230	2.690	0.030	0.98	>0.99	58%	-42.5%
7.64		0.590	3.930	0.090	0.390	5.100	0.080	0.98	>0.99	66%	-33.9%
6.90		0.130	0.370	0.010	0.130	0.370	0.020	>0.99	>0.99	100%	0.0%
7.02		0.270	0.470	0.050	0.180	0.480	0.030	>0.99	>0.99	67%	-33.3%
7.20		0.450	1.060	0.080	0.300	1.170	0.050	>0.99	>0.99	67%	-33.3%
**7.84 - 7.82, 7.08**	L_Histidine	1.240	7.610	0.270	0.880	8.780	0.140	>0.99	>0.99	71%	-29.0%
8.40		0.010	0.050	0.000	0.010	0.140	0.000	>0.99	>0.99	100%	0.0%
8.66		0.010	0.020	0.000	0.010	0.040	0.000	>0.99	>0.99	100%	0.0%
**Trainees**											
**Chemical Shift (1H ppm)**	**Metabolite**	**Before**	**max**	**mim**	**After**	**max**	**mim**	**P value**	**q value**	**#**	**&**
**7.08*, 2.68**	**L_Anserine+ histamine + 3-methylhistidine**	**0.120**	**0.320**	**0.050**	**0.360**	**2.580**	**0.020**	**0.01**	**>0.99**	**300%**	**200.0%**
**9.30**		**0.020**	**0.040**	**0.000**	**0.010**	**0.020**	**0.000**	**0.03**	**>0.99**	**50%**	**-50.0%**
8.20		0.040	0.090	0.000	0.080	0.100	0.010	0.06	>0.99	200%	100.0%
2.56		1.090	1.970	0.440	1.530	2.840	0.470	0.10	>0.99	140%	40.4%
7.28		0.290	1.040	0.080	0.410	1.070	0.100	0.12	>0.99	141%	41.4%
7.30		0.310	0.890	0.150	0.460	1.040	0.110	0.13	>0.99	148%	48.4%
6.66		0.030	0.130	0.010	0.020	0.100	0.000	0.15	>0.99	67%	-33.3%
2.66		0.390	0.650	0.070	0.530	1.020	0.050	0.17	>0.99	136%	35.9%
7.24		0.110	0.430	0.040	0.190	0.450	0.030	0.18	>0.99	173%	72.7%
7.68		0.160	0.380	0.040	0.310	0.510	0.070	0.18	>0.99	194%	93.8%
3		0.310	0.860	0.060	0.480	0.710	0.070	0.23	>0.99	155%	54.8%
8.84		0.080	0.190	0.000	0.060	0.440	0.010	0.23	>0.99	75%	-25.0%
4.08		2.020	5.310	0.860	3.140	5.140	0.650	0.24	>0.99	155%	55.4%
8.1		0.050	0.150	0.000	0.060	0.330	0.010	0.24	>0.99	120%	20.0%
4.1		0.340	0.760	0.060	0.420	0.620	0.150	0.25	>0.99	124%	23.5%
2.78		0.260	0.600	0.030	0.370	0.480	0.050	0.27	>0.99	142%	42.3%
2.98		0.220	0.710	0.040	0.330	0.510	0.040	0.27	>0.99	150%	50.0%
7.22		0.450	1.320	0.150	0.630	1.360	0.160	0.27	>0.99	140%	40.0%
2.36		0.870	2.860	0.290	1.230	2.910	0.300	0.28	>0.99	141%	41.4%
2.64		0.390	0.640	0.030	0.430	0.760	0.010	0.28	>0.99	110%	10.3%
2.7		0.460	1.570	0.130	0.570	1.340	0.190	0.28	>0.99	124%	23.9%
3.7		2.340	4.900	1.150	3.110	3.980	1.360	0.28	>0.99	133%	32.9%
2.52		0.500	0.950	0.110	0.640	0.770	0.090	0.29	>0.99	128%	28.0%
2.62		0.400	0.660	0.040	0.450	0.760	0.020	0.29	>0.99	113%	12.5%
3.12		0.960	2.630	0.450	1.200	1.810	0.290	0.29	>0.99	125%	25.0%
**1.00, 0.94**	L_Isoleucine	0.140	0.420	0.010	0.250	0.350	0.030	0.31	>0.99	179%	78.6%
3.06		31.560	43.320	10.810	35.380	53.470	12.730	0.32	>0.99	112%	12.1%
8.86		0.060	0.190	0.000	0.050	0.420	0.010	0.32	>0.99	83%	-16.7%
2.3		0.530	1.460	0.170	0.780	1.180	0.190	0.33	>0.99	147%	47.2%
2.76		0.260	0.630	0.020	0.370	0.560	0.030	0.33	>0.99	142%	42.3%
2.96		0.180	0.680	0.010	0.270	0.440	0.020	0.35	>0.99	150%	50.0%
3.66, 3.26		3.280	5.040	1.120	3.690	4.960	1.430	0.35	>0.99	113%	12.5%

7.38		1.010	2.090	0.330	1.170	2.240	0.360	0.35	>0.99	116%	15.8%
7.5		0.100	0.300	0.020	0.150	0.290	0.020	0.35	>0.99	150%	50.0%
8.22		0.030	0.100	0.010	0.060	0.110	0.010	0.35	>0.99	200%	100.0%
7.04		0.170	0.480	0.000	0.140	0.320	0.000	0.36	>0.99	82%	-17.6%
**2.54 - 2.53, 2.5, 2.65, 2.68**	Citrate	1.200	1.570	0.300	1.280	1.690	0.320	0.38	>0.99	107%	6.7%
7.46		0.160	0.360	0.010	0.210	0.360	0.040	0.39	>0.99	131%	31.3%
1.06		0.170	0.460	0.000	0.270	0.350	0.020	0.40	>0.99	159%	58.8%
1.08		0.180	0.550	0.010	0.300	0.410	0.040	0.40	>0.99	167%	66.7%
2.06		1.300	2.480	0.420	1.500	2.110	0.350	0.40	>0.99	115%	15.4%
7.44		0.460	1.090	0.130	0.570	1.110	0.180	0.40	>0.99	124%	23.9%
2.28		0.860	2.130	0.250	1.110	1.780	0.290	0.41	>0.99	129%	29.1%
2.48		0.810	1.280	0.260	0.890	1.180	0.180	0.41	>0.99	110%	9.9%
2.82		0.220	0.620	0.020	0.320	0.450	0.060	0.41	>0.99	145%	45.5%
3.3		1.240	2.380	0.320	1.440	4.230	0.420	0.41	>0.99	116%	16.1%
3.68		2.520	4.880	0.910	2.670	3.950	1.140	0.41	>0.99	106%	6.0%
7.36		0.590	1.480	0.140	0.770	1.400	0.190	0.41	>0.99	131%	30.5%
7.96		0.080	0.280	0.010	0.130	0.430	0.020	0.41	>0.99	163%	62.5%
8.48		0.040	0.120	0.020	0.040	0.170	0.010	0.42	>0.99	100%	0.0%
9.14		0.070	0.200	0.000	0.050	0.430	0.000	0.42	>0.99	71%	-28.6%
2.74		1.240	2.250	0.450	1.470	1.860	0.460	0.43	>0.99	119%	18.5%
2.8		0.330	0.760	0.070	0.440	0.750	0.100	0.43	>0.99	133%	33.3%
**4.04, 3.03 - 3.04**	Creatinine	0.930	2.530	0.200	1.170	1.800	0.610	0.43	>0.99	126%	25.8%
7.4		0.160	0.430	0.050	0.210	0.590	0.040	0.43	>0.99	131%	31.3%
7.42		0.380	0.970	0.110	0.470	0.950	0.130	0.43	>0.99	124%	23.7%
**7.34 - 7.38**	Phenylalanine	0.200	0.530	0.050	0.280	0.510	0.070	0.45	>0.99	140%	40.0%
1.1		0.170	0.680	0.020	0.340	0.460	0.060	0.46	>0.99	200%	100.0%
3.38		0.450	1.060	0.100	0.530	0.860	0.180	0.46	>0.99	118%	17.8%
3.48		0.550	1.170	0.090	0.610	0.850	0.190	0.46	>0.99	111%	10.9%
3.62		1.200	2.550	0.530	1.350	1.800	0.550	0.46	>0.99	113%	12.5%
3.64		1.810	3.370	0.810	1.910	2.550	0.840	0.46	>0.99	106%	5.5%
3.78		2.350	4.170	0.830	2.640	3.800	1.250	0.46	>0.99	112%	12.3%
3.82		1.430	2.970	0.380	1.580	2.360	0.680	0.46	>0.99	110%	10.5%
3.86		1.500	2.930	0.440	1.650	2.150	0.690	0.46	>0.99	110%	10.0%
3.88		1.140	2.490	0.300	1.290	1.730	0.570	0.46	>0.99	113%	13.2%
3.9		1.020	2.280	0.300	1.200	1.940	0.460	0.46	>0.99	118%	17.6%
**4.06**	Choline	7.660	10.490	2.800	8.550	13.760	2.760	0.46	>0.99	112%	11.6%
0.98 - 1.03	L-Valine	0.230	0.580	0.040	0.340	0.490	0.090	0.48	>0.99	148%	47.8%
0.98 - 1.03		0.990	2.040	0.260	1.100	1.820	0.420	0.48	>0.99	111%	11.1%
7.18		0.130	0.430	0.040	0.210	0.540	0.020	0.48	>0.99	162%	61.5%
2.08		0.960	2.120	0.310	1.250	1.640	0.270	0.50	>0.99	130%	30.2%
2.92		0.410	1.010	0.090	0.500	0.690	0.100	0.50	>0.99	122%	22.0%
3.16		1.370	2.070	0.520	1.550	2.190	0.600	0.50	>0.99	113%	13.1%
3.56		0.840	2.200	0.330	0.960	1.400	0.360	0.50	>0.99	114%	14.3%
3.6		1.250	2.460	0.470	1.300	1.720	0.530	0.51	>0.99	104%	4.0%
7.32		0.130	0.390	0.050	0.170	0.670	0.030	0.51	>0.99	131%	30.8%
2.32		0.430	1.140	0.090	0.610	0.770	0.090	0.52	>0.99	142%	41.9%

2.42		0.480	0.980	0.130	0.630	0.770	0.100	0.52	>0.99	131%	31.3%
3.24		2.850	5.720	0.730	2.390	4.680	0.990	0.52	>0.99	84%	-16.1%
3.84		1.940	4.300	0.550	2.090	2.750	0.950	0.52	>0.99	108%	7.7%
2.1		0.700	1.620	0.230	0.950	1.220	0.180	0.53	>0.99	136%	35.7%
2.9		0.330	0.890	0.050	0.410	0.690	0.060	0.54	>0.99	124%	24.2%
2.34		0.450	1.170	0.080	0.630	0.780	0.090	0.56	>0.99	140%	40.0%
**2.44, 2.14, 3.76**	L_Glutamine	0.670	1.140	0.190	0.740	0.910	0.150	0.56	>0.99	110%	10.4%
4.02		0.790	1.660	0.170	0.900	1.450	0.410	0.56	>0.99	114%	13.9%
0.92		0.310	0.900	0.090	0.460	0.600	0.140	0.58	>0.99	148%	48.4%
2.26		0.770	1.860	0.220	1.000	1.390	0.260	0.58	>0.99	130%	29.9%
2.84		0.450	0.900	0.120	0.560	0.710	0.150	0.58	>0.99	124%	24.4%
3.02		0.690	1.610	0.140	0.780	1.370	0.160	0.58	>0.99	113%	13.0%
**3.42**	L_Carnitine	0.930	1.280	0.140	0.970	1.700	0.380	0.58	>0.99	104%	4.3%
3.46		1.050	2.080	0.380	1.290	2.350	0.440	0.58	>0.99	123%	22.9%
3.72		2.870	10.100	1.020	3.370	10.780	1.360	0.58	>0.99	117%	17.4%
**7.84 - 7.82, 7.08**	L_Histidine	0.940	2.560	0.160	0.590	7.430	0.230	0.58	>0.99	63%	-37.2%
2.12		0.660	1.540	0.190	0.830	1.060	0.160	0.59	>0.99	126%	25.8%
2.88		0.460	1.120	0.100	0.500	0.710	0.200	0.59	>0.99	109%	8.7%
3.1		0.510	1.170	0.100	0.540	1.030	0.170	0.59	>0.99	106%	5.9%
6.94		0.050	0.160	0.010	0.050	0.530	0.000	0.61	>0.99	100%	0.0%
2.18		0.750	1.730	0.210	0.880	1.450	0.200	0.62	>0.99	117%	17.3%
2.38		0.570	1.230	0.150	0.670	0.880	0.130	0.62	>0.99	118%	17.5%
3.18		1.030	2.160	0.290	1.230	2.240	0.390	0.62	>0.99	119%	19.4%
1.24		0.910	1.940	0.180	1.260	1.930	0.510	0.64	>0.99	138%	38.5%
2		0.700	1.630	0.130	0.770	1.140	0.130	0.64	>0.99	110%	10.0%
**2.04**	N_Acetyl_L_glutamic_acid	0.920	2.400	0.370	1.120	1.460	0.260	0.64	>0.99	122%	21.7%
2.24		0.520	1.240	0.120	0.720	0.970	0.200	0.64	>0.99	138%	38.5%
2.46		1.000	1.720	0.280	1.040	1.360	0.230	0.64	>0.99	104%	4.0%
3.14		1.230	2.540	0.340	1.400	2.190	0.520	0.64	>0.99	114%	13.8%
3.32		1.060	2.630	0.390	1.130	2.650	0.300	0.64	>0.99	107%	6.6%
3.34		0.640	1.530	0.140	0.670	1.830	0.220	0.64	>0.99	105%	4.7%
3.36		0.730	1.510	0.250	0.840	1.470	0.270	0.64	>0.99	115%	15.1%
3.54		0.970	3.040	0.340	1.120	1.500	0.420	0.64	>0.99	115%	15.5%
3.8		2.100	3.940	0.710	2.210	3.300	1.040	0.64	>0.99	105%	5.2%
**3.96**	3_Methylhistidine	1.740	3.200	0.720	1.750	4.770	0.790	0.64	>0.99	101%	0.6%
7.74		0.030	0.140	0.000	0.040	0.250	0.020	0.64	>0.99	133%	33.3%
7.76		0.040	0.160	0.020	0.050	0.270	0.020	0.64	>0.99	125%	25.0%
**0.86 - 0.88**	n(CH3) LipoPTNs	0.180	0.400	0.040	0.250	0.310	0.080	0.65	>0.99	139%	38.9%
2.5		0.580	1.130	0.130	0.640	0.820	0.080	0.65	>0.99	110%	10.3%
7.66		0.400	1.090	0.080	0.270	2.460	0.110	0.65	>0.99	68%	-32.5%
2.02		0.510	1.380	0.080	0.630	0.820	0.060	0.66	>0.99	124%	23.5%
**8.46**	Formate	0.020	0.060	0.010	0.020	0.090	0.010	0.66	>0.99	100%	0.0%
0.9		0.480	1.120	0.130	0.590	0.850	0.220	0.68	>0.99	123%	22.9%
0.96		0.260	1.030	0.070	0.380	0.540	0.100	0.68	>0.99	146%	46.2%
7.02		0.190	0.870	0.090	0.220	0.590	0.100	0.68	>0.99	116%	15.8%
8.36		0.030	0.100	0.010	0.020	0.110	0.010	0.68	>0.99	67%	-33.3%

**6.88, 7.18 - 7.20**	Tyrosine	0.090	0.300	0.040	0.110	0.350	0.020	0.69	>0.99	122%	22.2%
8.54		0.040	0.130	0.010	0.050	0.400	0.010	0.69	>0.99	125%	25.0%
8.56		0.030	0.050	0.010	0.030	0.130	0.010	0.69	>0.99	100%	0.0%
1.26		0.530	1.710	0.050	0.710	1.190	0.090	0.70	>0.99	134%	34.0%
1.84		0.330	1.130	0.030	0.480	0.720	0.020	0.70	>0.99	145%	45.5%
4		0.940	2.050	0.230	0.970	2.510	0.450	0.70	>0.99	103%	3.2%
7.86		0.750	2.050	0.230	0.710	5.210	0.280	0.70	>0.99	95%	-5.3%
1.2		0.390	1.570	0.080	0.640	1.050	0.170	0.71	>0.99	164%	64.1%
7		0.050	0.210	0.010	0.050	0.540	0.000	0.71	>0.99	100%	0.0%
1.22		0.500	1.780	0.120	0.660	1.050	0.150	0.72	>0.99	132%	32.0%
1.82		0.320	1.080	0.040	0.430	0.700	0.010	0.72	>0.99	134%	34.4%
1.98		0.420	1.160	0.020	0.550	0.750	0.020	0.72	>0.99	131%	31.0%
3.4		0.390	1.110	0.030	0.430	0.640	0.120	0.72	>0.99	110%	10.3%
7.6		0.070	0.230	0.000	0.060	0.610	0.000	0.74	>0.99	86%	-14.3%
1.9		0.420	1.270	0.020	0.530	0.700	0.030	0.75	>0.99	126%	26.2%
1.96		0.540	1.460	0.100	0.700	0.920	0.060	0.75	>0.99	130%	29.6%
2.16		1.020	1.920	0.280	1.070	1.380	0.250	0.76	>0.99	105%	4.9%
2.22		0.510	1.440	0.110	0.750	1.110	0.220	0.76	>0.99	147%	47.1%
3.2		1.700	3.390	0.470	1.700	2.780	0.680	0.76	>0.99	100%	0.0%
3.44		1.390	2.420	0.260	1.400	2.960	0.550	0.76	>0.99	101%	0.7%
3.5		0.670	1.850	0.210	0.770	1.100	0.290	0.76	>0.99	115%	14.9%
**3.98**	Phosphoethanolamine	2.280	4.930	0.670	1.750	11.860	0.740	0.76	>0.99	77%	-23.2%
7.7		0.220	0.390	0.070	0.250	0.620	0.050	0.76	>0.99	114%	13.6%
8.34		0.090	0.140	0.020	0.090	0.270	0.030	0.76	>0.99	100%	0.0%
1.92		0.610	1.640	0.120	0.710	0.930	0.100	0.78	>0.99	116%	16.4%
1.34		0.760	1.690	0.180	0.920	1.230	0.180	0.81	>0.99	121%	21.1%
1.68		0.360	2.030	0.050	0.560	0.800	0.020	0.81	>0.99	156%	55.6%
1.94		0.780	1.900	0.200	0.920	1.210	0.160	0.81	>0.99	118%	17.9%
3.28		4.150	9.670	1.400	4.290	8.360	1.720	0.81	>0.99	103%	3.4%
2.2		0.590	1.690	0.140	0.830	1.380	0.210	0.82	>0.99	141%	40.7%
6.98		0.080	0.200	0.010	0.070	0.330	0.000	0.82	>0.99	88%	-12.5%
1.64		0.270	1.010	0.020	0.430	0.730	0.010	0.83	>0.99	159%	59.3%
1.66		0.330	1.190	0.040	0.470	0.750	0.010	0.83	>0.99	142%	42.4%
**3.22**	n(CH3)3 Choline	1.570	3.260	0.540	1.390	3.370	0.510	0.83	>0.99	89%	-11.5%
3.74		1.590	5.090	0.560	2.200	8.210	0.850	0.83	>0.99	138%	38.4%
5.8		14.390	24.870	7.170	16.010	23.330	6.960	0.83	>0.99	111%	11.3%
8.52		0.050	0.150	0.010	0.040	0.550	0.010	0.83	>0.99	80%	-20.0%
**3.58, 4.26, 1.34**	Threonine	1.890	4.280	0.760	2.050	2.680	0.700	0.85	>0.99	108%	8.5%
6.92		0.110	0.270	0.030	0.110	0.430	0.030	0.85	>0.99	100%	0.0%
7.72		0.100	0.510	0.040	0.120	0.300	0.030	0.85	>0.99	120%	20.0%
**1.28**	n(CH2) LipoPTNs	0.520	1.900	0.060	0.760	1.070	0.160	0.88	>0.99	146%	46.2%
**1.32, 4.10 - 4.12**	Lactic_acid	0.250	1.320	0.030	0.590	1.230	0.070	0.88	>0.99	236%	136.0%
5.94		1.020	2.280	0.480	1.460	2.180	0.390	0.88	>0.99	143%	43.1%
1.36		0.490	1.560	0.080	0.760	1.130	0.150	0.90	>0.99	155%	55.1%
**1.48 - 1.46**	Alanine	0.460	1.390	0.110	0.680	0.930	0.070	0.90	>0.99	148%	47.8%

1.5		0.390	1.460	0.090	0.690	1.030	0.070	0.90	>0.99	177%	76.9%
5.78		15.110	25.280	7.650	16.670	24.270	7.500	0.90	>0.99	110%	10.3%
5.82		10.580	18.880	5.370	12.070	17.610	5.080	0.90	>0.99	114%	14.1%
5.84		7.110	12.850	3.680	8.220	12.010	3.360	0.90	>0.99	116%	15.6%
5.88		3.430	6.400	1.780	4.130	6.060	1.570	0.90	>0.99	120%	20.4%
1.3		0.250	1.340	0.030	0.510	0.830	0.040	0.92	>0.99	204%	104.0%
2.94		0.520	0.960	0.150	0.490	0.840	0.120	0.92	>0.99	94%	-5.8%
5.86		4.760	8.760	2.500	5.590	8.190	2.210	0.92	>0.99	117%	17.4%
6.9		0.110	0.270	0.030	0.120	0.280	0.040	0.92	>0.99	109%	9.1%
7.2		0.280	0.700	0.080	0.270	0.520	0.070	0.92	>0.99	96%	-3.6%
7.56		0.860	2.460	0.180	0.620	6.700	0.210	0.92	>0.99	72%	-27.9%
7.58		0.560	1.670	0.090	0.370	4.900	0.130	0.92	>0.99	66%	-33.9%
7.64		0.460	1.230	0.060	0.250	3.700	0.110	0.92	>0.99	54%	-45.7%
5.9		2.150	4.260	1.120	2.760	4.070	0.970	0.95	>0.99	128%	28.4%
2.58		0.910	1.930	0.090	0.680	1.730	0.120	0.97	>0.99	75%	-25.3%
5.92		1.490	3.080	0.740	2.000	2.980	0.630	0.97	>0.99	134%	34.2%
6.86		0.070	0.230	0.030	0.080	0.430	0.020	0.99	>0.99	114%	14.3%
6.96		0.040	0.140	0.010	0.040	0.150	0.000	>0.99	>0.99	100%	0.0%
7.06		0.120	2.420	0.010	0.110	0.400	0.020	>0.99	>0.99	92%	-8.3%
7.54		0.300	0.760	0.080	0.220	2.060	0.070	>0.99	>0.99	73%	-26.7%
8.4		0.010	0.050	0.000	0.010	0.030	0.000	>0.99	>0.99	100%	0.0%
8.66		0.010	0.010	0.000	0.000	0.020	0.000	>0.99	>0.99	0%	-100.0%

Table S5. Peak report from HSQC submitted to COLMARm. Session ID 3307-SJKHh6VhYX from https://spin.ccic.osu.edu/index.php/colmarm/index. Default parameters with classical peak peaking, no peak fitting, and no spectral referencing.

Proton	Proton2	Amplitude	compound_name
1.278	30.93	312398.6	3_Hydroxyisovaleric_acid
1.311	31.52	90736.51	Pimelic_acid
1.365	29.64	125392.1	Alpha_hydroxyisobutyric_acid
2.067	25.01	225272.7	Acetyl_phosphate
2.186	40.11	128355.4	Pimelic_acid
2.373	52.29	128495.7	3_Hydroxyisovaleric_acid
2.447	45.57	99317.12	L_Carnitine
2.462	34.04	112533	Thioacetamide
2.733	37.45	199543.5	Dimethylamine
2.839	29.96	77829.69	Methylguanidine
3.052	27.41	96554.16	L_Cysteine
3.053	33	6705530	Creatinine
3.16	44.17	144918.8	Ethanolamine
3.205	56.67	596712.6	L_Carnitine
3.243	28.03	155958	1,9_Dimethyluric_acid
3.257	27.69	167945.3	3_Methylhistidine
3.26	76.85	91197.41	D-Glucuronic_acid
3.274	62.39	881099	Trimethylamine_N_oxide
3.276	30.75	140097.6	1,9_Dimethyluric_acid
3.277	76.6	86462.87	D_Glucuronate
3.304	43.51	79037.37	Phenethylamine
3.316	76.46	81670.97	scyllo_Inositol
3.416	72.63	110497.1	L_Carnitine
3.458	46.3	139979.8	4_Hydroxyphenylacetic_acid
3.505	78.57	152706.6	D-Glucuronic_acid
3.523	78.32	170140.5	D_Glucuronate
3.523	70.06	192832.3	Choline
3.531	74.7	219817.4	D_Glucuronate
3.571	44.36	542531.9	Glycine
3.582	74.17	184072.1	D-Glucuronic_acid
3.634	65.34	423059.9	meso_Erythritol
3.656	65.26	620592.3	Gluconic_acid
3.666	45.2	233554.3	Phenylacetylglycine
3.692	65.61	552605.5	L_Iditol
3.696	74.84	357294.8	Adonitol
3.723	65	289379.3	L_Gulonolactone
3.732	34.72	728345.4	3_Methylhistidine
3.738	78.99	99575.06	D_Glucuronate
3.758	74	250763.8	L_Iditol
3.779	65.03	431763.6	Adonitol
3.78	74.33	350835.4	6_Phosphogluconic_acid

3.804	47.37	264887.1	Guanidineacetic_acid
3.809	72.96	190513.3	L_Gulonolactone
3.822	73.23	190002.7	6_Phosphogluconic_acid
3.834	60.53	139936.8	Ethanolamine
3.851	74.18	149620.1	L_Iditol
3.892	58.51	103163.1	Homovanillic_acid
3.908	71.78	165880	D_Galacturonic_acid
3.92	63.95	135083	Glycolate
3.966	56.41	359306.9	3_Methylhistidine
3.977	46.62	2038834	Hippuric_acid
4.014	71.82	175423.1	D_Galactose
4.046	58.98	1784833	Creatinine
4.049	73.2	184901.1	D_Galactose
4.082	74.14	110930.1	D_Saccharate
4.105	76.07	163710.7	L_Gulonolactone
4.144	76.64	171061.8	Gluconic_acid
4.25	73.5	77888.59	D_Galacturonic_acid
5.25	95.07	111086.4	D_Galacturonic_acid
6.867	118.31	94740.85	4_Hydroxyphenylacetic_acid
7.101	126.94	300946.3	3_Methylhistidine
7.372	131.74	282071.8	Phenylacetylglycine
7.431	132.06	209999.6	Phenethylamine
7.561	131.6	819538.3	Hippuric_acid
7.648	135.03	273945.2	Hippuric_acid
7.838	130.04	854631.6	Hippuric_acid
8.002	141.55	121672.8	3_Methylhistidine

Table S6. Categorical variables of pilots. Chi-Square test was performed to the proportion the categorical variables was equal between Trainers and Instructors. *Values less than p < 0.05.

Variable	Trainees (n = 12)	Instructors (n = 20)	p-value
**POSITION IN A-29**			
**P1**	11	10	**0.016***
**P2**	1	10	
**PERIOD of FLIGHT**			
**Morning**	6	7	0.40
**Afternoon**	6	13	
**PHYSICAL ACTIVITY**			
**Yes**	7	19	**0.01***
**No**	5	1	
**SMOKING**			
**Yes**	1	3	0.58
**No**	11	17	
**HYPERTENSION**			
**Yes**	0	0	-
**No**	20	11	
**DIABETES**			
**Yes**	0	1	0.45
**No**	11	19	
**SUPPLEMENT**			
**Yes**	5	9	0.85
**No**	7	11	

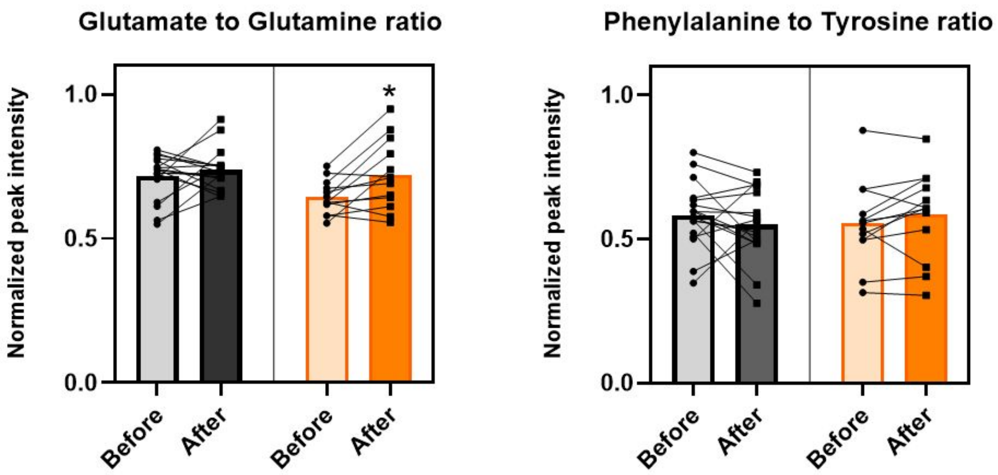

Figure S7. Metabolic ratios of serum. Instructors (Gray) and Trainees (orange). Paired analysis by Wilcoxon nonparametric test. Bars represent median. *(p<0.05).

Traineers													
R	Mean Corpuscular Volume (fL)	Segmented (mm3)	0.98 - L-Valine	1 - Leucine	1.04 - Isobutyrate	1.2 - n(CH2) LipoPTNs	1.32 - Lactate	1.46 - Alanine	1.9 - Acetate	5.22 - α-Glucose	5.24 - Lipids	7.18 -Tyrosine	7.84 - Histidine
Mean Corpuscular Volume (fL)	1,000	-0,320	-0,119	-0,070	-0,092	-0,236	0,047	-0,068	-0,186	0,250	-0,042	-0,013	-0,148
Segmented (mm3)	-0,320	1,000	-0,214	-0,177	-0,237	0,572	-0,013	-0,403	-0,390	0,273	0,323	-0,584	0,027
0.98 - L-Valine	-0,119	-0,214	1,000	0,965	0,943	-0,157	-0,045	0,689	0,710	-0,387	0,079	0,421	0,497
1 - Leucine	-0,070	-0,177	0,965	1,000	0,941	-0,131	0,052	0,623	0,616	-0,372	0,107	0,295	0,475
1.04 - Isobutyrate	-0,092	-0,237	0,943	0,941	1,000	-0,289	0,122	0,700	0,558	-0,315	-0,013	0,247	0,321
1.2 - n(CH2) LipoPTNs	-0,236	0,572	-0,157	-0,131	-0,289	1,000	-0,174	-0,346	-0,148	-0,197	0,162	-0,183	0,191
1.32 - Lactate	0,047	-0,013	-0,045	0,052	0,122	-0,174	1,000	0,094	-0,287	-0,257	-0,570	-0,183	-0,412
1.46 - Alanine	-0,068	-0,403	0,689	0,623	0,700	-0,346	0,094	1,000	0,657	-0,312	-0,151	0,362	0,323
1.9 - Acetate	-0,186	-0,390	0,710	0,616	0,558	-0,148	-0,287	0,657	1,000	-0,423	0,122	0,729	0,766
5.22 - α-Glucose	0,250	0,273	-0,387	-0,372	-0,315	-0,197	-0,257	-0,312	-0,423	1,000	0,615	-0,617	-0,238
5.24 - Lipids	-0,042	0,323	0,079	0,107	-0,013	0,162	-0,570	-0,151	0,122	0,615	1,000	-0,267	0,421
7.18 -Tyrosine	-0,013	-0,584	0,421	0,295	0,247	-0,183	-0,183	0,362	0,729	-0,617	-0,267	1,000	0,429
7.84 - Histidine	-0,148	0,027	0,497	0,475	0,321	0,191	-0,412	0,323	0,766	-0,238	0,421	0,429	1,000
P-value	Mean Corpuscular Volume (fL)	Segmented (mm3)	0.98 - L-Valine	1 - Leucine	1.04 - Isobutyrate	1.2 - n(CH2) LipoPTNs	1.32 - Lactate	1.46 - Alanine	1.9 - Acetate	5.22 - α-Glucose	5.24 - Lipids	7.18 -Tyrosine	7.84 - Histidine
Mean Corpuscular Volume (fL)		0,127	0,580	0,745	0,671	0,266	0,827	0,751	0,385	0,238	0,844	0,952	0,489
Segmented (mm3)	0,127	1,000	0,316	0,409	0,266	0,003	0,952	0,051	0,059	0,197	0,124	0,003	0,900
0.98 - L-Valine	0,580	0,316	1,000	0,000	0,000	0,465	0,834	0,000	0,000	0,062	0,713	0,041	0,014
1 - Leucine	0,745	0,409	0,000	1,000	0,000	0,541	0,809	0,001	0,001	0,073	0,619	0,162	0,019
1.04 - Isobutyrate	0,671	0,266	0,000	0,000	1,000	0,171	0,571	0,000	0,005	0,134	0,952	0,245	0,126
1.2 - n(CH2) LipoPTNs	0,266	0,003	0,465	0,541	0,171	1,000	0,416	0,098	0,491	0,355	0,450	0,391	0,371
1.32 - Lactate	0,827	0,952	0,834	0,809	0,571	0,416	1,000	0,662	0,174	0,225	0,004	0,393	0,045
1.46 - Alanine	0,751	0,051	0,000	0,001	0,000	0,098	0,662	1,000	0,000	0,138	0,480	0,082	0,123
1.9 - Acetate	0,385	0,059	0,000	0,001	0,005	0,491	0,174	0,000	1,000	0,039	0,571	0,000	0,000
5.22 - α-Glucose	0,238	0,197	0,062	0,073	0,134	0,355	0,225	0,138	0,039	1,000	0,001	0,001	0,262
5.24 - Lipids	0,844	0,124	0,713	0,619	0,952	0,450	0,004	0,480	0,571	0,001	1,000	0,207	0,041
7.18 -Tyrosine	0,952	0,003	0,041	0,162	0,245	0,391	0,393	0,082	0,000	0,001	0,207	1,000	0,037
7.84 - Histidine	0,489	0,900	0,014	0,019	0,126	0,371	0,045	0,123	0,000	0,262	0,041	0,037	1,000

Instructores										
R	Lymphocytes (mm3)	0.84 n(CH_3)LipoPTNs	1.2 - n(CH2)LipoPTNs	1.32 - Lactate	1.42 - Alanine	2.32 - Glutamate	2.44 - Glutamine	3.22 - n(CH3)3 Choline	3.58 - Threonine	8.46 - Formate
Lymphocytes (mm3)	1,00	-0,51	-0,02	0,01	0,38	0,27	-0,02	-0,43	-0,09	-0,24
0.84 n(CH_3)LipoPTNs	-0,51	1,00	0,48	-0,52	-0,49	-0,07	0,28	0,95	0,52	0,51
1.2 - n(CH2)LipoPTNs	-0,02	0,48	1,00	-0,56	-0,47	0,37	0,38	0,47	0,70	0,58
1.32 - Lactate	0,01	-0,52	-0,56	1,00	0,37	-0,37	-0,51	-0,60	-0,58	-0,68
1.42 - Alanine	0,38	-0,49	-0,47	0,37	1,00	0,24	0,00	-0,43	-0,45	-0,67
2.32 - Glutamate	0,27	-0,07	0,37	-0,37	0,24	1,00	0,79	0,09	0,52	0,11
2.44 - Glutamine	-0,02	0,28	0,38	-0,51	0,00	0,79	1,00	0,45	0,73	0,35
3.22 - n(CH3)3 Choline	-0,43	0,95	0,47	-0,60	-0,43	0,09	0,45	1,00	0,63	0,58
3.58 - Threonine	-0,09	0,52	0,70	-0,58	-0,45	0,52	0,73	0,63	1,00	0,66
8.46 - Formate	-0,24	0,51	0,58	-0,68	-0,67	0,11	0,35	0,58	0,66	1,00
P-value	Lymphocytes (mm3)	0.84 n(CH_3)LipoPTNs	1.2 - n(CH2)LipoPTNs	1.32 - Lactate	1.42 - Alanine	2.32 - Glutamate	2.44 - Glutamine	3.22 - n(CH3)3 Choline	3.58 - Threonine	8.46 - Formate
Lymphocytes (mm3)		0,00	0,90	0,97	0,02	0,12	0,90	0,01	0,60	0,17
0.84 n(CH_3)LipoPTNs	0,00		0,00	0,00	0,00	0,67	0,10	0,00	0,00	0,00
1.2 - n(CH2)LipoPTNs	0,90	0,00		0,00	0,00	0,03	0,02	0,00	0,00	0,00
1.32 - Lactate	0,97	0,00	0,00		0,03	0,03	0,00	0,00	0,00	0,00
1.42 - Alanine	0,02	0,00	0,00	0,03		0,16	0,99	0,01	0,01	0,00
2.32 - Glutamate	0,12	0,67	0,03	0,03	0,16		0,00	0,62	0,00	0,53
2.44 - Glutamine	0,90	0,10	0,02	0,00	0,99	0,00		0,01	0,00	0,04
3.22 - n(CH3)3 Choline	0,01	0,00	0,00	0,00	0,01	0,62	0,01		0,00	0,00
3.58 - Threonine	0,60	0,00	0,00	0,00	0,01	0,00	0,00	0,00		0,00
8.46 - Formate	0,17	0,00	0,00	0,00	0,00	0,53	0,04	0,00	0,00	

Figure S8. Trainees and instructors have different correlations between serum metabolites and key blood parameters. The tables show R and p-values from spearman correlation. Color scale represents r correlation, highest positive r = +1, dark green, and lowest negative r = -1, dark red; blue color highlights significant correlations ($p < 0.05$).

Trainees			
	Mean Corpuscular Volume (fL)	Segmented (mm3)	5.22 - α-Glucose
Mean Corpuscular Volume (fL)	1,00	-0,32	-0,22
Segmented (mm3)	-0,32	1,00	0,65
5.22 - α-Glucose	-0,22	0,65	1,00
	Mean Corpuscular Volume (fL)	Segmented (mm3)	5.22 - α-Glucose
Mean Corpuscular Volume (fL)		0,23	0,41
Segmented (mm3)	0,23		0,01
5.22 - α-Glucose	0,41	0,01	

Instructores						
	Lymphocytes (mm3)	2.56 - Acetylcarnitine	2.66 - (R) Malic acid	3.22 - n(Ch3)3 Choline	3.28 - L-Cysteic acid	4.10 Lactate
Lymphocytes (mm3)	1,00	-0,41	-0,29	-0,14	-0,32	-0,54
2.56 - Acetylcarnitine	-0,41	1,00	0,91	0,70	0,69	0,85
2.66 - (R) Malic acid	-0,29	0,91	1,00	0,84	0,62	0,71
3.22 - n(Ch3)3 Choline	-0,14	0,70	0,84	1,00	0,70	0,59
3.28 - L-Cysteic acid	-0,32	0,69	0,62	0,70	1,00	0,80
4.10 Lactate	-0,54	0,85	0,71	0,59	0,80	1,00
	Lymphocytes (mm3)	2.56 - Acetylcarnitine	2.66 - (R) Malic acid	3.22 - n(Ch3)3 Choline	3.28 - L-Cysteic acid	4.10 Lactate
Lymphocytes (mm3)		0,05	0,17	0,53	0,12	0,01
2.56 - Acetylcarnitine	0,05		0,00	0,00	0,00	0,00
2.66 - (R) Malic acid	0,17	0,00		0,00	0,00	0,00
3.22 - n(Ch3)3 Choline	0,53	0,00	0,00		0,00	0,00
3.28 - L-Cysteic acid	0,12	0,00	0,00	0,00		0,00
4.10 Lactate	0,01	0,00	0,00	0,00	0,00	

Figure S9. Trainees and instructors have different correlations between saliva metabolites and key blood parameters. The tables show R and p-values from spearman correlation. Color scale represents r correlation, highest positive r = +1, dark green, and lowest negative r = -1, dark red; blue color highlights significant correlations ($p < 0.05$).

Traineers			
R	Mean Corpuscular Volume (fL)	Segmented (mm3)	7.08 - L_Anserine+ histamine + 3-methylhistidine
Mean Corpuscular Volume (fL)	1,00	-0,33	0,16
Segmented (mm3)	-0,33	1,00	0,40
7.08 - L_Anserine+ histamine + 3-methylhistidine	0,16	0,40	1
P-value	Mean Corpuscular Volume (fL)	Segmented (mm3)	7.08 - L_Anserine+ histamine + 3-methylhistidine
Mean Corpuscular Volume (fL)		0,133	0,486
Segmented (mm3)	0,133		0,065
7.08 - L_Anserine+ histamine + 3-methylhistidine	0,486	0,065	1,000
Instructores			
R	Lymphocytes (mm3)	8.84 - Trigonelline	2.7 - Atropine
Lymphocytes (mm3)	1,000	0,035	0,217
8.84 - Trigonelline	0,035	1,000	0,486
2.7 - Atropine	0,217	0,486	1,000
P-value	Lymphocytes (mm3)	8.84 - Trigonelline	2.7 - Atropine
Lymphocytes (mm3)		0,841	0,204
8.84 - Trigonelline	0,841		0,003
2.7 - Atropine	0,204	0,003	

Figure S10. Trainees and instructors have different correlations between urine metabolites and key blood parameters. The tables show R and p-values from spearman correlation. Color scale represents r correlation, highest positive r = +1, dark green, and lowest negative r = -1, dark red; blue color highlights significant correlations ($p < 0.05$).
